# Metabolites associated with the inclusion of atlantic GRO® seaweed supplementation as a methane mitigation strategy in the diet of replacement beef heifers

**DOI:** 10.3389/fmolb.2026.1866940

**Published:** 2026-07-29

**Authors:** Shima Borzouie, Ghader Manafiazar

**Affiliations:** Department of Animal Science and Aquaculture, Dalhousie University, Truro, NS, Canada

**Keywords:** DFI/LC–MS/MS, enteric methane emissions, metabolomics, plasma biomarkers, seaweed supplementation

## Abstract

**Introduction:**

Dietary seaweed supplementation reduces enteric methane emissions in cattle, but the metabolic mechanisms remain unclear.

**Methods:**

This study evaluated plasma metabolomic responses to Atlantic GRO® brown seaweed supplementation at 0.0%, 0.5%, 1.0%, 2.0% of diet dry matter, and replacement beef heifers were classified into low, moderate, and high methane emission groups. Plasma metabolites were analyzed using direct flow injection/liquid chromatography–tandem mass spectrometry (DFI/LC–MS/MS), and a total of 453 plasma metabolites were quantified.

**Results:**

Significant dose-dependent metabolic shifts were observed, particularly at the 1.0% and 2.0% supplementation levels (p < 0.05). Compared with the control, the 2.0% seaweed group showed alterations in valine, TG.18:0_38:6, asymmetric dimethylarginine (ADMA), C2 acylcarnitine, trimethylamine (TMA), and inosine (p < 0.05, AUC ≥ 0.75, VIP score >1, MDA > 0), mainly involving lipid and amino acid metabolism. Stronger effects occurred at higher inclusion levels (1.0% and 2.0%). Methane-emission groups also differed in metabolites associated with energy metabolism and microbial fermentation pathways, with urea and ethylmalonic acid (EMA) identified as potential biomarkers (p < 0.05, AUC ≥ 0.75, VIP score > 1, MDA > 0). Overall, 152 metabolites were significantly affected by the interaction between seaweed supplementation and methane-emission group (p < 0.05), indicating that the metabolic response depended on methane phenotype. Metabolite differences were greatest between moderate and high methane emitters, with interaction-related changes mainly involving triglycerides, phosphatidylcholines, and microbial compounds, reflecting changes in lipid metabolism, nitrogen homeostasis, and gut microbial activity. Pathway analysis revealed enrichment of arginine biosynthesis, purine metabolism, and branched-chain amino acid metabolism, particularly at higher supplementation levels.

**Discussion:**

Overall, Atlantic GRO® seaweed induced dose-dependent metabolic changes associated with methane reduction and supports the potential use of plasma metabolites as non-invasive biomarkers of enteric methane emissions in cattle.

## Introduction

Cattle production systems are major contributors to greenhouse gas (GHG) emissions within the livestock and agriculture sector, accounting for approximately 18% of global anthropogenic greenhouse gas emissions, mainly through methane and nitrous oxide (Moran and Wall, 2011). A substantial proportion of these emissions originates from enteric methane, produced during ruminal fermentation and closely linked to forage intake in ruminant animals. Indeed, digestive fermentation is a natural physiological process that enables ruminants to utilize fibrous plant materials, yet it also results in methane production. Within beef production systems, replacement heifers play a critical role and contribute substantially to enteric methane emissions due to their predominantly forage-based diets. Consequently, effective methane mitigation strategies targeting this production stage have the potential to not only reduce the environmental footprint but also to increase profitability for the industry.

Recent research has demonstrated that dietary seaweed supplementation can effectively reduce enteric methane emissions. Greater methane reductions have been reported with red seaweed species, including reductions of up to 95% in an *in vivo* study using *Asparagopsis taxiformis,* supplemented at 5.0% organic matter (Kinley et al., 2016) and up to 67% with *Asparagopsis armata* at 1.0% of diet on a dry matter intake basis, in both dairy cattle (Vijn et al., 2020) and beef cattle (Roque et al., 2019). These findings highlight the strong potential of seaweed supplementation as a methane mitigation strategy in ruminant animal production systems. Among available seaweed species, red seaweed has been the subject of extensive research, whereas brown seaweed remains comparatively underexplored.

Atlantic GRO® is a 100% natural, organic seaweed meal made from the sustainably harvested brown macroalgae *Laminaria longicruris* and *Fucus vesiculosus* from the shores of Prince Edward Island, Canada, providing a responsibly sourced, readily available resource for agricultural use. Increasing interest in the use of brown seaweed supplementation has highlighted its potential role in mitigating methane emissions from livestock (Borzouie et al., 2026; McGurrin et al., 2023; Ahmed et al., 2022; Künzel et al., 2022; Abbott et al., 2020). Notably, our previous research found that supplementation of replacement heifers’ diets with 1.0% and 2.0% of the North Atlantic Organics brown seaweed product significantly reduced methane emissions, with reductions of 8.2%–8.7% compared to the control diet (Borzouie et al., 2026). However, the biochemical and metabolic mechanisms underlying these methane-reduction effects remain poorly understood.

Metabolomics is a powerful tool to elucidate the biological mechanisms associated with methane mitigation by characterizing systemic metabolic responses in cattle (Tachibana, 2014). However, few studies have focused on cattle metabolomics resulting from methane mitigation strategies, highlighting the complexity of rumen metabolism and the need for ongoing research.

Among the limited studies, a comprehensive metabolomics approach revealed that plasma metabolites associated with enteric methane emissions in beef cattle are largely breed-specific, with only a small proportion shared across breeds (Li et al., 2024). Other studies have linked plasma metabolites to rumen methanogenesis in beef cattle fed different silage diets (Yanibada et al., 2020). Similarly, a Nuclear Magnetic Resonance (NMR) analysis in beef cattle showed that the association between metabolites and methane emissions varies by diet, with key differences in acetate, butyrate, and propionate linked to variations in emissions (Bica et al., 2022).

Plasma samples offer a real-time view of circulating metabolites that reflect ruminal fermentation, hormonal regulation, and immune function influencing methane production. Nevertheless, circulating metabolomic investigations of cattle receiving seaweed supplementation are lacking. This gap includes limited understanding of how seaweed supplementation alters rumen fermentation, host metabolism, and related biochemical pathways.

Therefore, the objective of this study was to apply a liquid chromatography–tandem mass spectrometry (LC–MS/MS)-based metabolomics approach to evaluate the independent and combined effects of Atlantic GRO® seaweed supplementation and methane emission categories on the plasma metabolome of beef replacement heifers. We hypothesized that 1) seaweed supplementation induces dose-dependent metabolic shifts in plasma, 2) methane emission groups are associated with unique metabolic signatures, and 3) the metabolic response to seaweed differs across methane emission groups, highlighting an interaction and enabling the identification of candidate biomarkers and biological pathways linked to methane mitigation.

## Materials and methods

### Diet preparation, adaptation, and feed sampling

All experimental procedures (Animal Use Protocol #2022-032) were approved by the Animal Care and Use Committee, Dalhousie University, Nova Scotia, Canada, and the animals were cared for in accordance with the guidelines of the Canadian Council on Animal Care (CCAC, 2009). The full processes of diet preparation and sampling, experimental design, and adaptation procedure were detailed in our previous study (Borzouie et al., 2026). In short, the feed trial included 20 pregnant replacement heifers (18 months of age) for 50 days. Pregnant replacement heifers were included since they represent an important forage-fed production stage in beef systems and also due to logistical and resource constraints of the project. The heifers were a mix of four breeds, including Black Angus, Red Angus, Hereford, and Shorthorn, and were randomly assigned to one of four treatment groups based on seaweed supplementation levels (0.0%, 0.5%, 1.0%, and 2.0% on a dry matter basis). Each group, comprising five heifers, was housed together, and their individual feed consumption and feeding behavior were recorded individually using the Vytelle System (Borzouie et al., 2026). Throughout the study, all heifers remained clinically healthy, and no signs of illness, abnormal behavior, or adverse responses to seaweed supplementation were observed. Animal performance monitoring during the 50-day feeding trial followed the procedures described by Borzouie et al. (2026). Briefly, individual feed intake was recorded daily using the Vytelle system, and body weight was measured at the start of the trial, at bi-weekly intervals, and at the end of the performance test. Performance outcomes, including average daily gain, dry matter intake, feed conversion ratio, body weight gain, and final body weight, were not significantly affected by seaweed supplementation, and no treatment-related adverse health observations were reported.

Feed items were analyzed at the beginning of the experiment for nutrient content (Borzouie et al., 2026). The nutrient composition of seaweed and the basal TMR fed during the two-week GHG measurement period is presented in Table 1. A two-week dietary adaptation period was established for the heifers to adapt to the treatment diets. The primary feed was prepared at the feed mill located at the Nappan Research Farm. During the two-week dietary adaptation period, seaweed meal was incorporated using a top-dressing method. After the basal feed was delivered into the bunk by the feed cart, the required daily amount of seaweed for each treatment group was weighed according to the target inclusion level and manually mixed into the assigned feed. Following the adaptation period, seaweed was added during feed preparation using a serial addition method to improve mixing consistency and distribution across the diet. In this method, the calculated amount of seaweed meal was first mixed with a small portion of the basal diet in the feed tank to create a preliminary blend, followed by stepwise addition and mixing of the remaining basal diet until the full batch was prepared. This approach promoted homogeneous seaweed distribution while maintaining the four target inclusion levels on a dry matter basis. The daily dry matter content of feed samples was measured before seaweed addition, and weekly averages were used to maintain the target inclusion levels.

**TABLE 1:** Nutrient composition of seaweed supplement and the dietary feed composition over 7 weeks of the performance trial (mean ± standard error).

Parameter	Seaweed supplement		Basal TMR^1^, GHG^2^ trial weeks 1–2	
	As fed	Dry	As fed	Dry
Dry matter (%)	86.7 ± 0.51	-	95.72	98.60
Crude protein (%)	11.99 ± 2.23	13.85 ± 2.65	11.75	12.02
Acid detergent fiber (%)	12.22 ± 1.02	14.09 ± 1.26	33.99	35.36
Calcium (%)	0.636 ± 0.06	0.733 ± 0.08	0.27	0.31
Potassium (%)	2.582 ± 0.70	2.972 ± 0.79	1.31	1.26
Magnesium (%)	0.615 ± 0.04	0.709 ± 0.04	0.15	0.14
Phosphorus (%)	0.092 ± 0.001	0.105 ± 0.001	0.29	0.29
Sodium (%)	1.511 ± 0.53	1.739 ± 0.59	0.04	0.03
Copper (ppm^3^)	-	<5.00	5.19	<5.00
Manganese (ppm)	341.88 ± 77.63	393.53 ± 87.17	58.93	49.74
Zinc (ppm)	32.32 ± 4.65	37.28 ± 5.58	24.59	23.08

1 TMR: Total mixed ration.

2 GHG: Greenhouse gas.

3 PPM: parts per million.

### GHG emissions and gas flux measurement

Methane emission monitoring methodology was previously described in Borzouie et al. (2026). Briefly, after completing the feeding trial, 16 of the 20 heifers were randomly selected and fed *ad libitum* the same diet that they had received during the feed test period for an additional 4-week GHG measurement trial. Daily diet samples were collected to adjust seaweed supplementation based on dry matter content. The 4-week GHG measurement trial consisted of a 2-week habituation period followed by a 2-week measurement period. Emissions were measured using four ventilated head-box metabolic chambers connected to a gas monitoring system (Qubit Systems Inc., Ontario, Canada). The system sequentially sampled gases from each chamber every 30 min over 24-h periods, and CO_2_, O_2_, and CH_4_ levels were recorded for each head-box. Gas leakage from the head-box chambers was minimized by maintaining continuous negative-pressure ventilation, with external vacuum pumps pulling air through each chamber at approximately 600 L/min. Adequate oxygen supply was ensured by this continuous airflow, and CO_2_ levels were remotely monitored with an alert system to notify staff if concentrations exceeded 8000 ppm. Habituation to the respiration chambers occurred over a 2-week period, progressing from short sessions to 24-h continuous exposure, following a structured schedule. During the subsequent 2-week data collection period, each heifer underwent two 24-h measurements of gas exchange to quantify individual CO_2_ exchange, O_2_ consumption, and CH_4_ emissions, following the established protocol and manufacturer recommendations (Borzouie et al., 2026). Of the 16 heifers enrolled in the measurement trial, one heifer was not tested after calf loss, and one measurement was incomplete due to head withdrawal from the head-box.

### Plasma sampling and sample preparation

Plasma samples were collected at the end of Week 1 and at the end of Week 2, during the 2-week GHG emission measurement period. The samples were centrifuged, and the resulting plasma was aliquoted and stored at −80 °C until shipment to the Metabolomics Innovation Center (TMIC), Edmonton, Alberta, for metabolomic analysis. At TMIC, samples were processed according to an established procedure: they were thawed on ice, vortexed for 15 s, and centrifuged at 13,000 × g for 10 min.

Two analytical platforms were used: liquid chromatography–tandem mass spectrometry (LC–MS/MS) and direct flow injection–tandem mass spectrometry (DFI–MS/MS). For LC–MS/MS, two derivatization chemistries were applied: phenylisothiocyanate (PITC) for polar metabolites (amino acids, biogenic amines, nucleosides) and 3-nitrophenylhydrazine (3-NPH) for organic acids. Following derivatization, samples were extracted and analyzed. Lipids and acylcarnitines were quantified without derivatization by DFI–MS/MS.

### Exploratory metabolomic analysis using a combination of LC–MS/MS and DFI–MS/MS analytical methods

A comprehensive targeted quantitative metabolomics technique using DFI/LC–MS/MS was employed with 40 µL of plasma. The technique involved chemical derivatization of selected metabolites, including organic acids, amino acids, amino acid derivatives, and biogenic amines, followed by analyte extraction. Lipids and acylcarnitines were separated using liquid chromatography or direct flow injection (DFI) analysis. Metabolites were then identified and quantified by selective mass spectrometric detection using multiple reaction monitoring (MRM) pairs. Detailed MRM transitions and acquisition parameters for the targeted metabolites are provided in Supplementary Table S1. This information follows the targeted LC–MS/MS serum and plasma metabolomics assay described by Zhang et al. (2024), where additional details on assay design, metabolite coverage, analytical conditions, transition selection, and validation are provided.

Accurate metabolite quantification was ensured using isotope-labeled internal standards and isotope-labeled derivatization reagents. Calibration standards were created by diluting stock solutions, covering a broad range of analyte concentrations. Three different quality control standards were prepared by diluting the highest calibration standard for amino acids, biogenic amines, nucleotides/nucleosides, and organic acids. For lipid analysis, chloroform (Sigma-Aldrich, Oakville, ON, CA) was the chosen solvent for stock solution preparation, while methanol was used for acylcarnitines and hexose. Stock solutions for organic acids were prepared in 75% LC–MS/MS-grade methanol mixed with LC–MS/MS-grade water.

Mass spectrometry was performed using an AB SCIEX 5500 QTrap coupled to an Agilent 1290 UHPLC system with a Zorbax Eclipse XDB C18 column. Metabolites were separated using a mobile phase of (A) 0.2% formic acid in water and (B) 0.2% formic acid in acetonitrile. The gradient was held at 0.0% B (0.0–0.5 min), increased to 95% B (5.5–6.5 min), then returned to 0.0% B (7.0 min) and held until 9.5 min. The flow rate was 500 μL/min, the injection volume was 10 μL, and the column temperature was 50 °C. The mass spectrometer operated in positive electrospray ionization mode with scheduled MRM.

For DFI–MS/MS, the autosampler was connected directly to the MS source. Organic acids were separated using solvents I (0.01% formic acid in water) and II (0.01% formic acid in acetonitrile) with a gradient: 25% II (0.0 min), 65% II (6 min), 90% II (6.3 min), 100% II (6.5 min), returning to 25% by 7.5 min, held to 12 min; flow 400 μL/min, injection 10 μL, column 40 °C. The MS was operated in negative MRM mode under the following conditions: IonSpray voltage, −4500 V; source temperature, 400 °C; CUR, 20; GAS1, 30; GAS2, 30; CAD, medium; and EP −10 V.

### Data processing and statistical analysis

Overall, the analytical workflow consisted of two components: (1) a primary GLM-based framework in R used to adjust metabolite and methane data and test the effects of seaweed treatment, methane emission category, and their interaction; and (2) complementary exploratory analyses in MetaboAnalyst used to identify discriminating metabolites, evaluate biomarker potential, and explore enriched biological pathways.

### First component, GLM framework

Since plasma metabolite concentrations are influenced by multiple confounders, and methane emissions similarly require adjustment for relevant covariates to obtain biologically meaningful values, data were processed and statistically analyzed in three steps. Step I) adjustment of metabolite concentrations for significant (p < 0.05) fixed and covariate effects; Step II) adjustment of methane emissions for significant (p < 0.05) fixed factors and covariates, and animals were subsequently ranked and categorized into three distinct groups based on their adjusted methane emission values to facilitate biological interpretation; and Step III) evaluation of the effects of seaweed treatment levels and methane emission category, and their interaction on adjusted plasma metabolite concentrations. Each of these three steps is described below.

#### Step I) metabolite profiling and adjustment of metabolite concentrations

Quantitative analysis of plasma metabolites was performed using the proprietary Analyst software version 1.6.3 (Sciex, Framingham, MA, USA) to facilitate peak integration, calibration against external standard curves, and calculation of absolute concentrations for each target metabolite. To ensure the robustness of subsequent statistical analyses and isolate biological effects from confounding technical and physiological variables, a normalization procedure was implemented using sum normalization with no transformation, followed by auto-scaling.

Metabolite concentrations were statistically adjusted using a Generalized Linear Model (GLM) in R (version 4.0.3) for significant (p < 0.05) fixed effects and covariates. The model included breed and feeding time as fixed effects, with age and initial body weight fitted as covariates, as described by the following equation.

$$Y_{\text{ijk}}=\mu +B_{i}+{\text{Time}}_{j}+\beta_{1}\left(A\right)+\beta_{2}\left(\text{BW}\right)+e_{\text{ijk}}$$

where $Y_{\text{ijk}}$ represents the non-adjusted metabolite concentrations measured in plasma, μ is the overall mean, B_i_ is the fixed effect of breed with three levels, Time_j_ is the fixed effect of time of feeding with five levels, $\beta_{1}\left(A\right)$ is the covariate of age measured (days), $\beta_{2}\left(\text{BW}\right)$ is the covariate of body weight, and $e_{ijk}$ is the random residual error. These adjusted metabolites were used as the basis for all subsequent analyses, allowing evaluation of seaweed treatment and methane emission effects independent of these factors.

#### Step II) adjustment and classification of methane emissions

The primary objective of this analysis was to rank animals by their adjusted methane emission levels and then group them into three categories (low, moderate, high) in order to link these categories to plasma metabolomic profiles. To achieve this, daily methane emissions (measured in grams per day) were first analyzed using an analysis of variance (ANOVA) that included fixed effects for seaweed supplementation level, breed, and feeding time, and covariates such as body weight, age, and DMI. This ANOVA served to identify which factors had a statistically significant overall effect on methane emissions. Based on the ANOVA results, a final GLM was fitted in R as follows.

$$Y_{ij}=\mu +T_{i}+\varepsilon_{ij}$$

In this equation, $Y_{ij}$ represents the observed methane emissions for each animal, μ is the overall mean, and $\varepsilon_{ij}$ is the random residual error. The Ti estimated the effect of seaweed supplementation on methane emissions. Other fixed effects, including breed and feeding time, as well as covariates such as body weight, age, and DMI, were initially included but were not significant (p > 0.05) and were therefore removed. The final model retained seaweed supplementation as the only significant predictor (p < 0.05).

Adjusted methane emission values derived from this final model were then used to rank animals and classify them into three methane emission groups. Animals with adjusted methane emission values more than one standard deviation below the mean were classified as low emitters, those more than one standard deviation above the mean as high emitters, and those within one standard deviation of the mean as moderate emitters. These groups were subsequently used to examine associations with plasma metabolomic profiles.

#### Step III) integrated analysis of seaweed treatment and methane emission category on plasma metabolite concentrations

The final analytical stage of GLM analysis focused on exploring the relationships between plasma metabolite concentrations, seaweed supplementation, and methane emission categories. Specifically, we tested whether metabolite concentrations significantly differed according to methane emission category (low, moderate, high), seaweed supplementation level (0.0%, 0.5%, 1.0%, and 2.0%), and the interaction between these two factors.

Using the adjusted metabolite concentrations prepared under Step I, statistical analyses were performed using a GLM in R, incorporating both main effects and interaction terms. The regression model was specified as follows:

$$Y_{ijk}=\mu +T_{i}+E_{j}+{\left(T\times E\right)}_{ij}+\varepsilon_{ijk}$$

In this model, $Y_{ijk}$ represents an adjusted plasma metabolite concentration for an animal *k* receiving seaweed treatment i and classified into methane emission category j , μ is the overall mean, $T_{i}$ is the fixed effect of seaweed supplementation (0.0%, 0.5%, 1.0%, 2.0%), $E_{j}$ is the fixed effect of methane emission group (low, moderate, high), ${\left(T\times E\right)}_{ij}$ is the interaction between treatment and emission group, and $\varepsilon_{ijk}$ is the random residual error. This model was used to evaluate the effects of seaweed supplementation, methane emission category, and their interaction on plasma metabolite concentrations.

### Complementary multivariate, biomarker discovery, and pathway analyses

Following the primary GLM-based analyses described above, which tested each metabolite individually for associations with seaweed dose, methane group, or their interaction, complementary multivariate, biomarker discovery, and pathway analyses were conducted in MetaboAnalyst 6.0. These supportive exploratory approaches examined how the overall metabolite profiles could separate groups. These analyses were used to further identify discriminating metabolites, assess multivariate separation among comparison groups, evaluate the biomarker potential of selected metabolites, and determine enriched biological pathways associated with seaweed supplementation and methane emission groups. Thus, these results were intended to complement the main inferential results obtained from the GLM framework. Both univariate and multivariate analyses were applied to the LC–MS/MS metabolomic data. Metabolite concentrations were uploaded to the MetaboAnalyst 6.0 platform following established protocols (Xia et al., 2009; Xia and Wishart, 2010; Pang et al., 2024).

Prior to statistical analysis, the metabolomic dataset was processed using standard procedures in MetaboAnalyst 6.0. From the 453 identified metabolites, 34 metabolites with >20% missing values were excluded from the analysis. The remaining data were normalized using Probability Quotient Normalization (PQN), with the control group used as the reference. Univariate statistical methods included fold change calculation, Student’s t-tests, and volcano plot generation. Pairwise t-tests were performed two-by-two between seaweed groups (0.0%, 0.5%, 1.0%, and 2.0%) and methane categories (high, moderate, low). Multivariate analysis was performed using partial least squares discriminant analysis (PLS-DA) to assess variation and separate experimental groups. The significance of each PLS-DA model was verified through permutation testing (n = 1000, with significance declared at p < 0.05). For models that reached significance (p < 0.05), the 15 metabolites with the highest Variable Importance in Projection (VIP) scores were examined. VIP scores quantify each metabolite’s contribution to the PLS-DA model, allowing ranking of features driving group discrimination, with metabolites showing VIP >1 considered the most influential in distinguishing between comparison groups. A Random Forest classification analysis (500 trees and seven predictors, with random feature selection) was additionally applied as a complementary non-linear method to validate the discriminant features identified by PLS-DA. Feature importance was assessed using Mean Decrease Accuracy (MDA), which quantifies the reduction in classification accuracy when a given metabolite is permuted, thereby providing a robust ranking of predictive importance. Metabolites with positive MDA values were interpreted as contributing positively to classification accuracy.

Candidate biomarkers consisting of significant metabolites (p < 0.05) identified from both univariate and multivariate analyses were manually selected and further evaluated using the MetaboAnalyst six biomarker analysis module. Potential biomarkers were evaluated using receiver operating characteristic (ROC) analysis combined with logistic regression and 10-fold cross-validation on normalized and scaled metabolite subsets. ROC analysis was used to determine the diagnostic performance of each metabolite by quantifying sensitivity and specificity across varying thresholds. Selection was based on two primary criteria including the Area Under the Curve (AUC) ≥ 0.75 for discriminative power, between 0.65 and 0.75 as a trend or tendency, and statistical significance (FDR-corrected p < 0.05) from univariate analysis. Metabolites that, individually or in combination, yielded robust predictive models (AUC ≥0.75) with significant PLS-DA-based accuracy were considered important potential biomarkers. This methodology follows the biomarker discovery framework established by Xia et al. (2013).

To provide biological context, enrichment and pathway analyses were conducted using MetaboAnalyst 6.0 and KEGG-linked pathway mapping, where available, to link the identified key candidate metabolites to known biological pathways and mechanisms. The targeted DFI/LC–MS/MS panel quantified 453 plasma metabolites across multiple biochemical classes, including amino acids, amino acid derivatives, biogenic amines, nucleotides/nucleosides, organic acids, acylcarnitines, triglycerides, phosphatidylcholines, lysophosphatidylcholines, sphingomyelins, ceramides, and cholesterol esters. After exclusion of metabolites with >20% missing values, 419 metabolites were retained for downstream analysis. Pathway analysis of the mapped metabolites identified 16 KEGG-linked pathways. Pathways were prioritized if they met both criteria of p < 0.05 and a pathway impact score >0.2. Because the dataset was generated using a targeted metabolomics panel, pathway enrichment results were interpreted relative to the metabolites measured and successfully mapped to pathway databases, rather than as an unbiased representation of the complete metabolome. Therefore, enriched pathways were used to support biological interpretation of the significant metabolites and not as standalone evidence of global pathway activity. Unless otherwise stated, the threshold for statistical significance in this study was p < 0.05 and a Benjamini–Hochberg false discovery rate (Q) < 0.05. A tendency was defined as 0.05 ≤ p < 0.10.

## Results

The findings are presented in three sections. The first section describes changes in plasma metabolite levels associated with dietary seaweed supplementation in replacement heifers. The second section focuses on key discriminating metabolites specifically associated with enteric methane emission groups. The third section examines the interaction between seaweed supplementation and methane emission groups. Concise summary tables are presented in the main text to highlight the key candidate metabolites, whereas the full method-specific metabolite lists are provided in the Supplementary Material.

### Independent effects of seaweed supplementation on plasma metabolomic profiles of replacement heifers

Table 2 summarizes the highest-confidence plasma metabolites associated with seaweed supplementation, defined as metabolites consistently identified across multiple statistical approaches. Full method-specific metabolite lists from t-test, volcano plot, ROC, PLS-DA, and Random Forest analyses across all pairwise seaweed comparisons are provided in Supplementary Table S2. Overall, lipid-related metabolites and amino acid derivatives were the main metabolite classes altered by seaweed inclusion (Table 2; Supplementary Table S2). The 0.0% vs. 1.0% comparison showed that triglycerides (e.g., TG.18:0_30:1, TG.18:0_36:4) and phosphatidylcholines (PC ae C40.4 and PC aa C36.6) were the strongest discriminators (AUC >0.75, p < 0.05), suggesting lipid metabolism shifts at moderate doses (Supplementary Table S2). In contrast, the 0.0% vs. 2.0% comparison demonstrated the most pronounced differentiation, with metabolites linked to energy metabolism (valine, inosine, C2 acylcarnitine, oxoisocaproic acid) and nitrogen balance (ADMA and TMA) being prominent. Trimethylamine, a metabolite commonly linked to gut microbial metabolism, was elevated in the 2.0% group across ROC, PLS-DA, and volcano plot analyses, suggesting a possible association between higher seaweed inclusion and host-microbial co-metabolism. The univariate results (t-test and volcano plot) showed that intermediate dose comparisons (0.0% vs. 0.5%, 0.0% vs. 1.0%, and 0.5% vs. 1.0%) revealed fewer significant metabolites compared to the high-dose comparisons (0.0% vs. 2.0%, 0.5% vs. 2.0%, and 1.0% vs. 2.0%), suggesting a non-linear metabolic response (Supplementary Table S2). Specifically, the 1.0% vs. 2.0% comparison showed significant changes in amino acid-related metabolites (arginic acid and proline; p < 0.05), indicating a metabolic shift as supplementation increased from 1.0% to 2.0%. In the 0.5% vs. 2.0% comparison, the univariate t-test identified cytidine as the only significant metabolite (p = 0.02), implying modulation of nucleotide metabolism at higher doses. Notably, no significant metabolites were identified by univariate analyses in the 0.0% vs. 0.5%, 0.0% vs. 1.0%, or 0.5% vs. 1.0% comparisons, underscoring minimal metabolic disruption at lower inclusion levels.

**TABLE 2:** Highest-confidence plasma metabolites associated with seaweed supplementation in replacement heifers.

Pairwise comparison	Highest-confidence metabolites^1^	Evidence in manuscript	Interpretive note
0.0% vs. 2.0%	Valine; TG.18:0_38:6; ADMA; C2 acylcarnitine; trimethylamine; inosine	Significant across all 5 methods	Strongest dose response; reflects lipid, amino acid, microbial-related, and energy metabolism
1.0% vs. 2.0%	Argininic acid; proline; TG.18:2_32:1; TG.18:1_26:0; TG.16:1_36:1; allantoin	Detected in ≥3 methods	Supports metabolic shift between moderate and highest seaweed inclusion
0.5% vs. 2.0%	Cytidine; deoxycytidine; dimethylamine; selected TGs	Detected across multiple methods, but fewer robust markers than 0.0% vs. 2.0%	Suggests nucleotide- and lipid-related responses at higher dose
0.0% vs. 1.0%	TG.18:0_30:1; PC ae C40.4; PC aa C36.6; selected TGs	Mainly multivariate support; no univariate significance	Consistent with early lipid-metabolism shifts at 1.0% inclusion
0.0% vs. 0.5% and 0.5% vs. 1.0%	No high-confidence markers	Minimal or no univariate significance	Suggests limited metabolic perturbation at lower inclusion levels

1 Metabolite abbreviations: TG, triacylglycerol; ADMA, asymmetric dimethylarginine; C2, acetylcarnitine; PC, phosphatidylcholine; ae, acyl-alkyl; aa, diacyl.

This table preserves the main conclusion that the strongest metabolomic shifts occurred at the 2.0% inclusion level and that lower inclusion levels caused minimal disruption. Full method-specific metabolite lists are presented in Supplementary Table S2.

In contrast, intermediate and low-dose contrasts (0.0% vs. 0.5% and 0.0% vs. 1.0%) yielded fewer high-confidence markers. The 0.5% vs. 2.0% comparison highlighted nucleotide metabolites (cytidine and deoxycytidine) as top hits, and the 0.5% vs. 1.0% contrast produced four consistent markers (cytidine, deoxycytidine, methionine sulfoxide, N-acetylglutamine). Lower-dose comparisons (0.0% vs. 0.5% and 0.0% vs. 1.0%) lacked any metabolites validated by ≥ 3 methods, indicating minimal metabolic perturbation. However, select lipids (e.g., TG.18:0_30:1) and neuroactive compounds (serotonin) reached significance in two methods at the 1.0% level, suggesting subtle, early metabolic shifts (Supplementary Table S2).

Across all five statistical approaches (t-test, volcano plot, ROC AUC, PLS-DA VIP, and Random Forest MDA), metabolites identified by three or more statistical methods were prioritized as the strongest biomarkers. The 0.0% vs. 2.0% comparison stood out: six metabolites (valine, TG.18:0_38:6, asymmetric dimethylarginine, C2, trimethylamine, inosine) were significant across all five analyses, underscoring a robust metabolic shift at the highest dose. The 1.0% vs. 2.0% comparison also revealed a clear dose effect, with six core discriminators (argininic acid, proline, TG.18:2_32:1, TG.18:1_26:0, TG.16:1_36:1, allantoin) appearing in at least three methods, plus several additional triglycerides in two methods (Supplementary Table S2).

These results collectively demonstrate dose-specific alterations in the plasma metabolome with lipid and amino acid pathways as major contributors to group discrimination. The most consistently identified potential candidate biomarkers of seaweed supplementation across pairwise comparisons included trimethylamine, asymmetric dimethylarginine, valine, inosine, C2, TG.18:0_38:6, cytidine, proline, and argininic acid. TG.18:0_30:1 appeared to be a potential early indicator of the metabolic response to seaweed supplementation at the 1.0% inclusion level.

### Metabolic pathways altered by seaweed supplementation

Within the measured targeted metabolite panel, pathway and enrichment analyses revealed two pathways significantly associated with seaweed supplementation, including pyrimidine metabolism (p = 0.006) and valine, leucine, and isoleucine biosynthesis (p = 0.026), both with a pathway impact score of 1.0. These findings indicate that seaweed supplementation may alter nucleotide metabolism and branched-chain amino acid biosynthesis, suggesting potential effects on nucleic acid turnover, protein homeostasis, and energy metabolism in replacement heifers. Pathway annotation of the candidate metabolites is provided in the Supplementary Table S8, which links key metabolites to their corresponding enriched pathways.

### Independent effects of methane emission groups on plasma metabolomic profiles of replacement heifers

The results in Supplementary Table S3 show the classification of animals into low, moderate, and high methane emission groups based on methane mean values adjusted for other influencing factors. This classification provided the foundation for subsequent metabolomic comparisons across the three emission levels. Distinct plasma metabolite profiles were identified across methane emission groups in replacement heifers through multivariate (ROC, PLS-DA, and Random Forest) and univariate analyses (t-test and volcano plot). Table 3 provides a concise summary of the key metabolites associated with methane emission groups, while Supplementary Table S4 presents the complete method-specific lists of methane-group metabolites identified across the pairwise comparisons.

**TABLE 3:** Highest-confidence plasma metabolites associated with methane-emission group.

Methane-group comparison	Highest-confidence metabolites^1^	Evidence in manuscript	Interpretive note
High vs. Moderate	Caproic acid; propionic acid; urea; Hex2Cer.d18.1.16.0; multiple TG species	Most extensive separation; supported by univariate and multivariate analyses	Indicates strong differences in microbial fermentation, lipid metabolism, and nitrogen handling
Low vs. Moderate	TG.18:3_36:4; HexCer.d18.1.24.1; α-ketoisovaleric acid; PC ae C40.5; PC ae C38.6; multiple TGs	Robust discrimination across methods	Highlights lipid- and branched-chain-amino-acid-related differences
High vs. Low	Urea; ethylmalonic acid; PC aa C32.2	Mainly multivariate support; fewer univariate hits	Suggests weaker but detectable differences between extreme emitters

1 Metabolite abbreviations: TG, triacylglycerol; Hex2Cer, dihexosylceramide; HexCer, hexosylceramide; PC, phosphatidylcholine; ae, acyl-alkyl; aa, diacyl; α, alpha.

The full methane-classification table is presented in Supplementary Table S3, and the longer method-specific methane-group metabolite lists are presented in Supplementary Table S4.

The High vs. Moderate group comparison revealed the most extensive metabolite shifts, with 14 potential biomarkers identified using five complementary analytical approaches (ROC analysis, AUC >0.75; PLS-DA, VIP score >1; t-test, p < 0.05; Random Forest, MDA >1; and volcano plot, p < 0.05) (Table 3; Supplementary Table S5). Short-chain fatty acids (caproic and propionic acids) and urea were prominent alongside dihexosylceramides (Hex2Cer.d18.1.16.0), multiple triglyceride species (notably C18:2-enriched forms), and microbial-derived metabolites such as propionic acid, caproic acid, and ethylmalonic acid.

For the High vs. Low comparison, the most robust discriminators included urea and select phosphatidylcholines, with PC aa C32.2 emerging as a consistent differentiating metabolite across PLS-DA and Random Forest analyses (VIP >1, MDA >1). Organic acids (e.g., ethylmalonic acid) also contributed to group separation, though univariate tests did not yield significant results for this comparison.

In the Low vs. Moderate comparison, lipid species dominated the discriminant profile. Triglycerides with medium-to-long-chain fatty acids (C34-C40) were consistently elevated across all five statistical methods mentioned above, alongside sphingolipids (e.g., hexosylceramide HexCer.d18.1.24.1) and branched-chain amino acid derivatives (α-ketoisovaleric acid). Phosphatidylcholines, particularly plasmalogen species (PC ae C36.5–C40.5), further distinguished these groups.

Metabolite differences between methane emission groups, summarized in Table 3 with full method-specific details provided in Supplementary Table S4, collectively include changes in lipid-related metabolites (triglycerides and phosphatidylcholines) and nitrogen-related metabolites (urea, N1-acetyllysine/N6-acetyllysine, and branched-chain amino acids) across the compared groups Notably, short-chain fatty acids (propionic acid) and medium-chain fatty acids (caproic acid), which are commonly associated with microbial fermentation, were enriched in moderate and high emission groups, suggesting a possible association among microbial-related metabolites, lipid metabolism, and methane production. Overall, these patterns show that the metabolic changes are more pronounced between moderate and high emitters than between the extreme emission groups. Triglycerides with carbon numbers in the C28–C36 range consistently differentiated high emitters, while urea recurred as a cross-comparison metabolite. As Supplementary Table S4 shows, metabolites related to lipid metabolism, urea cycling, and fermentation-associated pathways differed across the methane emission gradient.

### Metabolic pathways altered by methane emission groups

Within the annotated targeted metabolite panel, pathway analysis linked methane emission groups to differences in amino acid, CoA-related, arginine, and propanoate metabolism. Comparisons between low- and moderate-methane-emitting groups identified significant enrichment in valine, leucine, and isoleucine biosynthesis and degradation (p = 0.02), and pantothenate and CoA biosynthesis (p = 0.01). Conversely, comparisons between high- and moderate-methane-emitting groups demonstrated significant enrichment in arginine biosynthesis (p = 0.02), and propanoate metabolism (p = 0.03). No significant pathways were detected between the high versus low methane emitting groups. This pattern suggests that the moderate methane-emission group may be metabolically distinct from both extreme groups at the pathway level.

### Key metabolites distinguishing the overall effects of seaweed Supplementation and methane emission category in replacement heifers

**FIGURE 1:**
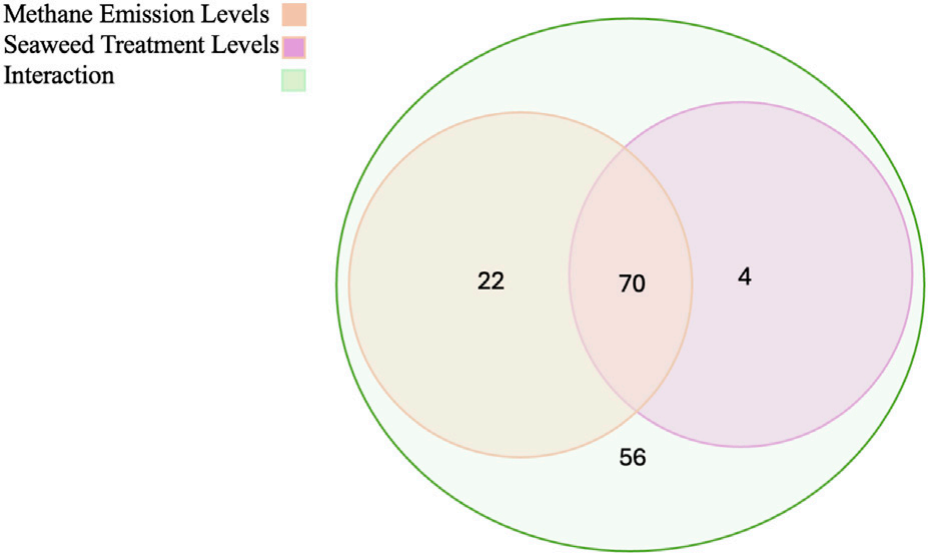
Number of significant plasma metabolites identified by two-way ANOVA associated with methane emission grouping, seaweed supplementation level, and their interaction effects in replacement heifers.

A total of 453 plasma metabolites (Supplementary Table S5) concentrations (mmol/L) in replacement heifers supplemented with four levels of Atlantic GRO® brown seaweed and categorized into three methane emission groups were subjected to analysis, and the results are presented in Supplementary Table S3. The FDR-adjusted p-values from the ANOVA indicated that 152 metabolites were significantly impacted (p < 0.05) by the interaction between seaweed supplementation and methane emission groups (Supplementary Table S7), highlighting the complex metabolic interplay between dietary seaweed and methane emissions. Among these, 70 metabolites were common to both experimental conditions, including seaweed supplementation and methane emission group (Supplementary Table S6). These results are visually summarized in Figure 1, which illustrates the overlapping and unique metabolite counts.

Following the ANOVA, metabolomic changes were further analyzed using Random Forest analysis, which ranked metabolites by their discriminatory power (measured via MDA after random permutation). Table 4 provides a concise summary of the key discriminating metabolites identified from the interaction analyses, whereas Supplementary Table S7 presents the complete interaction results, including the full set of metabolites identified across the method-specific analyses. As shown in Supplementary Table S7, the top 15 candidate metabolites associated with the interaction between the two factors included glycerophospholipids and phospholipids, organic acids and derivatives (e.g., ethylmalonic acid, hydroxyphenylpyruvic acid, 4-hydroxyproline, and cis-aconitic acid), and benzenoids (e.g., hydroxyphenylpyruvic acid). Eight significant discriminating metabolites, including PC aa C40.6, PC aa C38.6, PC ae C38.5, TG.18:1_38:5, creatinine, PC ae C38.0, CE.14.1, and PC ae C40.5, were consistently detected through both two-way ANOVA and Random Forest analysis (p < 0.05, MDA >0.001).

**TABLE 4:** Key discriminating metabolites associated with the interaction between seaweed supplementation and methane-emission group.

Analysis level	Key metabolites^1^	Evidence in manuscript	Interpretive note
Two-way ANOVA^2^ + random forest overlap	PC aa C40.6; PC ae C38.5; PC ae C40.5; TG.18:1_38:5; creatinine; CE.14:1	Significant in both ANOVA and random forest	Represents robust discriminators of the seaweed × methane interaction
ROC^3^-discriminating metabolites	TG.16:0_38:2; TG.16:0_35:3; TG.20:1_34:0; TG.18:2_28:0; LysoPC a C18:2; urea; oxalic acid	AUC ≥0.75 with significant discrimination	Highlights lipid-, nitrogen-, and energy-related metabolites
Most informative pairwise interaction contrast	Sarcosine; threonine; α-ketoglutaric acid; fumaric acid; cystathionine; α-ketoisovaleric acid; propionic acid; LysoPC a C18:2	0.5% vs. 2.0% across methane groups showed the clearest pathway enrichment	Supports a dose-dependent metabolic transition involving amino acid, TCA-cycle, and microbial pathways

1 Metabolite abbreviations: PC, phosphatidylcholine; aa, diacyl; ae, acyl-alkyl; TG, triacylglycerol; CE, cholesteryl ester; LysoPC, lysophosphatidylcholine; AUC, area under the curve; TCA, tricarboxylic acid; α, alpha.

2 ANOVA, analysis of variance.

3 ROC, receiver operating characteristic.

This summary table preserves the manuscript’s conclusion that 152 metabolites were significantly affected by the interaction. Only the most informative biomarkers are presented in the main text, and the full metabolite lists are presented in Supplementary Table S7.

Furthermore, we used ROC analysis to identify discriminatory metabolites (AUC ≥0.75, p < 0.05) associated with the interaction between seaweed supplementation and methane emission groups. A total of 15 metabolites were identified (Table 4; Supplementary Table S7), including triglycerides (TGs), amino acids, and organic acids. Among these, six metabolites, TG.16:0_38:2, TG.16:0_35:3, TG.20:1_34:0, TG.14:0_38:4, TG.18:2_28:0, and LysoPC a C18:2, were also highlighted in multivariate analyses as key discriminators of metabolic pathway shifts linked to the interactive effects of seaweed supplementation and methane emission groups.

The ROC analysis for the seaweed supplementation × methane emission group interaction (Supplementary Table S7) identified discriminating metabolites in the following pairwise seaweed comparisons: 0.0% vs. 1.0% across Low, High, and Moderate methane emission groups; 0.0% vs. 2.0% across High and Moderate groups; 0.5% vs. 1.0% across Low and Moderate groups; and 0.5% vs. 2.0% across Low, High, and Moderate groups. In contrast, no significant discriminating metabolites were detected for 0.0% vs. 0.5% across High and Moderate groups, or for 1.0% vs. 2.0% across Low, High, and Moderate groups. A single metabolite, homoarginine, discriminated between 0.5% and 1.0% seaweed inclusion levels (AUC = 0.91, p = 0.04). The contrast between 0.0% and 1.0% seaweed yielded four significant metabolites (AUC ≥0.75, p < 0.05): ethylmalonic acid, hippuric acid, PC ae C42.3, and TG.20:3_32:1. The contrast between 0.0% and 2.0% seaweed interacting with different methane groups resulted in numerous significant metabolites (Supplementary Table S7) with top discriminators including TG.18:0_32:2, TG.16:0_38:2, indole-3-propionic acid, and phenylacetylglutamine. The most informative contrast, 0.5% vs. 2.0% seaweed across methane emission groups, is summarized in Table 4, with the complete pairwise ROC metabolite lists provided in Supplementary Table S7. The comparison revealed 15 discriminating metabolites associated with lipid, amino acid, and energy pathways including sarcosine and threonine (one-carbon metabolism), α-ketoglutaric acid and fumaric acid (TCA cycle), trimethylamine N-oxide (TMAO), and hippuric acid. Overall, the ROC analyses supported a dose-dependent metabolomic response, with the strongest discriminatory profiles observed at higher seaweed inclusion levels.

### Metabolic pathways altered by the combined effects of seaweed supplementation and methane emission

Within the measured and pathway-mapped metabolites, enrichment analysis of significantly (p < 0.05) altered metabolites and potential biomarkers from Supplementary Table S7, revealed significant biological pathways associated with the interactive effects of dietary seaweed supplementation and methane emission groups on the plasma metabolome of replacement heifers. The pathway annotation of these key metabolites is summarized in Supplementary Table S8. Overall, significant enrichment was observed in arginine biosynthesis (p = 0.01), a pathway critical for nitric oxide production, growth, reproduction, and immune defense, as well as in purine metabolism (p = 0.04), which is fundamental to nucleotide synthesis and cellular energy transfer and nitrogen utilization efficiency, suggesting their importance in the host response to these combined factors.

Among the six pairwise comparisons between seaweed treatment levels within methane emission groups noted in Supplementary Table S7, only the 0.5% vs. 2.0% comparison across low, high, and moderate methane emission groups resulted in significant enrichment of multiple metabolic pathways. Enrichment and pathway analyses of significantly altered metabolites and candidate biomarkers (p < 0.05, AUC >0.75) in this comparison revealed ten significantly impacted pathways: valine, leucine, and isoleucine biosynthesis; glycine, serine, and threonine metabolism; citrate cycle (TCA cycle); alanine, aspartate, and glutamate metabolism; valine, leucine, and isoleucine degradation; arginine biosynthesis; pyruvate metabolism; lipoic acid metabolism; cysteine and methionine metabolism; and tyrosine metabolism. These pathways were not significantly enriched in any other pairwise seaweed group comparisons. Their specific alteration in the 0.5% vs. 2.0% group across methane categories indicates that other dosage contrasts induced insufficient metabolic change to alter this pathway set. Together, these results support a dose-dependent shift, with a clear metabolic change occurring between the 0.5% and 2.0% supplementation levels. To provide metabolite-level biological context, pathway annotation of the candidate metabolites associated with seaweed supplementation, methane emission phenotype, and their interaction is summarized in Supplementary Table S8, which links reported key candidate metabolites to their corresponding enriched pathways or pathway annotations.

## Discussion

This study evaluated the effects of seaweed supplementation and methane emission group on the plasma metabolome of replacement heifers. Overall, the results showed dose-dependent metabolomic shifts associated with Atlantic GRO® supplementation, stronger effects at 1.0% and 2.0% inclusion, and an interaction between seaweed supplementation and methane emission phenotype. These findings are consistent with previous reports that marine-derived supplements can influence energy, lipid, and amino acid metabolism in ruminants and may help clarify the mechanisms and broader potential effects of methane mitigation strategies.

The present study suggests that dietary seaweed supplementation induces dose-dependent alterations in the plasma metabolome of replacement heifers that extend beyond a simple linear dose-response relationship. Similar to previous work in other species, higher inclusion levels were associated with stronger shifts in lipid-related metabolites and amino acid derivatives. In our study, minimal metabolic change was observed at 0.5%, whereas clearer differences emerged at 1.0% and were most pronounced at 2.0%. This pattern is consistent with our earlier report that methane mitigation became significant only at inclusion levels ≥1.0% (Borzouie et al., 2026), suggesting that measurable metabolic responses may require a minimum threshold of bioactive exposure. At 1.0% inclusion, triglycerides such as TG.18:0_30:1 and phosphatidylcholines such as PC ae C40.4 appeared to act as early discriminating metabolites, whereas at 2.0% inclusion the strongest changes included valine, oxoisocaproic acid, trimethylamine, and C2 acylcarnitine, indicating broader alterations in amino acid, microbial-related, and energy metabolism.

Focusing on the independent effect of seaweed dose, no significant metabolites were identified in the 0.0% vs. 0.5%, 0.0% vs. 1.0%, or 0.5% vs. 1.0% comparisons by univariate analyses, supporting the view that lower inclusion levels induced limited metabolic disruption. In contrast, more substantial differences were observed for 0.0% vs. 2.0% and 1.0% vs. 2.0%. Collectively, these results indicate that seaweed intake was associated with dose-specific alterations in the plasma metabolome, with lipid- and amino acid-related metabolites emerging as major contributors to group discrimination. This dose-dependent pattern provides a metabolic context for the methane reduction threshold previously reported for this seaweed product. Given the significance of the main metabolites, the discussion section was grouped under lipid, amino acid, and energy metabolism.

### Lipid metabolism alterations

Interactions between seaweed supplementation and methane emission group were strongly associated with lipid metabolism, with triacylglycerols (TGs), phosphatidylcholines (PCs), and lysophosphatidylcholines (LysoPCs) identified as major discriminators in the interaction analysis (Supplementary Table S7). Among these, several TG species showed lower average levels in the 1.0% and 2.0% seaweed groups than in the higher-CH_4_ groups represented by 0.0% and 0.5% inclusion. The largest relative declines were observed for TG.20:1_34:2, TG.18:2_28:0, and TG.20:1_34:0, whereas TG.22:4_34:2 increased, suggesting selective modulation of fatty-acid composition rather than uniform TG suppression. These patterns may reflect altered lipid metabolism or energy partitioning associated with reduced methanogenesis.

One possible explanation is that methane suppression at higher seaweed inclusion was associated with changes in hydrogen disposal and fermentation-related metabolism, which may have contributed to the observed host lipid responses. This interpretation is consistent with studies showing that seaweed bioactive compounds, including bromoform in *Asparagopsis*, can inhibit methanogens and redirect H_2_ toward propionate synthesis, a glucogenic precursor that draws on reducing equivalents through several metabolic pathways (Li et al., 2024; Choi et al., 2022). In that context, increased propionate formation could reduce the availability of fatty acid building blocks required for certain saturated and monounsaturated TG species. The increase in TG.22:4_34:2, however, may reflect selective incorporation of fatty acids derived from seaweed.

This interpretation is also broadly consistent with earlier reports showing that dietary lipids can reduce methane production by inhibiting methanogenesis during rumen fermentation (Machmüller et al., 2000; Doreau and Ferlay, 1995). Our previous study showed that 1.0% and 2.0% seaweed inclusion reduced methane emissions (Borzouie et al., 2026), and the present metabolomic results suggest that lipid-related metabolites may serve as candidate biomarkers of that response. Similarly, previous studies have shown that dietary lipids, including medium-chain fatty acids and polyunsaturated oils, can modify ruminal fermentation and microbial populations while reducing methane production (Vargas et al., 2020; Rasmussen and Harrison, 2011; de Ondarza et al., 2024; Haque, 2018; Ahmad et al., 2025). Thus, the TG shifts observed here are consistent with the hypothesis that seaweed-associated methane mitigation is accompanied by altered host–microbe energy metabolism.

Another, not mutually exclusive, explanation is that seaweed bioactive compounds may have influenced lipid digestion, absorption, or mobilization. For example, TG.18:0_32:2, TG.16:0_38:2, and PC ae C42.3 were identified in the higher seaweed groups and co-occurred with metabolites commonly linked to host-microbial co-metabolism, such as indole-3-propionic acid and phenylacetylglutamine (Wang et al., 2021). Brown seaweeds contain polyphenols and carbohydrates such as laminarin, mannitol, alginic acid, and fucoidans that can be used by rumen microbes and may indirectly affect lipid metabolism through changes in digestion and fermentation (Maia et al., 2016). In addition, seaweed polyphenols may modulate rumen microbial activity and digestive processes, thereby influencing carbohydrate fermentation, protein degradation, and lipid utilization (Vasta et al., 2019). Because rumen microbial profiles were not measured in the present study, these interpretations should be viewed as plausible associations rather than direct evidence of microbiome-mediated mechanisms.

LysoPC a C18:2 also emerged as a notable candidate biomarker. Its concentration was markedly higher in the high-CH_4_ groups and lower in the 1.0% and 2.0% seaweed groups. Because LysoPCs are pro-inflammatory lipid mediators associated with oxidative stress and impaired lipid metabolism (Law et al., 2019), the reduced signal in the higher seaweed groups may indicate lower lipid peroxidation or improved oxidative stability. This interpretation is consistent with the reported antioxidant properties of brown seaweed polyphenols, particularly phlorotannins, which may contribute to improved oxidative stability and potentially influence rumen conditions (Choi et al., 2022; Bošnjaković et al., 2024).

Beyond lipid-specific metabolites, pathway enrichment analysis identified valine, leucine, and isoleucine biosynthesis and pyrimidine metabolism as pathways associated with seaweed supplementation. These findings suggest that seaweed-associated metabolic responses extend beyond lipid metabolism to include changes in amino acid and nucleotide metabolism. In dairy cows, rumen-protected branched-chain amino acid supplementation has been shown to increase valine, leucine, and isoleucine while reducing acylcarnitines, a pattern that is broadly consistent with the plasma shifts observed here (Xu et al., 2022). Together, these results support the view that higher seaweed inclusion is associated with coordinated changes in lipid, amino acid, and energy-related metabolism.

Overall, the metabolomic data support a dose-dependent response to brown seaweed supplementation. Minimal metabolic disruption was observed at 0.5%, whereas stronger discrimination occurred at 1.0% and was most pronounced at 2.0%. This pattern is consistent with our earlier finding that methane reduction became significant only at inclusion levels ≥1.0% (Borzouie et al., 2026). The enrichment of arginine biosynthesis in the 0.5% vs. 2.0% comparison, together with the appearance of homoarginine in the 0.5% vs. 1.0% contrast, suggests that arginine-related metabolism may be associated with the metabolic transition accompanying effective methane mitigation, although direct mechanistic conclusions cannot be drawn from the present data alone. A further targeted study tracing labelled arginine and homoarginine in rumen fluid could test whether homoarginine directly suppresses methanogenesis.

### Amino acid metabolism

Methane emission group and seaweed supplementation were both associated with changes in amino acid metabolism, with isoleucine, α-ketoisovaleric acid, two-oxoisocaproic acid, threonine, homoarginine, sarcosine, and cystathionine emerging as discriminating metabolites. In addition, metabolites such as hydroxyphenylpyruvic acid, 4-hydroxyproline, homoarginine, and sarcosine suggest possible links with amino acid metabolism, protein turnover, collagen synthesis, and oxidative stress responses (Wu et al., 2016).

Urea emerged as one of the most consistent metabolites associated with the seaweed × methane interaction. Concentrations were highest in the 0.0% group and generally decreased in the seaweed-supplemented groups, with the lowest values observed at 2.0% inclusion. Elevated urea in the 0.0% and 0.5% groups may reflect less efficient nitrogen utilization and greater rumen ammonia loss, which is consistent with their higher methane emissions (Matthews et al., 2019). This interpretation is also aligned with our previous findings that 1.0% and 2.0% seaweed inclusion were associated with the lowest CH_4_ emissions (Borzouie et al., 2026). One possible explanation is that seaweed supplementation was associated with changes in nitrogen-related metabolism that may reflect improved nitrogen retention or microbial protein synthesis. This interpretation is supported by reports that seaweed bioactive compounds, including phlorotannins, may inhibit urease-producing bacteria and enhance nitrogen efficiency (Orzuna-Orzuna et al., 2024).

A broader metabolic shift was observed at the 2.0% seaweed inclusion level. Changes in valine, ADMA, TMA, and several acylcarnitines indicate that higher seaweed inclusion was associated with coordinated alterations in amino acid, microbial-related, and energy metabolism. In particular, TMA, a metabolite commonly associated with microbial co-metabolism, may indicate a link between seaweed intake and host-microbial metabolic activity. This is consistent with previous reports showing that seaweed supplementation can alter microbial communities and promote beneficial bacteria (Suvega and Arunkumar, 2019). Likewise, the enrichment of pyrimidine metabolism and valine biosynthesis suggests broader changes in nucleotide and amino acid homeostasis. Similar patterns have been reported in dairy cows, where branched-chain amino acid supplementation affected phosphocholine and glutathione metabolism, linking amino acid metabolism to lipid and antioxidant pathways (Xu et al., 2022).

Trans-4-hydroxyproline also varied across methane and seaweed groups. The highest concentration was observed in high methane-emitting animals at 0.0% seaweed, whereas the lowest value occurred in animals with moderate methane emissions at 1.0% seaweed. Overall, higher methane levels tended to be associated with elevated hydroxyproline, whereas lower methane or higher seaweed groups showed lower or intermediate values. Because hydroxyproline is linked to collagen turnover, amino acid metabolism, and oxidative stress, these differences may reflect altered protein turnover or tissue remodeling across methane phenotypes (Li and Wu, 2018). The lower values observed in lower-emission or higher-seaweed groups may also be consistent with improved nutrient utilization or antioxidant effects of seaweed polyphenols (Rajauria et al., 2021). However, given the variability across groups, hydroxyproline should be interpreted as a candidate biomarker rather than as definitive evidence of a specific mechanism.

Branched-chain amino acids such as valine, together with metabolites linked to nitrogen homeostasis such as ADMA and urea, were especially prominent in the higher seaweed groups. Valine was identified across all five statistical tests in the 0.0% vs. 2.0% comparison, whereas ADMA, a nitric oxide pathway inhibitor, may reflect broader changes in protein turnover and vascular-related metabolism. These observations are consistent with a previous report that antioxidant-active dietary components can influence oxidative status during methane mitigation (Min et al., 2020). Homoarginine, which discriminated the 0.5% vs. 1.0% comparison, may further suggest that arginine-related metabolism is associated with the transition from weak to more effective methane mitigation, although this relationship remains associative rather than mechanistic.

Future studies may focus on determining whether these amino acid-related shifts are stable over time and whether they predict long-term methane phenotype, productivity, or response to dietary supplementation. In that context, metabolomics may help identify early metabolic indicators of response to methane-mitigation strategies in heifers.

### Energy metabolism

The plasma metabolomic patterns associated with methane emission groups in replacement heifers suggest links between microbial activity, host metabolism, and methane production. Rather than reflecting digestion alone, methane emission phenotype appeared to be associated with broader metabolic differences related to energy utilization.

Oxalic acid was higher in low-CH_4_ groups than in the higher-CH_4_ groups. Because oxalic acid is a byproduct of the catabolism of amino acids such as glycine and hydroxyproline (Porter, 2012), this pattern may reflect altered hydrogen utilization and fermentation balance in lower methane-emitting animals. This interpretation is consistent with recent *in vitro* evidence showing that oxalic acid can modify rumen fermentation by shifting hydrogen away from methanogenesis toward alternative sinks, increasing propionate and butyrate production while reducing the acetate-to-propionate ratio (Aschalew et al., 2024). Oxalic acid has also been associated with increased abundances of propionigenic genera such as *Prevotella, Ruminococcus,* and *Sharpea*, which may further support competing hydrogen-utilization pathways (Aschalew et al., 2024). Although our results do not establish a direct inhibitory role for oxalic acid, they are consistent with the possibility that oxalic acid is associated with fermentation patterns linked to reduced methane production.

High methane-emitting heifers at the 0.0% and 0.5% seaweed inclusion levels also showed elevated microbial-derived short-chain fatty acids, particularly propionic acid and caproic acid, together with higher urea and ethylmalonic acid. These alterations may indicate less efficient energy harvesting or altered fermentation patterns associated with greater methanogenesis. Elevated ethylmalonic acid may reflect impaired energy metabolism or a metabolic cost associated with inefficient hydrogen disposal. This broader interpretation is consistent with previous reports that changes in fermentation end products accompany shifts in methane production (Busquet et al., 2005).

Lipid-related differences were also evident across methane emission groups. Low-emitting heifers showed higher concentrations of medium-to long-chain triglycerides (C34–C40) and phosphatidylcholines (PC ae C36.5–C40.5), which may be consistent with more efficient energy handling. This observation aligns with the enrichment of pantothenate and CoA biosynthesis in the Low vs. Moderate comparison, a pathway relevant to fatty acid metabolism and mitochondrial energy handling. In contrast, high emitters showed accumulation of linoleic-acid-enriched triglycerides and dihexosylceramides, which may indicate greater metabolic stress or altered lipid signaling. This interpretation is broadly consistent with reports linking sphingolipid profiles to metabolic health (Park et al., 2025).

Nitrogen metabolism also differentiated methane emission phenotypes. Urea was consistently higher in the high-emitter groups, suggesting less efficient nitrogen recycling and possibly reduced microbial protein synthesis. This is consistent with findings in Japanese Black steers, where methane-emission phenotype was associated with differences in blood urea nitrogen (Kim et al., 2022). Together with the lipid and microbial-related metabolites observed here, these results support the view that methane phenotype is linked to coordinated differences in both nitrogen and energy metabolism.

Ethylmalonic acid (EMA) and α-ketoisovaleric acid emerged as particularly relevant discriminators. EMA, often interpreted as a marker of mitochondrial stress or altered fatty acid oxidation, was elevated in high methane emitters and lower in some lower-emission groups. In contrast, α-ketoisovaleric acid differentiated Low vs. Moderate groups and may reflect altered branched-chain amino acid catabolism and substrate use. Metabolites commonly associated with fermentation, such as propionic acid and caproic acid, were enriched in moderate and high emitters, further suggesting that methane phenotype is associated with distinct fermentation-linked energy pathways. The appearance of trimethylamine and hippuric acid at 2.0% seaweed also supports a role for host–microbe co-metabolism in these energy-related responses.

Cis-aconitic acid (CAA), a TCA-cycle intermediate, also varied across methane and supplementation groups. Animals with lower methane emissions at 1.0% seaweed inclusion showed lower EMA and intermediate CAA values, whereas in high methane emitters CAA tended to decrease as seaweed supplementation increased. These patterns may indicate altered carbon flow and mitochondrial metabolism associated with methane suppression. This interpretation is consistent with the broader concept that suppressed methanogenesis can redirect hydrogen toward propionate production and thereby alter central carbon metabolism (Ungerfeld, 2020; Choi et al., 2022). However, the responses were not linear, and the increase in EMA at 2.0% despite reduced methane suggests that higher seaweed inclusion may induce adaptive shifts in hydrogen disposal or microbial metabolism rather than a simple linear improvement in energetic efficiency.

Overall, these findings suggest that the interaction between seaweed supplementation and methane emission group was associated with plasma metabolite patterns related to rumen-associated metabolism, mitochondrial metabolism, lipid handling, and nitrogen use. The identification of TGs, PCs, EMA, and CAA as candidate biomarkers provides a useful starting point for future validation studies and may ultimately help guide more targeted methane-mitigation strategies in ruminant production systems.

## Future prospects

The identification of key plasma metabolites associated with brown seaweed supplementation and methane emission levels provides several directions for future research and for advancing sustainable livestock management. First, the potential biomarkers uncovered in this study, including EMA, TMAO, triglycerides, and microbial-derived metabolites such as indole-3-propionic acid and hippuric acid, should be validated in larger and multi-breed trials to confirm their utility across diverse cattle populations. Moreover, mechanistic studies are needed to clarify how Atlantic GRO® brown seaweed components modulate lipid metabolism and gut microbial metabolism via TMA and TCA cycle intermediates in relation to methane production. Future studies should chemically profile seaweed in more detail, because the plasma metabolites identified in the present study represent systemic response markers rather than the original active compounds in the seaweed. Based on previous literature and the known composition of brown seaweeds, candidate bioactive components, including phlorotannins, polysaccharides, alginic acid, fucoidans, laminarin, mannitol, and minerals, should be identified through targeted chemical profiling and tested individually or in combination to determine their effects on rumen fermentation, microbial communities, methane production, and host metabolism. Additionally, the non-linear metabolic responses observed here between seaweed levels (minimal changes at 0.5% and the strongest changes at 2.0%) necessitate further investigations into threshold effects and long-term adaptations.

Another potential limitation of this study is that adjusted methane emission values, which are continuous traits, were classified into low, moderate, and high groups as a common practice in nutritional and metabolic studies. This strategy was intentionally applied to improve biological interpretation, facilitate comparison across different phenotypes, and support clearer visualization of metabolomic patterns associated with methane production. Although categorization can reduce statistical power, it may miss non-linear patterns or hide small variations between animals near the group cut-off points. Future studies with larger populations may build on these results by increasing sample size. Although the two-way interaction model provided useful insight into how seaweed supplementation and methane emission may jointly influence plasma metabolism, the interaction results should be interpreted in the context of the exploratory nature of this study. Therefore, studies with more animals per subgroup are suggested to validate the discriminating metabolites and confirm the robustness of the observed interaction effects.

Another limitation is that this study used only pregnant replacement heifers, which represent an important stage in beef production and are often managed on high forage diets, making them more relevant for methane mitigation research. However, sex and pregnancy status can affect circulating metabolites through changes in growth, hormones, nutrient use, lipid and amino acid metabolism, and nitrogen balance. Therefore, these findings provide useful candidate biomarkers and biological insight, their direct application to steers, bulls, non-pregnant heifers, or finishing cattle should be validated in larger and more diverse cattle populations.

Integrating multi-omics approaches, such as metagenomics to profile rumen microbiota or transcriptomics to map host metabolic pathways, could clarify potential relationships between seaweed-induced biochemical changes and methane output. Future studies should integrate plasma and rumen metabolomics with rumen fermentation measurements and advanced microbiome profiling. In particular, third-generation full-length sequencing of bacterial 16S rRNA and fungal ITS regions would provide greater taxonomic resolution than short-read amplicon sequencing and help determine how bacterial and fungal community dynamics relate to specific plasma metabolite patterns, seaweed supplementation responses, and methane output. Because metabolites such as TMA, indole-3-propionic acid, propionic acid, and caproic acid may reflect both rumen microbial activity and host metabolism, future multi-omics studies should use paired rumen and plasma samples to separate microbial from systemic responses. Combining rumen fermentation data, full-length microbial sequencing, archaeal methanogen profiling, and targeted metabolomics would help identify which microbial groups and fermentation pathways are linked to the plasma metabolite shifts and methane output observed in this study. Key discriminating metabolites, including triglycerides, phosphatidylcholines, and microbial-derived compounds, suggesting changes in lipid metabolism, nitrogen homeostasis, and host-microbial co-metabolism.

The present study was designed to evaluate whether circulating plasma metabolites could serve as practical, minimally invasive indicators of animal-level responses to seaweed supplementation and methane emission phenotype. Therefore, future studies integrating rumen fermentation data, microbiome profiling, and paired rumen–plasma metabolomics would help further define the biological pathways linking seaweed supplementation, rumen function, and methane output. Translating these metabolites into rapid, pen-side assays or portable metabolomic tools such as simplified LC–MS/MS protocols would enable real-time monitoring of dietary efficacy on farms, empowering producers to understand the effectiveness of supplementation strategies and adjust the methodology. By bridging the gap between metabolomic insights and practical applications, this work can pave the way for precision livestock management that enhances both environmental sustainability and economic viability for cattle enterprises.

This study revealed complex interactions between dietary seaweed supplementation and methane emission groups in replacement heifers, with 152 plasma metabolites significantly impacted by their interaction. Key discriminating metabolites, including triglycerides, phosphatidylcholines, and microbial-derived compounds, suggested changes in lipid metabolism, nitrogen homeostasis, and host-microbial co-metabolism. Dose-specific responses were evident with stronger metabolic changes at higher seaweed inclusion levels (1.0%–2.0%), which coincided with significant methane reduction, whereas the lower dose (0.5%) induced minimal methane reduction. Independent analyses further showed methane emission phenotypes were associated with metabolites linked to energy metabolism and microbial fermentation. Multi-method validation identified urea and EMA as strong biomarkers for distinguishing emission categories. Overall, these findings support plasma metabolomics as a useful tool for monitoring dietary interventions and understanding methane-related metabolic adaptations in cattle.

## Data Availability

The original contributions presented in the study are included in the article/Supplementary Material, further inquiries can be directed to the corresponding author.

## References

[B1] Abbott D. W. Aasen I. M. Beauchemin K. A. Grondahl F. Gruninger R. Hayes M. (2020). Seaweed and seaweed bioactives for mitigation of enteric methane: challenges and opportunities. Animals 10 (12), 2432. 10.3390/ani10122432 33353097 PMC7766277

[B2] Ahmad I. Rawnsley R. P. Bowman J. P. Omede A. A. (2025). Rumen microbiome response to methane inhibition. Microbiol. Aust. 46, 91–95. 10.1071/ma25026

[B3] Ahmed E. Batbekh B. Fukuma N. Hanada M. Nishida T. (2022). Evaluation of different brown seaweeds as feed and feed additives regarding rumen fermentation and methane mitigation. Fermentation 8 (10), 504. 10.3390/fermentation8100504

[B4] Aschalew N. D. Zhang L. Wang Z. Xia Y. Yin G. Dong J. (2024). Effects of yeast culture and oxalic acid supplementation on *in vitro* nutrient disappearance, rumen fermentation, and bacterial community composition. Front. Vet. Sci. 10, 1330841. 10.3389/fvets.2023.1330841 38313769 PMC10834634

[B5] Bica R. Palarea-Albaladejo J. Lima J. Uhrin D. Miller G. A. Bowen J. M. (2022). Methane emissions and rumen metabolite concentrations in cattle fed two different silages. Sci. Rep. 12, 5441. 10.1038/s41598-022-09108-w 35361825 PMC8971404

[B7] Borzouie S. Duynisveld J. Pal C. Fillmore S. Manafiazar G. (2026). Effects of brown seaweed supplementation on methane emissions, health, and productivity indicators in pregnant replacement heifers. Front. Anim. Sci. 10.3389/fanim.2026.1840864

[B8] Bošnjaković D. Nedić S. Arsić S. Prodanović R. Vujanac I. Jovanović L. (2024). Effects of brown seaweed (Ascophyllum nodosum) supplementation on enteric methane emissions, metabolic status and milk composition in peak-lactating holstein cows. Animals 14 (11), 1520. 10.3390/ani14111520 38891568 PMC11171174

[B9] Busquet M. Calsamiglia S. Ferret A. Kamel C. (2005). Effect of garlic oil and four of its compounds on rumen microbial fermentation. J. Dairy Sci. 88 (12), 4393–4404. 10.3168/jds.S0022-0302(05)73126-X 16291631

[B61] CCAC (2009). CCAC guidelines on: the care and use of farm animals in research, teaching and testing [Guidelines]. Canadian Council on Animal Care. Available online at: https://ccac.ca/Documents/Standards/Guidelines/Farm_Animals.pdf.

[B11] Choi Y. Lee S. J. Kim H. S. Eom J. S. Jo S. U. Guan L. L. (2022). Red seaweed extracts reduce methane production by altering rumen fermentation and microbial composition *in vitro* . Front. Vet. Sci. 9, 1012345. 10.3389/fvets.2022.985824 36467635 PMC9709288

[B13] de Ondarza M. B. Smith J. Johnson L. Tricarico J. M. (2024). Understanding potential opportunities and risks associated with feeding supplemental rumen available fats to mitigate enteric methane emissions in lactating dairy cows. J. Dairy Sci. 107 (10), 8072–8083. 10.3168/jds.2023-24528 38825101

[B14] Doreau M. Ferlay A. (1995). Effect of dietary lipids on nitrogen metabolism in the rumen: a review. Livest. Prod. Sci. 43 (2), 97–110. 10.1016/0301-6226(95)00041-i

[B15] Haque M. N. (2018). Dietary manipulation: a sustainable way to mitigate methane emissions from ruminants. J. Anim. Sci. Technol. 60, 15. 10.1186/s40781-018-0175-7 29946475 PMC6004689

[B18] Kim M. Masaki T. Ikuta K. Iwamoto E. Uemoto Y. Terada F. (2022). Changes in the liver transcriptome and physiological parameters of Japanese black steers during the fattening period. Sci. Rep. 12, 4029. 10.1038/s41598-022-08057-8 35256743 PMC8901683

[B19] Kinley R. D. de Nys R. Vucko M. J. Machado L. Tomkins N. W. (2016). The red macroalgae *Asparagopsis taxiformis* is a potent natural antimethanogenic that reduces methane production during *in vitro* fermentation with rumen fluid. Anim. Prod. Sci. 56, 282–289. 10.1071/an15576

[B21] Künzel S. Yergaliyev T. Wild K. J. Philippi H. Petursdottir A. H. Gunnlaugsdottir H. (2022). Methane reduction potential of brown seaweeds and their influence on nutrient degradation and microbiota composition in a rumen simulation technique. Front. Microbiol. 13, 889618. 10.3389/fmicb.2022.889618 35836418 PMC9273974

[B22] Law S. H. Chan M. L. Marathe G. K. Parveen F. Chen C. H. Ke L. Y. (2019). An updated review of lysophosphatidylcholine metabolism in human diseases. Int. J. Mol. Sci. 20 (5), 1149. 10.3390/ijms20051149 30845751 PMC6429061

[B24] Li P. Wu G. (2018). Roles of dietary glycine, proline, and hydroxyproline in collagen synthesis and animal growth. Amino Acids 50, 29–38. 10.1007/s00726-017-2490-6 28929384

[B25] Li H. Wang X. Vinsky M. Manafiazar G. Fitzsimmons C. Li L. (2024). Analyses of plasma metabolites using a high performance four-channel CIL LC-MS method and identification of metabolites associated with enteric methane emissions in beef cattle. PLoS ONE 19 (3), e0299268. 10.1371/journal.pone.0299268 38427676 PMC10906882

[B28] Machmüller A. Ossowski D. A. Kreuzer M. (2000). Comparative evaluation of the effects of coconut oil, oilseeds, and crystalline fat on methane release, digestion, and energy balance in lambs. Anim. Feed Sci. Technol. 85 (1-2), 41–60. 10.1016/s0377-8401(00)00126-7

[B29] Maia M. R. G. Fonseca A. J. M. Oliveira H. M. Mendonça C. Cabrita A. R. J. (2016). The potential role of seaweeds in the natural manipulation of rumen fermentation and methane production. Sci. Rep. 6, 32321. 10.1038/srep32321 27572486 PMC5004155

[B30] Matthews C. Crispie F. Lewis E. Reid M. O’Toole P. W. Cotter P. D. (2019). The rumen microbiome: a crucial consideration when optimising milk and meat production and nitrogen utilisation efficiency. Gut Microbes 10 (2), 115–132. 10.1080/19490976.2018.1505176 30207838 PMC6546327

[B31] McGurrin A. Maguire J. Tiwari B. K. Garcia-Vaquero M. (2023). Anti-methanogenic potential of seaweeds and seaweed-derived compounds in ruminant feed: current perspectives, risks, and future prospects. J. Anim. Sci. Biotechnol. 14, 145. 10.1186/s40104-023-00946-w 38041152 PMC10693045

[B32] Min B. R. Solaiman S. Waldrip H. M. Parker D. Todd R. W. Brauer D. (2020). Dietary mitigation of enteric methane emissions from ruminants: a review of plant tannin mitigation options. Anim. Nutr. 6 (3), 231–246. 10.1016/j.aninu.2020.05.002 33005757 PMC7503797

[B33] Moran D. Wall E. (2011). Livestock production and greenhouse gas emissions: defining the problem and specifying solutions. Anim. Front. 1, 19–25. 10.2527/af.2011-0012

[B35] Orzuna-Orzuna J. F. Lara-Bueno A. Mendoza-Martínez G. D. Miranda-Romero L. A. Vázquez Silva G. de la Torre-Hernández M. E. (2024). Meta-analysis of dietary supplementation with seaweed in dairy cows: milk yield and composition, nutrient digestibility, rumen fermentation, and enteric methane emissions. Dairy 5 (3), 464–479. 10.3390/dairy5030036

[B36] Pang Z. Lu Y. Zhou G. Hui F. Xu L. Viau C. (2024). MetaboAnalyst 6.0: towards a unified platform for metabolomics data processing, analysis and interpretation. Nucleic Acids Res. 52 (W1), W398–W406. 10.1093/nar/gkae253 38587201 PMC11223798

[B37] Park D. Kim H. Shin H. H. Imm J. Y. (2025). Dietary sphingolipids and milk fat globule membrane: emerging roles in cardiometabolic health and muscle function. Food Sci. Biotechnol. 34, 3473–3486. 10.1007/s10068-025-01863-6 41113271 PMC12528607

[B38] Porter R. S. (2012). “Oxalic acid metabolism,” in The Merck Manual of Diagnosis and Therapy. 19th ed. (Whitehouse Station, NJ: Merck Sharp and Dohme Corp.), 1234–1236.

[B40] Rajauria G. Ravindran R. Garcia-Vaquero M. Rai D. K. Sweeney T. O’Doherty J. (2021). Molecular characteristics and antioxidant activity of laminarin extracted from the seaweed species laminaria hyperborea, using hydrothermal-assisted extraction and a multi-step purification procedure. Food Hydrocoll. 112, 106332. 10.1016/j.foodhyd.2020.106332

[B41] Rasmussen J. Harrison A. (2011). The benefits of supplementary fat in feed rations for ruminants with particular focus on reducing levels of methane production. ISRN Vet. Sci. 2011, 613172. 10.5402/2011/613172 23738103 PMC3658489

[B43] Roque B. M. Salwen J. K. Kinley R. Kebreab E. (2019). Inclusion of *Asparagopsis armata* in lactating dairy cows’ diet reduces enteric methane emission by over 50 percent. J. Clean. Prod. 234, 132–138. 10.1016/j.jclepro.2019.06.193

[B46] Suvega T. Arunkumar K. (2019). Probiotic bacteria promote the growth of associating host (red seaweed, Gracilaria edulis) also synthesize antibacterial protein. Biocatal. Agric. Biotechnol. 19, 101136. 10.1016/j.bcab.2019.101136

[B47] Tachibana C. (2014). What’s next in ’omics: the metabolome. Science 345 (6203), 1519–1521. 10.1126/science.345.6203.1519

[B48] Ungerfeld E. M. (2020). Metabolic hydrogen flows in rumen fermentation: principles and possibilities of interventions. Front. Microbiol. 11, 589. 10.3389/fmicb.2020.00589 32351469 PMC7174568

[B49] Vargas J. E. Andrés S. López-Ferreras L. Snelling T. J. Yáñez-Ruíz D. R. García-Estrada C. (2020). Dietary supplemental plant oils reduce methanogenesis from anaerobic microbial fermentation in the rumen. Sci. Rep. 10, 1613. 10.1038/s41598-020-58401-z 32005859 PMC6994681

[B50] Vasta V. Daghio M. Cappucci A. Buccioni A. Serra A. Viti C. (2019). Invited review: plant polyphenols and rumen microbiota responsible for fatty acid biohydrogenation, fiber digestion, and methane emission – experimental evidence and methodological approaches. J. Dairy Sci. 102 (5), 3781–3804. 10.3168/jds.2018-14985 30904293

[B51] Vijn S. Compart D. P. Dutta N. Foukis A. Hess M. Hristov A. N. (2020). Key considerations for the use of seaweed to reduce enteric methane emissions from cattle. Front. Vet. Sci. 7, 597430. 10.3389/fvets.2020.597430 33426018 PMC7785520

[B52] Wang Y. Xuemei N. Zhao Y. Jiang L. Wang H. Hua D. (2021). Dietary supplementation with inulin improves lactation performance and serum lipids by regulating the rumen microbiome and metabolome in dairy cows. Anim. Nutr. 7, 1189–1204. 10.1016/j.aninu.2021.09.007 34754961 PMC8556608

[B54] Wu G. Bazer F. W. Dai Z. Li D. Wang J. Wu Z. (2016). Amino acid nutrition in animals: protein synthesis and beyond. Annu. Rev. Anim. Biosci. 4, 387–417. 10.1146/annurev-animal-022513-114113 25384149

[B55] Xia J. Wishart D. S. (2010). MSEA: a web-based tool to identify biologically meaningful patterns in quantitative metabolomic data. Nucleic Acids Res. 38 (Web Server issue), W71–W77. 10.1093/nar/gkq329 20457745 PMC2896187

[B56] Xia J. Psychogios N. Young N. Wishart D. S. (2009). MetaboAnalyst: a web server for metabolomic data analysis and interpretation. Nucleic Acids Res. 37 (Web Server issue), W652–W660. 10.1093/nar/gkp356 19429898 PMC2703878

[B57] Xia J. Broadhurst D. I. Wilson M. Wishart D. S. (2013). Translational biomarker discovery in clinical metabolomics: an introductory tutorial. Metabolomics 9 (2), 280–299. 10.1007/s11306-012-0482-9 23543913 PMC3608878

[B58] Xu W. Kenéz A. Mann S. Overton T. R. Joseph J. Nydam D. V. (2022). Effects of dietary branched-chain amino acid supplementation on serum and milk metabolome profiles in dairy cows during early lactation. J. Dairy Sci. 105 (10), 8497–8508. 10.3168/jds.2022-21892 35965128

[B59] Yanibada B. Hohenester U. Pétéra M. Canlet C. Durand S. Jourdan F. (2020). Inhibition of enteric methanogenesis in dairy cows induces changes in plasma metabolome highlighting metabolic shifts and potential markers of emission. Sci. Rep. 10, 15591. 10.1038/s41598-020-72145-w 32973203 PMC7515923

[B60] Zhang L. Zheng J. Johnson M. Mandal R. Cruz M. Martínez-Huélamo M. (2024). A comprehensive LC-MS metabolomics assay for quantitative analysis of serum and plasma. Metabolites 14 (11), 622. 10.3390/metabo14110622 39590858 PMC11596266

