# Supplementary material for: Metabolites associated with the inclusion of atlantic GRO® seaweed supplementation as a methane mitigation strategy in the diet of replacement beef heifers
Source: Front Mol Biosci. 2026 Jul 29;13:1866940. doi: 10.3389/fmolb.2026.1866940 (PMC13461535; doi:10.3389/fmolb.2026.1866940)
Supplement: Supplementary file 1 [file Table1.docx]

**Supplementary Materials**

**Table S1.** Optimized MRM transitions and mass spectrometer parameters for targeted metabolites

| **Metabolites ^1^** | **MRM ^2^ Parameters** | | | | |  |
| --- | --- | --- | --- | --- | --- | --- |
|  | **Q1 ^3^** | **Q3 ^4^** | **DP ^5^ (V ^6^)** | **CE ^7^ (V)** | **CXP ^8^ (V)** | |
| 7-Methylguanine | 166.1 | 149 | 132 | 29 | 13 | |
| alpha-Aminobutyric acid | 239.1 | 104 | 45 | 16 | 15 | |
| Adenosine | 268.2 | 136.1 | 87 | 28 | 12 | |
| Asymmetric dimethylarginine | 338.2 | 46 | 71 | 61 | 15 | |
| Dimethylarginine | 76 | 58 | 40 | 26 | 15 | |
| Alanine | 225.2 | 44.2 | 61 | 35 | 15 | |
| Allantoin | 159 | 116 | 83 | 11 | 15 | |
| alpha-Aminoadipic acid | 297.1 | 144.2 | 46 | 23 | 15 | |
| Arginine | 310 | 217 | 76 | 23 | 15 | |
| Asparagine | 268.2 | 87 | 41 | 25 | 15 | |
| Aspartic acid | 269.2 | 116.2 | 76 | 21 | 15 | |
| beta-Alanine | 225.1 | 90.1 | 41 | 16 | 15 | |
| Betaine | 118.1 | 59.2 | 60 | 30 | 15 | |
| Carnosine | 362.2 | 110.1 | 46 | 47 | 15 | |
| Choline | 104.2 | 60.1 | 28 | 50 | 15 | |
| Citrulline | 311.2 | 113.1 | 56 | 29 | 15 | |
| Creatine | 132.1 | 90 | 40 | 18 | 15 | |
| Creatinine | 114.1 | 44 | 60 | 17 | 15 | |
| Cystathionine | 358.1 | 134.1 | 60 | 29 | 15 | |
| Cytidine | 244.2 | 112.1 | 50 | 22 | 8 | |
| Deoxycytidine | 228.1 | 112 | 61 | 23 | 13 | |
| Glutamine | 282.2 | 130 | 71 | 25 | 15 | |
| Glutamic acid | 283.2 | 130.2 | 76 | 23 | 15 | |
| Glycine | 211.2 | 75.9 | 66 | 14 | 15 | |
| Histidine | 291.1 | 110.2 | 56 | 33 | 15 | |
| Homoarginine | 324.1 | 84 | 112 | 46 | 12 | |
| Homocitrulline | 325 | 127 | 56 | 29 | 10 | |
| Isoleucine | 267.3 | 69 | 55 | 45 | 15 | |
| Indole | 118.1 | 91 | 105 | 30 | 10 | |
| Kynurenine | 344.2 | 146.2 | 46 | 33 | 15 | |
| Leucine | 267.3 | 43 | 55 | 66 | 15 | |
| Lysine | 417.2 | 324.2 | 81 | 19 | 15 | |
| Methionine | 285.1 | 104.2 | 71 | 25 | 15 | |
| Methionine sulfoxide | 301.2 | 88.1 | 61 | 41 | 15 | |
| Methylhistidine | 305.1 | 212.1 | 46 | 23 | 15 | |
| N2-Acetyl-ornithine | 310.2 | 217.3 | 46 | 15 | 15 | |
| Ornithine | 403.2 | 310.2 | 76 | 19 | 15 | |
| Phenylalanine | 301.2 | 120.2 | 71 | 31 | 15 | |
| Proline | 251.2 | 70.3 | 51 | 43 | 15 | |
| Sarcosine | 225.1 | 44.2 | 43 | 31 | 15 | |
| Serine | 241.2 | 60 | 41 | 31 | 15 | |
| Serotonin | 312.3 | 160.2 | 66 | 27 | 15 | |
| Taurine | 261 | 126.1 | 71 | 21 | 15 | |
| Threonine | 255.2 | 74.1 | 66 | 29 | 15 | |
| Thymidine | 243.1 | 127.1 | 50 | 17 | 12 | |
| Trimethylamine N-oxide | 267.2 | 68 | 56 | 61 | 15 | |
| trans-4-Hydroxyproline | 338.2 | 70.1 | 71 | 61 | 15 | |
| Tryptophan | 340.2 | 188.2 | 96 | 25 | 15 | |
| Tyrosine | 317.2 | 136.1 | 76 | 31 | 15 | |
| Uridine | 245.1 | 113 | 80 | 21 | 17 | |
| Valine | 253.2 | 72.2 | 81 | 31 | 15 | |
| Urea | 61 | 44 | 80 | 26 | 10 | |
| Glucose | 198.13 | 180.13 | 35 | 9 | 10 | |
| LysoPC a C14.0 | 468.3 | 184 | 79 | 30 | 15 | |
| LysoPC a C16.1 | 494.3 | 184 | 79 | 30 | 15 | |
| LysoPC a C16.0 | 496.3 | 184 | 81 | 31 | 15 | |
| LysoPC a C17.0 | 510.3 | 184 | 82 | 31 | 15 | |
| LysoPC a C18.2 | 520.3 | 184 | 82 | 31 | 15 | |
| LysoPC a C18.1 | 522.3 | 184 | 82 | 31 | 15 | |
| LysoPC a C18.0 | 524.3 | 184 | 85 | 31 | 15 | |
| LysoPC a C20.4 | 544.3 | 184 | 85 | 31 | 15 | |
| LysoPC a C24.0 | 608.4 | 184 | 96 | 33 | 15 | |
| LysoPC a C28.1 | 662.5 | 184 | 125 | 53 | 15 | |
| LysoPC a C28.0 | 664.5 | 184 | 105 | 35 | 15 | |
| SM OH) C14.1 | 689.6 | 184 | 108 | 36 | 15 | |
| SM C16.1 | 701.6 | 184 | 111 | 37 | 15 | |
| SM C16.0 | 703.6 | 184 | 112 | 37 | 15 | |
| SM(OH) C16.1 | 717.6 | 184 | 114 | 38 | 15 | |
| SM C18.1 | 729.6 | 184 | 117 | 38 | 15 | |
| PC aa C32.2 | 730.5 | 184 | 117 | 38 | 15 | |
| SM C18.0 | 731.6 | 184 | 117 | 38 | 15 | |
| SM C20.2 | 755.6 | 184 | 123 | 40 | 15 | |
| PC ae C36.0 | 776.7 | 184 | 128 | 42 | 15 | |
| PC aa C36.6 | 778.5 | 184 | 128 | 42 | 15 | |
| PC aa C36.0 | 790.6 | 184 | 131 | 43 | 15 | |
| SM(OH) C22.2 | 799.7 | 184 | 133 | 43 | 15 | |
| SM(OH) C22.1 | 801.7 | 184 | 134 | 43 | 15 | |
| PC aa C38.6 | 806.6 | 184 | 135 | 44 | 15 | |
| PC aa C38.0 | 818.7 | 184 | 138 | 45 | 15 | |
| PC ae C40.6 | 820.6 | 184 | 139 | 45 | 15 | |
| SM(OH) C24.1 | 829.7 | 184 | 141 | 46 | 15 | |
| PC aa C40.6 | 834.6 | 184 | 143 | 46 | 15 | |
| PC aa C40.2 | 842.7 | 184 | 144 | 47 | 15 | |
| PC aa C40.1 | 844.7 | 184 | 145 | 47 | 15 | |
| C0 | 162.1 | 85.1 | 61 | 27 | 15 | |
| C2 | 204.1 | 85.1 | 41 | 27 | 15 | |
| C3 | 218.1 | 85.1 | 46 | 29 | 15 | |
| C4.1 | 230.1 | 85.1 | 52 | 29 | 15 | |
| C4 | 232.2 | 85.1 | 46 | 29 | 15 | |
| C5 | 246.2 | 85.1 | 46 | 29 | 15 | |
| C4OH | 248.1 | 85.1 | 55 | 32 | 15 | |
| C5OH | 262.2 | 85.1 | 58 | 33 | 15 | |
| C8 | 288.2 | 85.1 | 66 | 33 | 15 | |
| C5MDC | 290.2 | 85.1 | 63 | 37 | 15 | |
| C9 | 302.2 | 85.1 | 66 | 39 | 15 | |
| C7DC | 304.2 | 85.1 | 66 | 39 | 15 | |
| C10.2 | 312.2 | 85.1 | 67 | 40 | 15 | |
| C14 | 372.2 | 85.1 | 86 | 45 | 15 | |
| C12DC | 374.3 | 85.1 | 86 | 45 | 15 | |
| C16.1 | 398.3 | 85.1 | 84 | 51 | 15 | |
| C16 | 400.3 | 85.1 | 84 | 51 | 15 | |
| C18.2 | 424.3 | 85.1 | 89 | 54 | 15 | |
| C18.1 | 426.4 | 85.1 | 89 | 55 | 15 | |
| C18 | 428.4 | 85.1 | 96 | 63 | 15 | |
| PC aa C28.1 | 676.5 | 184 | 106 | 35 | 15 | |
| PC ae C30.2 | 688.5 | 184 | 108 | 36 | 15 | |
| PC ae C30.1 | 690.5 | 184 | 109 | 36 | 15 | |
| PC ae C30.0 | 692.6 | 184 | 111 | 37 | 15 | |
| PC aa C30.0 | 706.5 | 184 | 114 | 37 | 15 | |
| PC ae C32.2 | 716.6 | 184 | 114 | 37 | 15 | |
| PC ae C32.1 | 718.6 | 184 | 115 | 38 | 15 | |
| PC aa C32.3 | 728.5 | 184 | 117 | 38 | 15 | |
| PC aa C32.1 | 732.6 | 184 | 118 | 39 | 15 | |
| PC aa C32.0 | 734.6 | 184 | 119 | 39 | 15 | |
| PC ae C34.3 | 742.6 | 184 | 120 | 39 | 15 | |
| PC ae C34.2 | 744.6 | 184 | 120 | 39 | 15 | |
| PC ae C34.1 | 746.6 | 184 | 121 | 39 | 15 | |
| PC ae C34.0 | 748.6 | 184 | 122 | 40 | 15 | |
| PC aa C34.4 | 754.5 | 184 | 122 | 40 | 15 | |
| PC aa C34.3 | 756.6 | 184 | 123 | 40 | 15 | |
| PC aa C34.2 | 758.6 | 184 | 123 | 40 | 15 | |
| PC aa C34.1 | 760.6 | 184 | 125 | 41 | 15 | |
| PC ae C36.5 | 766.6 | 184 | 125 | 41 | 15 | |
| PC ae C36.4 | 768.6 | 184 | 126 | 41 | 15 | |
| PC ae C36.3 | 770.6 | 184 | 126 | 41 | 15 | |
| PC ae C36.2 | 772.6 | 184 | 127 | 41 | 15 | |
| PC ae C36.1 | 774.6 | 184 | 127 | 41 | 15 | |
| PC aa C36.5 | 780.6 | 184 | 128 | 42 | 15 | |
| PC aa C36.4 | 782.6 | 184 | 129 | 42 | 15 | |
| PC aa C36.3 | 784.6 | 184 | 130 | 42 | 15 | |
| PC aa C36.2 | 786.6 | 184 | 130 | 42 | 15 | |
| PC aa C36.1 | 788.6 | 184 | 131 | 43 | 15 | |
| PC ae C38.6 | 792.6 | 184 | 132 | 43 | 15 | |
| PC ae C38.5 | 794.6 | 184 | 132 | 43 | 15 | |
| PC ae C38.4 | 796.6 | 184 | 133 | 43 | 15 | |
| PC ae C38.3 | 798.6 | 184 | 133 | 43 | 15 | |
| PC ae C38.2 | 800.7 | 184 | 133 | 43 | 15 | |
| PC ae C38.1 | 802.7 | 184 | 134 | 44 | 15 | |
| PC ae C38.0 | 804.7 | 184 | 135 | 44 | 15 | |
| PC aa C38.5 | 808.6 | 184 | 136 | 44 | 15 | |
| PC aa C38.4 | 810.6 | 184 | 136 | 44 | 15 | |
| PC aa C38.3 | 812.6 | 184 | 136 | 44 | 15 | |
| SM C24.1 | 813.7 | 184 | 137 | 45 | 15 | |
| SM C24.0 | 815.7 | 184 | 137 | 45 | 15 | |
| PC aa C38.1 | 816.7 | 184 | 138 | 45 | 15 | |
| PC ae C40.5 | 822.6 | 184 | 139 | 45 | 15 | |
| PC ae C40.4 | 824.7 | 184 | 140 | 45 | 15 | |
| PC ae C40.3 | 826.7 | 184 | 140 | 46 | 15 | |
| PC ae C40.2 | 828.7 | 184 | 141 | 46 | 15 | |
| PC ae C40.1 | 830.7 | 184 | 141 | 46 | 15 | |
| PC aa C40.5 | 836.6 | 184 | 143 | 47 | 15 | |
| PC aa C40.4 | 838.6 | 184 | 144 | 47 | 15 | |
| PC aa C40.3 | 840.7 | 184 | 144 | 47 | 15 | |
| SM C26.1 | 841.7 | 184 | 144 | 47 | 15 | |
| SM C26.0 | 843.7 | 184 | 145 | 47 | 15 | |
| PC ae C42.5 | 850.7 | 184 | 147 | 48 | 15 | |
| PC ae C42.4 | 852.7 | 184 | 148 | 48 | 15 | |
| PC ae C42.3 | 854.7 | 184 | 148 | 48 | 15 | |
| PC ae C42.2 | 856.7 | 184 | 149 | 48 | 15 | |
| PC ae C42.1 | 858.7 | 184 | 141 | 46 | 15 | |
| PC ae C42.0 | 860.8 | 184 | 150 | 49 | 15 | |
| PC aa C42.6 | 862.6 | 184 | 150 | 49 | 15 | |
| PC aa C42.5 | 864.7 | 184 | 151 | 49 | 15 | |
| PC aa C42.4 | 866.7 | 184 | 152 | 50 | 15 | |
| PC aa C42.2 | 870.7 | 184 | 153 | 50 | 15 | |
| PC aa C42.1 | 872.7 | 184 | 153 | 50 | 15 | |
| PC aa C42.0 | 874.7 | 184 | 154 | 50 | 15 | |
| PC ae C44.6 | 876.7 | 184 | 154 | 50 | 15 | |
| PC ae C44.5 | 878.7 | 184 | 155 | 51 | 15 | |
| PC ae C44.4 | 880.7 | 184 | 156 | 51 | 15 | |
| PC ae C44.3 | 882.7 | 184 | 156 | 51 | 15 | |
| Cer(d18.1/16.0) | 520.509 | 264.245 | 206 | 33 | 10 | |
| Cer(d18.1/22.0) | 604.603 | 264.245 | 246 | 39 | 10 | |
| Cer(d18.1/23.0) | 618.618 | 264.245 | 246 | 39 | 10 | |
| Cer(d18.1/24.0) | 632.634 | 264.245 | 256 | 37 | 10 | |
| Cer(d18.1/24.1) | 630.618 | 264.245 | 256 | 37 | 10 | |
| CE(14.0) | 614.587 | 369.352 | 41 | 19 | 10 | |
| CE(14.1) | 612.571 | 369.352 | 41 | 19 | 10 | |
| CE(15.0) | 628.603 | 369.352 | 41 | 19 | 10 | |
| CE(15.1) | 626.587 | 369.352 | 41 | 19 | 10 | |
| CE(16.0) | 642.618 | 369.352 | 41 | 19 | 10 | |
| CE(16.1) | 640.603 | 369.352 | 41 | 19 | 10 | |
| CE(17.0) | 656.634 | 369.352 | 71 | 21 | 10 | |
| CE(17.1) | 654.618 | 369.352 | 72 | 22 | 10 | |
| CE(18.0) | 670.65 | 369.352 | 73 | 23 | 10 | |
| CE(18.1) | 668.634 | 369.352 | 56 | 19 | 10 | |
| CE(18.2) | 666.618 | 369.352 | 56 | 21 | 10 | |
| CE(18.3) | 664.603 | 369.352 | 56 | 21 | 10 | |
| CE(20.0) | 698.681 | 369.352 | 56 | 21 | 10 | |
| CE(20.1) | 696.665 | 369.352 | 56 | 21 | 10 | |
| CE(20.3) | 692.634 | 369.352 | 56 | 21 | 10 | |
| CE(20.4) | 690.618 | 369.352 | 61 | 19 | 10 | |
| CE(20.5) | 688.603 | 369.352 | 61 | 19 | 10 | |
| CE(22.5) | 716.634 | 369.352 | 61 | 21 | 10 | |
| CE(22.6) | 714.618 | 369.352 | 61 | 21 | 10 | |
| Hex2Cer(d18.1/16.0) | 862.625 | 264.245 | 131 | 51 | 10 | |
| Hex2Cer(d18.1/18.0) | 890.656 | 264.245 | 131 | 52 | 10 | |
| HexCer(d18.1/23.0) | 798.682 | 264.245 | 136 | 45 | 10 | |
| HexCer(d18.1/24.0) | 812.697 | 264.245 | 136 | 46 | 10 | |
| TG.14:0_32:2 | 792.708 | 547.472 | 126 | 31 | 10 | |
| TG.14:0_34:0 | 824.77 | 579.535 | 131 | 33 | 10 | |
| TG.14:0_34:1 | 822.755 | 577.519 | 131 | 33 | 10 | |
| TG.14:0_34:2 | 820.739 | 575.503 | 131 | 33 | 10 | |
| TG.14:0_35:1 | 836.77 | 591.535 | 131 | 34 | 10 | |
| TG.14:0_35:2 | 834.755 | 589.519 | 131 | 34 | 10 | |
| TG.14:0_36:1 | 850.786 | 605.55 | 144 | 35 | 10 | |
| TG.14:0_36:2 | 848.77 | 603.535 | 144 | 35 | 10 | |
| TG.14:0_36:3 | 846.755 | 601.519 | 144 | 35 | 10 | |
| TG.14:0_36:4 | 844.739 | 599.503 | 144 | 35 | 10 | |
| TG.14:0_38:5 | 870.755 | 625.519 | 144 | 35 | 10 | |
| TG.16:0_28:1 | 766.692 | 493.425 | 126 | 30 | 10 | |
| TG.16:0_32:0 | 824.77 | 551.503 | 131 | 33 | 10 | |
| TG.16:0_32:1 | 822.755 | 549.488 | 131 | 33 | 10 | |
| TG.16:0_32:2 | 820.739 | 547.472 | 131 | 33 | 10 | |
| TG.16:0_32:3 | 818.723 | 545.456 | 131 | 33 | 10 | |
| TG.16:0_33:1 | 836.77 | 563.503 | 131 | 34 | 10 | |
| TG.16:0_33:2 | 834.755 | 561.488 | 131 | 34 | 10 | |
| TG.16:0_34:0 | 852.801 | 579.535 | 161 | 35 | 10 | |
| TG.16:0_34:1 | 850.786 | 577.519 | 161 | 33 | 10 | |
| TG.16:0_34:2 | 848.77 | 575.503 | 126 | 35 | 10 | |
| TG.16:0_34:3 | 846.755 | 573.488 | 144 | 35 | 10 | |
| TG.16:0_34:4 | 844.739 | 571.472 | 144 | 35 | 10 | |
| TG.16:0_35:1 | 864.801 | 591.535 | 144 | 35 | 10 | |
| TG.16:0_35:2 | 862.786 | 589.519 | 144 | 35 | 10 | |
| TG.16:0_35:3 | 860.77 | 587.503 | 144 | 35 | 10 | |
| TG.16:0_36:2 | 876.801 | 603.535 | 144 | 35 | 10 | |
| TG.16:0_36:3 | 874.786 | 601.519 | 144 | 35 | 10 | |
| TG.16:0_36:4 | 872.77 | 599.503 | 144 | 35 | 10 | |
| TG.16:0_36:5 | 870.755 | 597.488 | 144 | 35 | 10 | |
| TG.16:0_36:6 | 868.739 | 595.472 | 144 | 35 | 10 | |
| TG.16:0_37:3 | 888.801 | 615.535 | 144 | 36 | 10 | |
| TG.16:0_38:1 | 906.848 | 633.582 | 161 | 37 | 10 | |
| TG.16:0_38:2 | 904.833 | 631.566 | 161 | 37 | 10 | |
| TG.16:0_38:3 | 902.817 | 629.55 | 161 | 37 | 10 | |
| TG.16:0_38:4 | 900.801 | 627.535 | 161 | 37 | 10 | |
| TG.16:0_38:5 | 898.786 | 625.519 | 161 | 37 | 10 | |
| TG.16:0_38:6 | 896.77 | 623.503 | 161 | 37 | 10 | |
| TG.16:0_38:7 | 894.755 | 621.488 | 161 | 37 | 10 | |
| TG.16:0_40:6 | 924.801 | 651.535 | 161 | 39 | 10 | |
| TG.16:0_40:7 | 922.786 | 649.519 | 161 | 39 | 10 | |
| TG.16:0_40:8 | 920.77 | 647.503 | 161 | 39 | 10 | |
| TG.16:1_28:0 | 766.692 | 495.441 | 126 | 30 | 10 | |
| TG.16:1_32:0 | 822.755 | 551.503 | 131 | 33 | 10 | |
| TG.16:1_32:1 | 820.739 | 549.488 | 131 | 33 | 10 | |
| TG.16:1_32:2 | 818.723 | 547.472 | 131 | 33 | 10 | |
| TG.16:1_33:1 | 834.755 | 563.503 | 131 | 34 | 10 | |
| TG.16:1_34:0 | 850.786 | 579.535 | 144 | 35 | 10 | |
| TG.16:1_34:1 | 848.77 | 577.519 | 144 | 35 | 10 | |
| TG.16:1_34:2 | 846.755 | 575.503 | 144 | 35 | 10 | |
| TG.16:1_34:3 | 844.739 | 573.488 | 144 | 35 | 10 | |
| TG.16:1_36:1 | 876.801 | 605.55 | 144 | 35 | 10 | |
| TG.16:1_36:2 | 874.786 | 603.535 | 144 | 35 | 10 | |
| TG.16:1_36:3 | 872.77 | 601.519 | 144 | 35 | 10 | |
| TG.16:1_36:4 | 870.755 | 599.503 | 144 | 35 | 10 | |
| TG.16:1_38:5 | 896.77 | 625.519 | 161 | 37 | 10 | |
| TG.17:0_32:1 | 836.77 | 549.488 | 131 | 34 | 10 | |
| TG.17:0_34:1 | 864.801 | 577.519 | 144 | 35 | 10 | |
| TG.17:0_34:2 | 862.786 | 575.503 | 144 | 35 | 10 | |
| TG.17:0_34:3 | 860.77 | 573.488 | 144 | 35 | 10 | |
| TG.17:0_36:3 | 888.801 | 601.519 | 144 | 36 | 10 | |
| TG.17:0_36:4 | 886.786 | 599.503 | 144 | 36 | 10 | |
| TG.17:1_32:1 | 834.755 | 549.488 | 131 | 34 | 10 | |
| TG.17:1_34:1 | 862.786 | 577.519 | 144 | 35 | 10 | |
| TG.17:1_34:2 | 860.77 | 575.503 | 144 | 35 | 10 | |
| TG.17:1_34:3 | 858.755 | 573.488 | 144 | 35 | 10 | |
| TG.17:1_36:3 | 886.786 | 601.519 | 144 | 36 | 10 | |
| TG.18:0_30:0 | 824.77 | 523.472 | 131 | 33 | 10 | |
| TG.18:0_30:1 | 822.755 | 521.456 | 131 | 33 | 10 | |
| TG.18:0_32:0 | 852.801 | 551.503 | 144 | 35 | 10 | |
| TG.18:0_32:1 | 850.786 | 549.488 | 144 | 35 | 10 | |
| TG.18:0_32:2 | 848.77 | 547.472 | 144 | 35 | 10 | |
| TG.18:0_34:2 | 876.801 | 575.503 | 144 | 35 | 10 | |
| TG.18:0_34:3 | 874.786 | 573.488 | 144 | 35 | 10 | |
| TG.18:0_36:1 | 906.848 | 605.55 | 116 | 37 | 10 | |
| TG.18:0_36:2 | 904.833 | 603.535 | 161 | 37 | 10 | |
| TG.18:0_36:3 | 902.817 | 601.519 | 161 | 37 | 10 | |
| TG.18:0_36:4 | 900.801 | 599.503 | 161 | 37 | 10 | |
| TG.18:0_36:5 | 898.786 | 597.488 | 161 | 37 | 10 | |
| TG.18:0_38:6 | 924.801 | 623.503 | 161 | 39 | 10 | |
| TG.18:0_38:7 | 922.786 | 621.488 | 161 | 39 | 10 | |
| TG.18:1_26:0 | 766.692 | 467.409 | 126 | 30 | 10 | |
| TG.18:1_28:1 | 792.708 | 493.425 | 126 | 31 | 10 | |
| TG.18:1_30:0 | 822.755 | 523.472 | 131 | 33 | 10 | |
| TG.18:1_30:1 | 820.739 | 521.456 | 131 | 33 | 10 | |
| TG.18:1_30:2 | 818.723 | 519.441 | 131 | 33 | 10 | |
| TG.18:1_31:0 | 836.77 | 537.488 | 131 | 34 | 10 | |
| TG.18:1_32:0 | 850.786 | 551.503 | 144 | 35 | 10 | |
| TG.18:1_32:1 | 848.77 | 549.488 | 144 | 35 | 10 | |
| TG.18:1_32:2 | 846.755 | 547.472 | 144 | 35 | 10 | |
| TG.18:1_32:3 | 844.739 | 545.456 | 144 | 35 | 10 | |
| TG.18:1_33:0 | 864.801 | 565.519 | 144 | 35 | 10 | |
| TG.18:1_33:1 | 862.786 | 563.503 | 144 | 35 | 10 | |
| TG.18:1_33:2 | 860.77 | 561.488 | 144 | 35 | 10 | |
| TG.18:1_34:1 | 876.801 | 577.519 | 144 | 35 | 10 | |
| TG.18:1_34:2 | 874.786 | 575.503 | 144 | 35 | 10 | |
| TG.18:1_34:3 | 872.77 | 573.488 | 144 | 35 | 10 | |
| TG.18:1_34:4 | 870.755 | 571.472 | 144 | 35 | 10 | |
| TG.18:1_35:2 | 888.801 | 589.519 | 144 | 36 | 10 | |
| TG.18:1_35:3 | 886.786 | 587.503 | 144 | 36 | 10 | |
| TG.18:1_36:0 | 906.848 | 607.566 | 161 | 37 | 10 | |
| TG.18:1_36:1 | 904.833 | 605.55 | 161 | 37 | 10 | |
| TG.18:1_36:2 | 902.817 | 603.535 | 46 | 37 | 10 | |
| TG.18:1_36:3 | 900.801 | 601.519 | 161 | 37 | 10 | |
| TG.18:1_36:4 | 898.786 | 599.503 | 161 | 37 | 10 | |
| TG.18:1_36:5 | 896.77 | 597.488 | 161 | 37 | 10 | |
| TG.18:1_36:6 | 894.755 | 595.472 | 161 | 37 | 10 | |
| TG.18:1_38:5 | 924.801 | 625.519 | 161 | 39 | 10 | |
| TG.18:1_38:6 | 922.786 | 623.503 | 161 | 39 | 10 | |
| TG.18:1_38:7 | 920.77 | 621.488 | 161 | 39 | 10 | |
| TG.18:2_28:0 | 792.708 | 495.441 | 126 | 31 | 10 | |
| TG.18:2_30:0 | 820.739 | 523.472 | 131 | 33 | 10 | |
| TG.18:2_30:1 | 818.723 | 521.456 | 131 | 33 | 10 | |
| TG.18:2_31:0 | 834.755 | 537.488 | 131 | 34 | 10 | |
| TG.18:2_32:0 | 848.77 | 551.503 | 144 | 35 | 10 | |
| TG.18:2_32:1 | 846.755 | 549.488 | 144 | 35 | 10 | |
| TG.18:2_32:2 | 844.739 | 547.472 | 144 | 35 | 10 | |
| TG.18:2_33:0 | 862.786 | 565.519 | 144 | 35 | 10 | |
| TG.18:2_33:1 | 860.77 | 563.503 | 144 | 35 | 10 | |
| TG.18:2_33:2 | 858.755 | 561.488 | 144 | 35 | 10 | |
| TG.18:2_34:0 | 876.801 | 579.535 | 144 | 35 | 10 | |
| TG.18:2_34:1 | 874.786 | 577.519 | 144 | 35 | 10 | |
| TG.18:2_34:2 | 872.77 | 575.503 | 144 | 35 | 10 | |
| TG.18:2_34:3 | 870.755 | 573.488 | 144 | 35 | 10 | |
| TG.18:2_35:1 | 888.801 | 591.535 | 144 | 36 | 10 | |
| TG.18:2_35:2 | 886.786 | 589.519 | 144 | 36 | 10 | |
| TG.18:2_36:0 | 904.833 | 607.566 | 161 | 37 | 10 | |
| TG.18:2_36:1 | 902.817 | 605.55 | 161 | 37 | 10 | |
| TG.18:2_36:2 | 900.801 | 603.535 | 161 | 37 | 10 | |
| TG.18:2_36:3 | 898.786 | 601.519 | 161 | 37 | 10 | |
| TG.18:2_36:4 | 896.77 | 599.503 | 161 | 37 | 10 | |
| TG.18:2_36:5 | 894.755 | 597.488 | 161 | 37 | 10 | |
| TG.18:2_38:4 | 924.801 | 627.535 | 161 | 39 | 10 | |
| TG.18:3_30:0 | 818.723 | 523.472 | 131 | 33 | 10 | |
| TG.18:3_32:0 | 846.755 | 551.503 | 144 | 35 | 10 | |
| TG.18:3_32:1 | 844.739 | 549.488 | 144 | 35 | 10 | |
| TG.18:3_34:0 | 874.786 | 579.535 | 144 | 35 | 10 | |
| TG.18:3_34:1 | 872.77 | 577.519 | 144 | 35 | 10 | |
| TG.18:3_34:2 | 870.755 | 575.503 | 144 | 35 | 10 | |
| TG.18:3_36:1 | 900.801 | 605.55 | 161 | 37 | 10 | |
| TG.18:3_36:2 | 898.786 | 603.535 | 161 | 37 | 10 | |
| TG.18:3_36:3 | 896.77 | 601.519 | 161 | 37 | 10 | |
| TG.20:1_32:1 | 876.801 | 549.488 | 144 | 35 | 10 | |
| TG.20:1_32:2 | 874.786 | 547.472 | 144 | 35 | 10 | |
| TG.20:1_34:0 | 906.848 | 579.535 | 161 | 37 | 10 | |
| TG.20:1_34:1 | 904.833 | 577.519 | 161 | 37 | 10 | |
| TG.20:1_34:2 | 902.817 | 575.503 | 161 | 37 | 10 | |
| TG.20:3_32:0 | 874.786 | 551.503 | 144 | 35 | 10 | |
| TG.20:3_32:2 | 870.755 | 547.472 | 144 | 35 | 10 | |
| TG.20:3_34:0 | 902.817 | 579.535 | 161 | 37 | 10 | |
| TG.20:3_34:1 | 900.801 | 577.519 | 161 | 37 | 10 | |
| TG.20:3_34:2 | 898.786 | 575.503 | 161 | 37 | 10 | |
| TG.20:3_34:3 | 896.77 | 573.488 | 161 | 37 | 10 | |
| TG.20:3_36:3 | 924.801 | 601.519 | 161 | 39 | 10 | |
| TG.20:3_36:4 | 922.786 | 599.503 | 161 | 39 | 10 | |
| TG.20:4_30:0 | 844.739 | 523.472 | 144 | 35 | 10 | |
| TG.20:4_32:0 | 872.77 | 551.503 | 144 | 35 | 10 | |
| TG.20:4_32:1 | 870.755 | 549.488 | 144 | 35 | 10 | |
| TG.20:4_32:2 | 868.739 | 547.472 | 144 | 35 | 10 | |
| TG.20:4_34:0 | 900.801 | 579.535 | 161 | 37 | 10 | |
| TG.20:4_34:1 | 898.786 | 577.519 | 161 | 37 | 10 | |
| TG.20:4_34:2 | 896.77 | 575.503 | 161 | 37 | 10 | |
| TG.20:4_36:2 | 924.801 | 603.535 | 161 | 39 | 10 | |
| TG.20:4_36:4 | 920.77 | 599.503 | 161 | 39 | 10 | |
| TG.20:5_34:0 | 898.786 | 579.535 | 161 | 37 | 10 | |
| TG.20:5_34:1 | 896.77 | 577.519 | 161 | 37 | 10 | |
| TG.20:5_34:2 | 894.755 | 575.503 | 161 | 37 | 10 | |
| TG.20:5_36:2 | 922.786 | 603.535 | 161 | 39 | 10 | |
| TG.22:4_34:2 | 924.801 | 575.503 | 161 | 39 | 10 | |
| TG.22:5_32:0 | 898.786 | 551.503 | 161 | 37 | 10 | |
| TG.22:5_32:1 | 896.77 | 549.488 | 161 | 37 | 10 | |
| TG.22:5_34:1 | 924.801 | 577.519 | 161 | 39 | 10 | |
| TG.22:5_34:2 | 922.786 | 575.503 | 161 | 39 | 10 | |
| TG.22:6_32:0 | 896.77 | 551.503 | 161 | 37 | 10 | |
| TG.22:6_32:1 | 894.755 | 549.488 | 161 | 37 | 10 | |
| TG.22:6_34:1 | 922.786 | 577.519 | 161 | 39 | 10 | |
| TG.22:6_34:2 | 920.77 | 575.503 | 161 | 39 | 10 | |
| Uric acid | 167.1 | 124.1 | -40 | -20 | -6 | |
| p-Cresol sulfate | 187 | 80 | -95 | -32 | -13 | |
| 4-Ethylphenyl sulfate | 201 | 121 | -70 | -26 | -15 | |
| Propionic acid | 208 | 136.8 | -30 | -26 | -25 | |
| Butyric acid | 221.9 | 136.8 | -68 | -30 | -5 | |
| Lactic acid | 224 | 137 | -55 | -12 | -10 | |
| Valeric acid | 236 | 137 | -75 | -24 | -5 | |
| Dimethylglycine | 237.1 | 137.1 | -147 | -24 | -8 | |
| 2-Hydroxybutyric acid | 238 | 137 | -101 | -26 | -17 | |
| 2-Hydroxyisobutyric acid | 238 | 152 | -120 | -20 | -10 | |
| 3-Hydroxybutyric acid | 238 | 193.8 | -58 | -21 | -6 | |
| 3-Hydroxyisobutyric acid | 238.1 | 164.9 | -126 | -18 | -13 | |
| Glyceric acid | 240.1 | 137 | -130 | -25 | -17 | |
| Caproic acid | 250 | 137.1 | -75 | -25 | -5 | |
| Guanidoacetic acid | 251 | 209 | -90 | -20 | -10 | |
| N-Acetyl-glycine | 251.1 | 137 | -124 | -27 | -9 | |
| 2-Hydroxy-2-methylbutyric acid | 252 | 137 | -131 | -26 | -15 | |
| 2-Hydroxyisovaleric acid | 252 | 152 | -139 | -22 | -13 | |
| 3-Hydroxyisovaleric acid | 252.1 | 152 | -90 | -26 | -14 | |
| Benzoic acid | 256 | 150 | -60 | -26 | -9 | |
| 5-Oxoproline | 263 | 125 | -117 | -26 | -10 | |
| Pipecolic acid | 263.1 | 137 | -67 | -29 | -15 | |
| Guanidinopropionic acid | 265 | 223 | -100 | -20 | -15 | |
| N-Acetyl-alanine | 265.1 | 137 | -145 | -31 | -11 | |
| 2-Hydroxy-3-methylvaleric acid | 266 | 151.9 | -149 | -27 | -10 | |
| Phenylacetic acid | 270 | 152 | -144 | -22 | -7 | |
| Caprylic acid | 278.1 | 137.1 | -80 | -28 | -5 | |
| N-Acetyl-serine | 281.1 | 251 | -82 | -18 | -13 | |
| Xanthosine | 283.2 | 151 | -72 | -25 | -15 | |
| 4-Hydroxyphenylacetic acid | 286 | 150.1 | -80 | -27 | -7 | |
| N-Acetyl-proline | 291.1 | 249 | -101 | -24 | -12 | |
| Tiglylglycine | 291.1 | 152 | -150 | -22 | -11 | |
| N-Acetyl-valine | 293.1 | 137 | -169 | -32 | -15 | |
| N-Acetyl-leucine | 307.1 | 137 | -168 | -32 | -8 | |
| N-Acetyl-isoleucine | 307.2 | 136.9 | -165 | -32 | -8 | |
| N-Acetyl-asparagine | 308.2 | 155.2 | -150 | -20 | -9 | |
| 3-Indoleacetic acid | 308.9 | 152.1 | -100 | -28 | -16 | |
| Argininic acid | 309 | 152 | -60 | -30 | -15 | |
| Hippuric acid | 313 | 136.9 | -65 | -26 | -11 | |
| N1-Acetyl-lysine | 322 | 280 | -64 | -27 | -12 | |
| N1-Acetyl-lysine | 322 | 280 | -64 | -27 | -12 | |
| N-Acetyl-glutamine | 322.2 | 137.1 | -64 | -35 | -10 | |
| Kynurenic acid | 323 | 150 | -85 | -28 | -15 | |
| Indole-3-propionic acid | 323.2 | 194 | -181 | -23 | -12 | |
| 5-Hydroxyindoleacetic acid | 325 | 132.1 | -84 | -30 | -5 | |
| p-Hydroxyhippuric acid | 328.9 | 208.8 | -91 | -28 | -9 | |
| N-Acetyl-histidine | 331.2 | 194 | -155 | -20 | -10 | |
| Indolelactic acid | 339.1 | 137 | -145 | -29 | -10 | |
| N-Acetyl-Arginine | 350.1 | 137 | -200 | -40 | -11 | |
| Pyruvic acid | 357 | 136.8 | -80 | -33 | -5 | |
| N-Acetyl-tyrosine | 357.1 | 251 | -171 | -25 | -13 | |
| Oxalic acid | N/A | N/A | N/A | N/A | N/A | |
| Malonic acid | 373 | 178 | -160 | -23 | -15 | |
| alpha-Ketoisovaleric acid | 385 | 150.1 | -125 | -30 | -12 | |
| Fumaric acid | 385 | 234 | -78 | -20 | -9 | |
| Maleic acid | 385.1 | 234 | -202 | -25 | -13 | |
| Methylmalonic acid | 387 | 177.9 | -85 | -28 | -9 | |
| Succinic acid | 387.1 | 234 | -100 | -40 | -20 | |
| Phenylacetylglutamine | 398.1 | 127 | -250 | -52 | -15 | |
| 2-oxoisocaproic acid | 399.1 | 150 | -169 | -30 | -10 | |
| Ethylmalonic acid | 401 | 178 | -166 | -25 | -12 | |
| Glutaric acid | 401.1 | 247.9 | -80 | -26 | -10 | |
| Malic acid | 403 | 207.9 | -60 | -26 | -12 | |
| 2-Hydroxyglutaric acid | 417.1 | 137 | -108 | -30 | -8 | |
| 2,5-Furandicarboxylic acid | 425.1 | 150 | -202 | -30 | -11 | |
| 2-oxoadipic acid | 429 | 137 | -160 | -38 | -8 | |
| 3-Methyladipic acid | 429 | 178 | -200 | -34 | -15 | |
| 3-Deoxyglucosone | 431.1 | 136.9 | -153 | -33 | -10 | |
| Quinolinic acid | 436 | 283 | -100 | -40 | -15 | |
| N-Acetyl-aspartic acid | 444.1 | 291 | -188 | -22 | -21 | |
| 4-Hydroxyphenylpyruvic acid | 449.1 | 178 | -90 | -30 | -10 | |
| N-Acetyl-glutamic acid | 458.2 | 263 | -80 | -31 | -15 | |
| alpha-Ketoglutaric acid | 550.1 | 232.7 | -104 | -36 | -24 | |
| cis-aconitic acid | 578 | 424.8 | -124 | -22 | -11 | |
| Citric acid | 596.3 | 221.9 | -92 | -50 | -16 | |
| Acetoacetic acid | 371 | 137 | -73 | -30 | -5 | |
| Threonic acid | 270.1 | 152 | -155 | -33 | -15 | |

¹Metabolite abbreviations: LysoPC, lysophosphatidylcholine; SM, sphingomyelin; OH, hydroxyl; PC, phosphatidylcholine; aa, diacyl; ae, acyl-alkyl; C0, free carnitine; C2, acetylcarnitine; C3, propionylcarnitine; C4.1, butenylcarnitine; C4, butyrylcarnitine/isobutyrylcarnitine; C5, valerylcarnitine/isovalerylcarnitine; C4OH, hydroxybutyrylcarnitine; C5OH, hydroxyvalerylcarnitine; C5MDC, methylglutarylcarnitine; C7DC, pimelylcarnitine; C8, octanoylcarnitine; C9, nonanoylcarnitine; C10.2, decadienylcarnitine; C12DC, dodecanedioylcarnitine; C14, myristoylcarnitine; C16, palmitoylcarnitine; C16.1, palmitoleylcarnitine; C18, stearoylcarnitine; C18.1, oleoylcarnitine; C18.2, linoleylcarnitine; Cer, ceramide; CE, cholesteryl ester; Hex2Cer, dihexosylceramide; HexCer, hexosylceramide; TG, triacylglycerol. ²MRM: multiple reaction monitoring; ³Q1: precursor ion; ⁴Q3: product ion; ⁵DP: declustering potential; ⁶V: volts; ⁷CE: collision energy; ⁸CXP: collision cell exit potential.

Values were transcribed from Zhang et al. Supplementary Table S2 (Optimized MRM Transitions and Mass Spectrometer Parameters). The listed Q1→Q3 pairs represent the optimized MRM transitions used for targeted metabolite acquisition and quantification.

**Table S2.** **List of significant plasma metabolites identified by t-test, ROC AUC, PLS-DA VIP, Random Forest MDA, and volcano plot analyses** in replacement beef heifers **across four seaweed supplementation levels and through six pairwise group comparisons.**

| **Analysis method** | **Significant metabolites ^1^** |  | |  |
| --- | --- | --- | --- | --- |
| **ROC ^2^ AUC ^3^** |  |  | |  |
| 0.0% vs.0.5% | TG^3^.20:5_36:2^^4^, Methionine sulfoxide^, Hydroxyisovaleric acid^, N-Acetylglutamine^, Isoleucine^. | | |  |
| 0.0% vs.1.0% | TG.18:0_30:1, TG.18:0_36:4, PC ae C40.3, TG.16:0_38:1, PC aa C36.6, TG.18:3_32:0, TG.16:0_38:7, PC ae C40.4 | | |  |
| 0.0% vs.2.0% | Valine, TG.18:0_38:6, Asymmetric dimethylarginine, C2, Trimethylamine, Inosine | | |  |
| 0.5% vs.1.0% | Cytidine, Deoxycytidine, Methionine sulfoxide, TG.20:3_32:1, N-Acetyl Glutamine, Hydroxyglutaric acid, p-Cresol sulfate, Tiglylglycine, N-Acetyl Alanine | | |  |
| 0.5% vs.2.0% | Cytidine, TG.18:1_36:4, TG.18:1_36:3, Dimethylamine | | |  |
| 1.0% vs.2.0% | Argininic acid, Proline, TG.17:1_34:1^, TG.18:2_32:1^, TG.17:0_34:1^ | | |  |
| **PLS-DA ^4^ VIP ^5^** |  |  | |  |
|  |  |  | |  |
| 0.0% vs.0.5% | NS ^6^ |  | |  |
| 0.0% vs.1.0% | Serotonin, Acetyl.ornithine, Creatine, Leucine, Hydroxyindoleacetic.acid, Hex2Cer.d18.1.14.0., Succinic.acid, Cer.d18.2.24.1., Inosine, TG.18:3_34:2., PC aa C24.0, Glutamic.acid, C3OH, Hex2Cer.d18.1.24.0., Indole.3.propionic.acid | | |  |
| 0.0% vs.2.0% | Valine, TG.18:0_38:6, Asymmetric dimethylarginine, C2, Trimethylamine, Inosine, PC aa C38.4, TG.18:2_36:0, TG.18:3_34:0, PC aa C40.3, Leucine, Oxoisocaproic acid, PC aa C28.1, TG.18:2_34:4, PC ae C38.3 | | |  |
| 0.5% vs.1.0% | Cytidine, Deoxycytidine, Methionine sulfoxide, TG.20:3_32:1., N-Acetyl.Glutamine, 2-Hydroxyglutaric.acid, p.Cresol.sulfate, Tiglylglycine, N-Acetyl.Alanine, N.Acetyl.Proline, PC aa C32.3, Creatinine, Isoleucine, 3-Hydroxyisovaleric acid, TG.18:2_36:5 | | |  |
| 0.5% vs.2.0% | Cytidine, TG.18:1_36:4, TG.18:1_36:3, Dimethylamine, Allantoin, TG.18:1_36:2, Deoxycytidine, TG.18:3_36:4, TG.18:1_36:5, Hydroxyisovaleric acid, Hydroxy 2-methylbutyric acid, TG.18:2_36:2, TG.18:2_36:3, TG.20:5_36:2, Hydroxyisobutyric acid | | |  |
| 1.0% vs.2.0% | Argininic acid, Proline, TG.17:1_34:1, TG.18:2_32:1, TG.17:0_34:1, TG.18:1_26:0, TG.16:0_40:6, TG.16:1_36:1, TG.16:0_33:1, Allantoin, TG.14:0_36:3, Valine, TG.18:0_30:1, TG.16:0_35:1, TG.14:0_35:2 | | |  |
| **Random Forest MDA ^7^** |  |  | |  |
| 0.0% vs.0.5% | Isoleucine, TG.18:0_30:1, TG.18:0_36:4, TG.18:2_34:0, TG.22:5_32:0, TG.16:0_36:5, TG.16:0_40:6, N-Acetylproline, TG.18:0_38:6, TG.18:0_36:5, TG.18:1_38:5, TG.20:2_34:1, TG.18:1_33:0, TG.20:3_32:0, TG.18:0_30:0 | | |  |
| 0.0% vs.1.0% | Malic acid, Deoxyadenosine, Indolelactic acid, Xanthosine, Serotonin, TG.16:0_16:0, C3.1, Creatine, Hex2Cer d18.1/24.0, C3OH, Hydroxyisovaleric acid, Hydroxyphenyl acetic acid, Butyric acid/Isobutyric acid, TG.20:3_34:0, Uric acid | | |  |
| 0.0% vs.2.0% | PC aa C28.1, TG.18:0_38:6, CE.20.3, TG.22:5_32:0, TG.18:2_33:1, TG.14:0_36:4, Thymidine, TG.17:0_34:2, C2, LysoPC a C18.0, LysoPC a C18.2, Choline, Guanidinopropionic acid, TG.18:0_38:7, Inosine | | |  |
| 0.5% vs.1.0% | Deoxycytidine, Isoleucine, N-Acetyl Glycine, Oxalic acid, N-Acetyl Alanine, Cytidine, Hydroxyglutaric acid, N1-Acetyl Lysine, Uridine, Creatinine, Methionine sulfoxide, Guanidinopropionic acid, N-Acetyl Proline, Pipecolic acid, Leucine | | |  |
| 0.5% vs.2.0% | Cytidine, N-Acetyl.Tyrosine, Deoxycytidine, TG.18:2_35:2., alpha.Aminobutyric.acid, N-Acetyl.Glutamine, Allantoin, N-Acetyl.Leucine, 2-Hydroxyisobutyric.acid, PC ae C32.1, N-Acetylproline, TG.20:4_34:0., PC aa C32.3, CE.17.0., Threonic.acid | | |  |
| 1.0% vs.2.0% | TG.22:5_34:1, TG.16:0_36:6, Argininic acid, TG.18:2_31:0, N-Acetyllysine, Allantoin, TG.20:5_34:1, TG.16:1_36:1, TG.18:2_36:0, TG.18:1_26:0, Oxalic acid, TG.18:2_32:1, TG.18:2_33:1, TG.18:1_38:6, TG.20:5_36:3 | | |  |
| **T-test** |  |  | |  |
| 0.0% vs.0.5% | NS |  | |  |
| 0.0% vs.1.0% | NS |  | |  |
| 0.0% vs.2.0% | Valine, TG.18:0_38:6, Asymmetric dimethylarginine, C2, Trimethylamine, Inosine | | |  |
| 0.5% vs.1.0% | NS |  | |  |
| 0.5% vs.2.0% | Cytidine |  | |  |
| 1.0% vs.2.0% | Argininic.acid, Proline | | |  |
| **Volcano Plot** |  | |  | |
| 0.0% vs.0.5% | NS | |  | |
| 0.0% vs.1.0% | NS | |  | |
| 0.0% vs.2.0% | Valine, TG.18:0_38:6, Asymmetric dimethylarginine, C2, Trimethylamine, Inosine | | | |
| 0.5% vs.1.0% | NS | |  | |
| 0.5% vs.2.0% | NS | |  | |
| 1.0% vs.2.0% | Proline | |  | |
| ¹Metabolite abbreviations: TG, triacylglycerol; PC, phosphatidylcholine; ae, acyl-alkyl; aa, diacyl; C2, acetylcarnitine; C3OH, hydroxypropionylcarnitine; Hex2Cer, dihexosylceramide; Cer, ceramide; CE, cholesteryl ester; LysoPC, lysophosphatidylcholine; N1, nitrogen at position 1. ²ROC: receiver operating characteristic; ³AUC: area under the curve; ⁴PLS-DA: partial least squares-discriminant analysis; ⁵VIP: variable importance in projection; ⁶NS: not significant; ⁷MDA: mean decrease accuracy.    ROC analysis identified significant metabolites across the four seaweed supplementation levels. Metabolites with AUC ≥ 0.75 and/or p < 0.05 across the six pairwise seaweed-level comparisons were considered significant. Metabolites marked with ^ indicate a tendency toward significance (AUC ≥ 0.75, p < 0.10). PLS-DA identified the top 15 metabolites differentiating comparison groups based on VIP score > 1 and p < 0.05. Random Forest analysis identified the top 15 metabolites based on MDA scores, which measure feature importance by the decrease in model accuracy after permutation; MDA values were derived from 1,000 decision trees with out-of-bag validation. Student’s t-test identified significant plasma metabolites at p < 0.05 for each pairwise comparison. Volcano plot analysis identified significant metabolites based on fold change and statistical significance (p < 0.05). | |  | |  |

| **Table S3.** Classification of measurement records into low, moderate, and high methane emission groups based on methane mean values adjusted for confounding effects. | | | | |
| --- | --- | --- | --- | --- |
| **ID^1^** | **Methane mean (CH₄, g/day)** | **Adjusted CH₄ (g/day)** | **Emission groups** |  |
| 0.5%W1-1 | 206.45 | -35.39 | Low |  |
| 0.5%W1-2 | 229.25 | 0.86 | Moderate |  |
| 0.5%W1-3 | 229.54 | -2.69 | Moderate |  |
| 0.5%W1-4 | 249.61 | 20.26 | Moderate |  |
| 0.5%W2-1 | 243.43 | 8.31 | Moderate |  |
| 0.5%W2-2 | 272.36 | 33.40 | High |  |
| 0.5%W2-4 | 224.56 | -26.89 | Low |  |
| 0%W1-1 | 211.61 | -23.20 | Moderate |  |
| 0%W1-2 | 204.48 | -21.69 | Moderate |  |
| 0%W1-3 | 220.04 | -9.97 | Moderate |  |
| 0%W1-4 | 227.19 | -12.43 | Moderate |  |
| 0%W2-1 | 258.99 | 16.49 | Moderate |  |
| 0%W2-2 | 265.30 | 35.29 | High |  |
| 0%W2-3 | 239.36 | 4.55 | Moderate |  |
| 0%W2-4 | 289.34 | 41.07 | High |  |
| 1%W1-1 | 174.93 | -42.23 | Low |  |
| 1%W1-2 | 185.30 | -37.63 | Low |  |
| 1%W1-3 | 254.77 | 37.61 | High |  |
| 1%W1-4 | 226.04 | 2.15 | Moderate |  |
| 1%W2-1 | 192.96 | -36.69 | Low |  |
| 1%W2-2 | 238.65 | 7.08 | Moderate |  |
| 1%W2-4 | 241.41 | 12.72 | Moderate |  |
| 2%W1-1 | 204.95 | -9.93 | Moderate |  |
| 2%W1-2 | 178.71 | -7.35 | Moderate |  |
| 2%W1-3 | 166.26 | -39.01 | Low |  |
| 2%W1-4 | 205.34 | 0.07 | Moderate |  |
| 2%W2-1 | 223.43 | 29.69 | High |  |
| 2%W2-2 | 240.05 | 28.05 | High |  |
| 2%W2-3 | 227.32 | 14.36 | Moderate |  |
| 2%W2-4 | 236.68 | 13.15 | Moderate |  |

^1^ W1 and W2 refer to Week 1 and Week 2 methane measurement periods.

Animals were classified as low, moderate, or high methane emitters based on adjusted CH₄ values. Low and high emitters were defined as values more than 1 SD below or above the mean, respectively, whereas moderate emitters were defined as values within 1 SD of the mean. The full table is provided in the Supplementary Material.

The number of animal measurement records classified as Low, Moderate, and High within each seaweed treatment group was as follows: 0.0% seaweed, n = 0 Low, 6 Moderate, and 2 High; 0.5% seaweed, n = 2 Low, 4 Moderate, and 1 High; 1.0% seaweed, n = 3 Low, 3 Moderate, and 1 High; and 2.0% seaweed, n = 1 Low, 5 Moderate, and 2 High.

| **Table S4.** List of significant plasma metabolites identified by univariate (t-test and volcano plot) and multivariate (ROC AUC, PLS-DA VIP, and Random Forest MDA) analyses associated with methane emission groups (high, moderate, and low) in replacement heifers across three pairwise methane emission group comparisons. | |  |  |
| --- | --- | --- | --- |
| **Analysis method** | **Significant metabolites ^1^** | |  |
| **ROC ^2^ AUC ^3^** |  | |  |
| High vs.Low | PC aa C32.2^, Urea^, Ethylmalonic.acid^ | |  |
| High vs.Moderate | TG.20:2_32:1, Hex2Cer.d18.1.16.0, TG.18:2_36:4, TG.18:2_36:3, TG.14:0_32:2, TG.16:0_32:2, Caproic.acid, Propionic.acid, TG.18:2_36:5, TG.18:2_36:2, TG.18:1_36:6, Urea, TG.20:3_36:4, TG.18:2_34:4, TG.18:2_28.0 | |  |
| Low vs.Moderate | TG.18:3_36:4, HexCer.d18.1.24.1, TG.18:3_34:2, alpha-Ketoisovaleric acid, TG.18:2_36:2, TG.18:1_36:6, TG.18:1_36:1, TG.18:3_36:1, PC ae C40.5, TG.18:1_34:1, PC ae C38.6, TG.18:1_36:3, PC ae C38.2, PC ae C36.5, TG.18:3_36:2. | |  |
| **PLS-DA ^4^ VIP ^5^** |  | |  |
|  |  | |  |
| High vs.Low | Citric.acid, PC aa C32.2, Urea, Ethylmalonic.acid, LysoPC a C20.4, N1-Acetyllysine, N6.Acetyllysine, trans.4.Hydroxyproline, Phenylalanine, PC ae C32.2, LysoPC a C16.0, SM.C24.0, Serine, TG.18:2_34:4, SM.(OH).C22.1, TG.20:3_32:1 | |  |
| High vs.Moderate | TG.20:2_32:1, Hex2Cer.d18.1.16.0, TG.18:2_36:4, TG.18:2_36:3, TG.14:0_32:2, TG.16:0_32:2, Caproic.acid, Propionic.acid, TG.18:2_36:5, TG.18:2_36:2, TG.18:1_36:6, Urea, TG.20:3_36:4, TG.18:2_34:4, TG.18:2_28:0 | |  |
| Low vs.Moderate | TG.18:3_36:4, HexCer.d18.1.24.1, TG.18:3_34:2, alpha-Ketoisovaleric acid, TG.18:2_36:2, TG.18:1_36:6, TG.18:1_36:1, TG.18:3_36:1, PC ae C40.5, TG.18:1_34:1, PC ae C38.6, TG.18:1_36:3, PC ae C38.2, PC ae C36.5, TG.18:3_36:2 | |  |
| **Random Forest MDA ^6^** |  | |  |
| High vs.Low | TG.18:2_36:5, Urea, Ethylmalonic.acid, PC ae C32.2, TG.17:1_34:2, TG.16:0_36:4, TG.14:0_38:4, Caproic.acid, TG.16:1_33:1, TG.16:0_38:4, N1-Acetyllysine, N6.Acetyllysine, TG.18:3_30:0, TG.16:0_38:2, TG.18:2_32:0, PC aa C32.2 | |  |
| High vs.Moderate | TG.18:2_36:2, TG.18:2_36:3, Ethylmalonic.acid, TG.18:3_36:4, Hydroxyisobutyric.acid, PC aa C36.6, TG.18:1_36:5, TG.18:2_36:4, TG.20:1_34:0, Carnosine, TG.18:1_36:2, TG.18:1_36:4, Uric.acid, Glycine, PC aa C38.6 | |  |
| Low vs.Moderate | N-Acetylserine, Creatinine, PC aa C40.6, TG.18:2_36:2, TG.18:2_36:3, TG.18:3_34:2, X2.Hydroxyisobutyric.acid, alpha-Ketoisovaleric acid, HexCer.d18.1.24.1, TG.18:2_36:4, TG.18:1_36:3, 3.Indoleacetic.acid, CE.16.1, PC ae C38.6, LysoPC.a.C14.0 | |  |
| **T-test** |  | |  |
| High vs.Low | NS ^7^ | |  |
| High vs.Moderate | TG.20:2_32:1, Hex2Cer.d18.1.16.0, TG.18:2_36:4, TG.18:2_36:3, TG.14:0_32:2, TG.16:0_32:2, Caproic.acid, Propionic.acid, TG.18:2_36:5, TG.18:2_36:2, TG.18:1_36:6, Urea, TG.20:3_36:4, TG.18:2_34:4, TG.18:2_28:0 | |  |
| Low vs.Moderate | TG.18:3_36:4, HexCer.d18.1.24.1, TG.18:3_34:2, alpha-Ketoisovaleric acid, TG.18:2_36:2, TG.18:1_36:6, TG.18:1_36:1, TG.18:3_36:1, PC ae C40.5, TG.18:1_34:1, PC ae C38.6, TG.18:1_36:3, PC ae C38.2, PC ae C36.5, TG.18:3_36:2 | |  |
| **Volcano Plot** |  | |  |
| High vs.Low | NS | |  |
| High vs.Moderate | TG.20:2_32:1, Hex2Cer.d18.1.16.0, TG.18:2_36:4, TG.18:2_36:3, TG.14:0_32:2, TG.16:0_32:2, Caproic.acid, TG.18:2_36:5, TG.18:2_36:2, TG.18:1_36:6, Urea, TG.20:3_36:4, TG.18:2_34:4, TG.18:2_28:0, TG.20:0_32:3 | |  |
| Low vs.Moderate | TG.18:3_36:4, HexCer.d18.1.24.1, TG.18:3_34:2, alpha-Ketoisovaleric acid, TG.18:2_36:2, TG.18:1_36:6, TG.18:1_36:1, TG.18:3_36:1, TG.18:1_34:1, PC ae C38.6, TG.18:1_36:3, PC ae C38.2, PC ae C36.5, TG.18:3_36:2, TG.18:2_36:3 | |  |

¹Metabolite abbreviations: PC, phosphatidylcholine; aa, diacyl; TG, triacylglycerol; Hex2Cer, dihexosylceramide; HexCer, hexosylceramide; ae, acyl-alkyl; LysoPC, lysophosphatidylcholine; N1, nitrogen at position 1; N6, nitrogen at position 6; SM, sphingomyelin; OH, hydroxyl; CE, cholesteryl ester. ²ROC: receiver operating characteristic; ³AUC: area under the curve; ⁴PLS-DA: partial least squares-discriminant analysis; ⁵VIP: variable importance in projection; ^6^MDA: mean decrease accuracy

^7^NS: not significant.

Metabolites marked with ^ indicate a tendency (AUC ≥0.75, p < 0.10), metabolites that are not statistically significant, but show a trend toward significance.

| **Table S5.** Quantitative plasma metabolite concentrations (mmol/L; mean ± SD) in replacement heifers supplemented with 0.0%, 0.5%, 1.0%, or 2.0% Atlantic GRO® brown seaweed at Week 1 (W1) and Week 2 (W2). | | | | | | | | | | |  |  |  |
| --- | --- | --- | --- | --- | --- | --- | --- | --- | --- | --- | --- | --- | --- |
|  |  |  |  |  |  |  |  |  |  | |  |  |  |
| **Metabolites ^1^** | **HMDB ^2^ ID** | **0.0% W1 ^3^** | **0.0% W2 ^4^** | **0.5% W1** | **0.5% W2** | **1% W1** | **1% W2** | **2% W1** | **2% W2** | | |  | |
| 7-Methylguanine | HMDB0000897 | 0.13 ± 0.01 | 0.12 ± 0.01 | 0.12 ± 0.01 | 0.12 ± 0.01 | 0.1 ± 0.01 | 0.11 ± 0.01 | 0.11 ± 0.01 | 0.13 ± 0.01 |  | | |  |
| alpha-Aminobutyric acid | HMDB0000452 | 13.29 ± 0.96 | 13.9 ± 1.99 | 14.85 ± 1.2 | 20.15 ± 2.86 | 11.6 ± 0.72 | 14.17 ± 1.02 | 12.69 ± 3.09 | 17.96 ± 3.42 |  | | |  |
| Adenosine | HMDB0000050 | 0.12 ± 0.03 | 0.1 ± 0.03 | 0.07 ± 0.03 | 0.12 ± 0.01 | 0.1 ± 0.02 | 0.08 ± 0.03 | 0.1 ± 0.03 | 0.13 ± 0.01 |  | | |  |
| Asymmetric dimethylarginine | HMDB0001539 | 1.16 ± 0.19 | 0.97 ± 0.07 | 1.12 ± 0.04 | 1.11 ± 0.06 | 1.02 ± 0.08 | 1.05 ± 0.06 | 0.97 ± 0.14 | 1.05 ± 0.08 |  | | |  |
| Dimethylarginine | HMDB0003334 | 3.38 ± 0.37 | 3.24 ± 0.12 | 3.13 ± 0.11 | 3.27 ± 0.11 | 3.15 ± 0.19 | 3.11 ± 0.19 | 2.91 ± 0.32 | 3.4 ± 0.15 |  | | |  |
| Alanine | HMDB0000161 | 262.6 ± 31.86 | 317.52 ± 29.51 | 300.47 ± 25.76 | 363.25 363.25 ± 10.69 | 259.1 ± 11.51 | 267.65 ± 7.77 | 253.58 ± 33.06 | 312.62 ± 15.66 |  | | |  |
| Allantoin | HMDB0000462 | 41.12 ± 2.49 | 42.26 ± 0.85 | 39.16 ± 1.82 | 43.27 ± 1.37 | 37.2 ± 0.95 | 40.76 ± 0.44 | 36.14 ± 1.97 | 43.23 ± 2.47 |  | | |  |
| alpha-Aminoadipic acid | HMDB0000510 | 3.12 ± 0.45 | 2.4 ± 0.37 | 3.58 ± 0.24 | 3.1 ± 0.69 | 3.64 ± 0.47 | 3.16 ± 0.33 | 3.2 ± 0.82 | 3.14 ± 0.42 |  | | |  |
| Arginine | HMDB0000517 | 177.5 ± 17.96 | 167.9 ± 13.98 | 182.27 ± 4.61 | 177.08 ± 12.36 | 170.55 ± 11.91 | 177.02 ± 8.16 | 154.43 ± 20.41 | 158.8 ± 13.95 |  | | |  |
| Asparagine | HMDB0000168 | 25.93 ± 3.84 | 31.01 ± 2.86 | 28.2 ± 2.35 | 30.35 ± 1.42 | 23.56 ± 1.67 | 24.74 ± 1.76 | 19.33 ± 4.01 | 25.36 ± 1.65 |  | | |  |
| Aspartic acid | HMDB0000191 | 12.14 ± 1.29 | 15.9 ± 1.41 | 12.89 ± 2.28 | 15.68 ± 2.18 | 14.36 ± 2.11 | 12.79 ± 1.3 | 12.16 ± 3.45 | 10.24 ± 4.28 |  | | |  |
| beta-Alanine | HMDB0000056 | 0.47 ± 0.09 | 0.36 ± 0.05 | 0.4 ± 0.05 | 0.35 ± 0.07 | 0.37 ± 0.06 | 0.41 ± 0.08 | 0.31 ± 0.07 | 0.46 ± 0.08 |  | | |  |
| Betaine | HMDB0000043 | 154.01 ± 16.48 | 170.63 ± 33.05 | 141.35 ± 26.5 | 155.67 ± 25.36 | 138.76 ± 20.99 | 116.69 ± 9.82 | 118.83 ± 5.46 | 148.15 ± 17.73 |  | | |  |
| Carnosine | HMDB0000033 | 21.69 ± 3.47 | 20.51 ± 1.19 | 20.02 ± 1.19 | 28.05 ± 4.23 | 19.24 ± 0.71 | 21.66 ± 1.14 | 17.12 ± 1.11 | 21.32 ± 2.92 |  | | |  |
| Choline | HMDB0000097 | 22.43 ± 1.18 | 20.67 ± 2.49 | 21.08 ± 2.36 | 21.71 ± 1.6 | 19.87 ± 2.0 | 26.2 ± 4.41 | 25.31 ± 4.37 | 17.87 ± 3.42 |  | | |  |
| Citrulline | HMDB0000904 | 73.79 ± 8.65 | 72.72 ± 3.82 | 75.03 ± 8.01 | 83.09 ± 6.06 | 62.07 ± 2.61 | 66.3 ± 3.46 | 61.86 ± 8.94 | 68.58 ± 2.92 |  | | |  |
| Creatine | HMDB0000064 | 742.39 ± 395.55 | 295.3 ± 48.67 | 718.14 ± 401.92 | 342.15 ± 35.53 | 221.75 ± 17.25 | 287.02 ± 34.32 | 291.95 ± 17.84 | 293.48 ± 41.2 |  | | |  |
| Creatinine | HMDB0000562 | 183.58 ± 13.3 | 167.72 ± 11.6 | 166.26 ± 14.42 | 177.82 ± 14.29 | 182.65 ± 17.65 | 169.25 ± 14.23 | 156.28 ± 20.04 | 180.88 ± 12.94 |  | | |  |
| Cystathionine | HMDB0000099 | 1.14 ± 0.23 | 1.21 ± 0.2 | 0.87 ± 0.03 | 0.92 ± 0.06 | 0.77 ± 0.05 | 1.07 ± 0.13 | 0.75 ± 0.11 | 1.18 ± 0.22 |  | | |  |
| Cytidine | HMDB0000089 | 3.05 ± 0.36 | 2.72 ± 0.25 | 2.9 ± 0.31 | 3.19 ± 0.24 | 2.39 ± 0.3 | 2.46 ± 0.25 | 2.6 ± 0.39 | 3.13 ± 0.39 |  | | |  |
| Deoxycytidine | HMDB0000014 | 1.1 ± 0.4 | 1.13 ± 0.37 | 1.2 ± 0.21 | 1.48 ± 0.17 | 0.58 ± 0.13 | 0.87 ± 0.09 | 1.17 ± 0.25 | 1.78 ± 0.09 |  | | |  |
| Glutamine | HMDB0000641 | 408.68 ± 31.79 | 400.32 ± 33.86 | 413.36 ± 13.42 | 429.22 ± 31.77 | 418.8 ± 24.48 | 406.48 ± 37.3 | 349.88 ± 48.95 | 384.0 ± 30.11 |  | | |  |
| Glutamic acid | HMDB0000148 | 100.5 ± 7.03 | 97.66 ± 8.0 | 104.14 ± 9.0 | 108.71 ± 5.06 | 88.63 ± 4.97 | 109.12 ± 16.39 | 85.35 ± 15.81 | 87.76 ± 17.53 |  | | |  |
| Glycine | HMDB0000123 | 293.63 ± 24.17 | 309.18 ± 36.43 | 332.08 ± 12.6 | 349.05 ± 30.51 | 330.98 ± 37.54 | 276.62 ± 39.08 | 322.32 ± 33.15 | 356.1 ± 42.31 |  | | |  |
| Histidine | HMDB0000177 | 74.05 ± 6.08 | 78.0 ± 5.04 | 75.9 ± 7.19 | 89.09 ± 1.32 | 66.04 ± 2.95 | 75.86 ± 5.57 | 64.66 ± 8.29 | 76.99 ± 5.06 |  | | |  |
| Homoarginine | HMDB0000670 | 4.64 ± 0.62 | 5.12 ± 0.39 | 4.54 ± 0.64 | 5.48 ± 0.89 | 4.94 ± 0.37 | 5.26 ± 0.37 | 4.16 ± 0.55 | 5.22 ± 0.73 |  | | |  |
| Homocitrulline | HMDB0000679 | 1.0 ± 0.25 | 0.95 ± 0.09 | 0.87 ± 0.11 | 0.83 ± 0.12 | 0.79 ± 0.07 | 0.86 ± 0.04 | 0.82 ± 0.12 | 0.97 ± 0.13 |  | | |  |
| Isoleucine | HMDB0000172 | 125.03 ± 19.49 | 129.88 ± 10.26 | 146.36 ± 12.44 | 136.9 ± 10.99 | 117.5 ± 3.2 | 118.65 ± 7.66 | 116.69 ± 18.52 | 143.45 ± 11.58 |  | | |  |
| Indole | HMDB0000738 | 9.2 ± 1.55 | 9.8 ± 1.01 | 9.32 ± 1.23 | 10.52 ± 0.78 | 8.86 ± 0.58 | 9.18 ± 0.91 | 7.17 ± 1.48 | 8.93 ± 0.86 |  | | |  |
| Kynurenine | HMDB0000684 | 12.05 ± 0.85 | 11.45 ± 1.72 | 13.27 ± 2.04 | 12.13 ± 1.66 | 12.1 ± 0.44 | 11.36 ± 0.58 | 8.19 ± 0.29 | 11.37 ± 1.12 |  | | |  |
| Leucine | HMDB0000687 | 138.85 ± 29.37 | 143.2 ± 11.71 | 161.99 ± 14.22 | 154.88 ± 12.32 | 130.75 ± 2.41 | 132.72 ± 7.05 | 133.44 ± 25.84 | 160.8 ± 20.99 |  | | |  |
| Lysine | HMDB0000182 | 111.64 ± 13.71 | 123.15 ± 4.79 | 119.62 ± 8.98 | 128.25 ± 4.47 | 110.8 ± 2.84 | 113.38 ± 5.33 | 91.57 ± 13.29 | 115.64 ± 6.07 |  | | |  |
| Methionine | HMDB0000696 | 30.46 ± 5.5 | 33.3 ± 2.51 | 30.72 ± 2.27 | 37.38 ± 0.85 | 26.29 ± 0.53 | 30.38 ± 1.93 | 26.99 ± 5.1 | 33.1 ± 3.29 |  | | |  |
| Methionine sulfoxide | HMDB0002005 | 2.84 ± 0.38 | 3.32 ± 0.29 | 3.14 ± 0.3 | 3.14 ± 0.19 | 2.84 ± 0.14 | 2.92 ± 0.14 | 2.35 ± 0.27 | 2.95 ± 0.17 |  | | |  |
| Methylhistidine | HMDB0000479 | 28.42 ± 2.56 | 27.83 ± 1.29 | 28.17 ± 1.93 | 29.71 ± 2.92 | 26.99 ± 0.52 | 25.62 ± 0.77 | 22.52 ± 1.76 | 27.46 ± 2.68 |  | | |  |
| N2-Acetyl-ornithine | HMDB0003357 | 7.71 ± 2.36 | 7.46 ± 0.72 | 6.31 ± 0.72 | 6.89 ± 0.89 | 5.54 ± 0.2 | 5.74 ± 0.65 | 5.23 ± 0.35 | 7.46 ± 0.92 |  | | |  |
| Ornithine | HMDB0000214 | 62.84 ± 6.98 | 68.32 ± 4.6 | 71.84 ± 3.08 | 81.62 ± 4.29 | 60.9 ± 1.36 | 64.05 ± 4.7 | 51.61 ± 6.4 | 70.95 ± 3.87 |  | | |  |
| Phenylalanine | HMDB0000159 | 74.74 ± 6.31 | 75.91 ± 3.7 | 72.85 ± 2.71 | 75.37 ± 2.47 | 71.73 ± 2.14 | 69.74 ± 3.21 | 62.41 ± 11.23 | 75.32 ± 4.04 |  | | |  |
| Proline | HMDB0000162 | 99.5 ± 11.7 | 109.2 ± 4.99 | 105.32 ± 6.06 | 118.55 ± 4.32 | 99.56 ± 7.22 | 93.61 ± 5.06 | 93.24 ± 11.57 | 114.58 ± 5.96 |  | | |  |
| Sarcosine | HMDB0000271 | 1.45 ± 0.53 | 1.32 ± 0.23 | 0.8 ± 0.26 | 0.66 ± 0.15 | 0.8 ± 0.14 | 1.25 ± 0.5 | 1.0 ± 0.34 | 1.58 ± 0.3 |  | | |  |
| Serine | HMDB0000187 | 104.27 ± 10.44 | 101.3 ± 11.09 | 98.73 ± 6.47 | 112.88 ± 7.74 | 94.03 ± 8.0 | 101.12 ± 9.53 | 91.74 ± 10.06 | 98.66 ± 6.94 |  | | |  |
| Serotonin | HMDB0000259 | 4.69 ± 1.27 | 4.26 ± 1.49 | 8.09 ± 2.98 | 8.78 ± 4.41 | 5.96 ± 1.6 | 11.22 ± 1.83 | 4.18 ± 1.25 | 6.17 ± 2.09 |  | | |  |
| Taurine | HMDB0000251 | 39.37 ± 4.89 | 35.89 ± 2.95 | 50.8 ± 11.48 | 49.92 ± 7.77 | 35.39 ± 1.91 | 49.52 ± 6.62 | 47.03 ± 15.5 | 38.31 ± 7.27 |  | | |  |
| Threonine | HMDB0000167 | 91.57 ± 17.0 | 99.99 ± 8.11 | 93.98 ± 9.27 | 101.97 ± 11.36 | 86.22 ± 5.79 | 85.06 ± 3.21 | 74.32 ± 16.06 | 90.68 ± 11.65 |  | | |  |
| Thymidine | HMDB0000273 | 0.79 ± 0.12 | 0.86 ± 0.13 | 0.93 ± 0.05 | 0.96 ± 0.09 | 0.79 ± 0.09 | 0.83 ± 0.12 | 0.84 ± 0.1 | 0.94 ± 0.07 |  | | |  |
| Trimethylamine N-oxide | HMDB0000925 | 26.49 ± 17.41 | 21.38 ± 16.08 | 9.5 ± 1.78 | 8.53 ± 2.2 | 6.54 ± 2.46 | 47.22 ± 24.14 | 4.36 ± 1.09 | 6.28 ± 1.31 |  | | |  |
| trans-4-Hydroxyproline | HMDB0000725 | 29.86 ± 1.46 | 31.91 ± 4.55 | 28.49 ± 1.1 | 28.9 ± 2.57 | 31.25 ± 4.69 | 26.3 ± 2.64 | 29.6 ± 2.69 | 31.9 ± 2.31 |  | | |  |
| Tryptophan | HMDB0000929 | 53.22 ± 5.18 | 53.73 ± 3.76 | 53.64 ± 4.51 | 56.53 ± 3.12 | 50.79 ± 0.68 | 51.38 ± 2.13 | 45.24 ± 8.15 | 55.56 ± 4.23 |  | | |  |
| Tyrosine | HMDB0000158 | 66.18 ± 6.92 | 80.83 ± 6.35 | 70.09 ± 5.87 | 77.99 ± 1.14 | 59.85 ± 3.25 | 68.08 ± 4.38 | 56.98 ± 10.71 | 72.73 ± 2.61 |  | | |  |
| Uridine | HMDB0000296 | 4.06 ± 0.47 | 3.67 ± 0.44 | 4.36 ± 0.69 | 4.04 ± 0.42 | 3.05 ± 0.25 | 3.85 ± 0.58 | 3.62 ± 1.19 | 4.01 ± 0.74 |  | | |  |
| Valine | HMDB0000883 | 267.18 ± 23.81 | 273.35 ± 12.13 | 284.95 ± 20.53 | 295.0 ± 9.26 | 265.25 ± 4.76 | 276.23 ± 12.1 | 260.45 ± 33.99 | 291.0 ± 15.31 |  | | |  |
| Urea | HMDB0000294 | 12303.75 ± 2641.92 | 9686.0 ± 2240.48 | 9707.5 ± 387.74 | 9271.5 ± 778.43 | 9018.0 ± 386.51 | 11561.75 ± 1875.05 | 8321.75 ± 722.57 | 7386.25 ± 805.35 |  | | |  |
| Glucose | HMDB0000122 | 5982.1 ± 391.37 | 5159.56 ± 377.47 | 5950.23 ± 302.86 | 6041.54 ± 432.43 | 6309.22 ± 401.61 | 5201.75 ± 148.54 | 5072.02 ± 363.84 | 5398.14 ± 525.9 |  | | |  |
| LysoPC a C14.0 | HMDB0010379 | 0.9 ± 0.08 | 1.02 ± 0.16 | 0.83 ± 0.06 | 0.91 ± 0.1 | 0.75 ± 0.07 | 0.98 ± 0.06 | 0.68 ± 0.09 | 0.81 ± 0.07 |  | | |  |
| LysoPC a C16.1 | HMDB0010383 | 0.99 ± 0.08 | 1.1 ± 0.19 | 0.92 ± 0.03 | 1.08 ± 0.14 | 0.9 ± 0.12 | 1.22 ± 0.13 | 0.71 ± 0.08 | 0.92 ± 0.1 |  | | |  |
| LysoPC a C16.0 | HMDB0010382 | 13.38 ± 1.11 | 13.0 ± 1.58 | 12.06 ± 0.71 | 14.46 ± 1.96 | 13.36 ± 0.78 | 17.45 ± 1.3 | 8.67 ± 0.69 | 13.31 ± 1.76 |  | | |  |
| LysoPC a C17.0 | HMDB0012108 | 1.7 ± 0.14 | 1.63 ± 0.22 | 1.56 ± 0.12 | 1.83 ± 0.24 | 1.45 ± 0.16 | 2.02 ± 0.2 | 1.05 ± 0.14 | 1.58 ± 0.23 |  | | |  |
| LysoPC a C18.2 | HMDB0010386 | 38.13 ± 24.33 | 11.87 ± 1.27 | 37.22 ± 24.65 | 15.82 ± 1.97 | 12.5 ± 2.01 | 17.66 ± 2.32 | 8.44 ± 1.28 | 13.29 ± 1.87 |  | | |  |
| LysoPC a C18.1 | HMDB0002815 | 7.75 ± 0.71 | 7.69 ± 1.05 | 7.08 ± 0.37 | 8.61 ± 1.22 | 7.13 ± 1.0 | 10.42 ± 1.22 | 5.5 ± 0.47 | 7.97 ± 1.17 |  | | |  |
| LysoPC a C18.0 | HMDB0010384 | 11.79 ± 0.8 | 11.12 ± 1.87 | 10.5 ± 0.5 | 12.94 ± 2.16 | 10.41 ± 0.92 | 14.8 ± 1.2 | 7.44 ± 0.66 | 12.07 ± 2.33 |  | | |  |
| LysoPC a C20.4 | HMDB0010395 | 2.1 ± 0.06 | 2.12 ± 0.28 | 1.95 ± 0.18 | 2.55 ± 0.28 | 2.24 ± 0.27 | 3.07 ± 0.33 | 1.54 ± 0.22 | 2.13 ± 0.17 |  | | |  |
| LysoPC a C24.0 | HMDB0010405 | 0.08 ± 0.02 | 0.09 ± 0.02 | 0.12 ± 0.02 | 0.1 ± 0.02 | 0.09 ± 0.02 | 0.13 ± 0.02 | 0.07 ± 0.01 | 0.12 ± 0.02 |  | | |  |
| LysoPC a C28.1 | HMDB0029221 | 0.19 ± 0.03 | 0.24 ± 0.04 | 0.21 ± 0.02 | 0.27 ± 0.03 | 0.17 ± 0.03 | 0.38 ± 0.06 | 0.18 ± 0.04 | 0.27 ± 0.02 |  | | |  |
| LysoPC a C28.0 | HMDB0029206 | 0.19 ± 0.02 | 0.22 ± 0.03 | 0.25 ± 0.02 | 0.27 ± 0.04 | 0.19 ± 0.03 | 0.36 ± 0.08 | 0.16 ± 0.02 | 0.26 ± 0.03 |  | | |  |
| SM OH) C14.1 | HMDB0013462 | 13.23 ± 1.28 | 12.7 ± 2.6 | 12.64 ± 0.9 | 15.37 ± 2.55 | 12.99 ± 0.93 | 16.59 ± 1.75 | 11.67 ± 1.45 | 16.13 ± 1.56 |  | | |  |
| SM C16.1 | HMDB0240613 | 14.33 ± 1.55 | 13.69 ± 2.55 | 13.67 ± 0.99 | 16.83 ± 2.57 | 14.18 ± 1.36 | 18.54 ± 2.0 | 12.39 ± 1.28 | 16.64 ± 1.22 |  | | |  |
| SM C16.0 | HMDB0010169 | 102.65 ± 12.3 | 102.79 ± 20.2 | 95.75 ± 4.42 | 117.65 ± 18.2 | 101.74 ± 7.01 | 138.31 ± 16.33 | 89.97 ± 12.13 | 126.08 ± 9.9 |  | | |  |
| SM(OH) C16.1 | HMDB0013463 | 11.92 ± 1.69 | 11.79 ± 2.16 | 12.26 ± 1.07 | 15.06 ± 1.78 | 12.64 ± 0.97 | 16.87 ± 1.7 | 11.23 ± 1.86 | 15.13 ± 1.33 |  | | |  |
| SM C18.1 | HMDB0012100 | 13.65 ± 1.48 | 13.93 ± 2.47 | 15.57 ± 1.69 | 20.63 ± 2.21 | 15.07 ± 2.28 | 20.88 ± 2.02 | 12.6 ± 2.16 | 16.99 ± 1.78 |  | | |  |
| PC aa C32.2 | HMDB0007874 | 6.08 ± 0.3 | 5.01 ± 0.54 | 7.68 ± 0.82 | 7.26 ± 0.1 | 7.21 ± 0.99 | 7.05 ± 0.6 | 5.78 ± 0.71 | 6.06 ± 0.65 |  | | |  |
| SM C18.0 | HMDB0001348 | 16.93 ± 2.3 | 18.11 ± 3.84 | 16.46 ± 0.57 | 19.59 ± 2.0 | 17.37 ± 1.41 | 23.98 ± 2.45 | 14.62 ± 2.56 | 20.45 ± 1.72 |  | | |  |
| SM C20.2 | HMDB0013465 | 1.85 ± 0.18 | 1.98 ± 0.32 | 2.19 ± 0.25 | 2.88 ± 0.3 | 2.14 ± 0.35 | 2.97 ± 0.26 | 1.58 ± 0.27 | 2.17 ± 0.23 |  | | |  |
| PC ae C36.0 | HMDB0013406 | 6.61 ± 0.21 | 5.41 ± 0.69 | 6.91 ± 0.45 | 6.99 ± 0.7 | 6.96 ± 0.91 | 6.97 ± 0.42 | 5.58 ± 0.63 | 6.19 ± 0.59 |  | | |  |
| PC aa C36.6 | HMDB0008690 | 3.45 ± 0.23 | 2.85 ± 0.38 | 3.98 ± 0.33 | 4.25 ± 0.19 | 4.25 ± 0.5 | 4.19 ± 0.35 | 3.06 ± 0.44 | 3.27 ± 0.34 |  | | |  |
| PC aa C36.0 | HMDB0007886 | 18.49 ± 0.78 | 15.4 ± 1.96 | 19.23 ± 0.78 | 19.19 ± 3.02 | 18.94 ± 1.95 | 19.5 ± 1.39 | 15.39 ± 1.72 | 17.79 ± 2.13 |  | | |  |
| SM(OH) C22.2 | HMDB0013467 | 12.72 ± 1.26 | 13.55 ± 2.65 | 11.76 ± 0.56 | 14.16 ± 2.65 | 13.2 ± 0.99 | 16.6 ± 1.32 | 10.76 ± 1.38 | 14.7 ± 1.35 |  | | |  |
| SM(OH) C22.1 | HMDB0013466 | 13.02 ± 1.69 | 14.91 ± 3.05 | 12.19 ± 0.93 | 15.82 ± 3.21 | 13.97 ± 0.91 | 18.15 ± 2.09 | 12.17 ± 1.85 | 17.13 ± 1.56 |  | | |  |
| PC aa C38.6 | HMDB0007991 | 7.94 ± 0.39 | 6.74 ± 1.42 | 7.49 ± 0.68 | 7.07 ± 1.17 | 8.58 ± 0.73 | 7.91 ± 0.71 | 6.23 ± 0.25 | 6.89 ± 0.61 |  | | |  |
| PC aa C38.0 | HMDB0007893 | 2.9 ± 0.15 | 2.63 ± 0.3 | 3.12 ± 0.2 | 3.13 ± 0.37 | 3.25 ± 0.35 | 3.31 ± 0.25 | 2.72 ± 0.33 | 3.08 ± 0.4 |  | | |  |
| PC ae C40.6 | HMDB0013422 | 2.32 ± 0.04 | 2.1 ± 0.38 | 2.24 ± 0.1 | 2.26 ± 0.41 | 2.53 ± 0.23 | 2.39 ± 0.12 | 1.96 ± 0.17 | 2.14 ± 0.2 |  | | |  |
| SM(OH) C24.1 | HMDB0013469 | 1.84 ± 0.18 | 2.03 ± 0.35 | 1.83 ± 0.12 | 2.33 ± 0.34 | 1.97 ± 0.18 | 2.84 ± 0.33 | 1.77 ± 0.31 | 2.5 ± 0.23 |  | | |  |
| PC aa C40.6 | HMDB0008057 | 11.06 ± 0.67 | 9.57 ± 2.25 | 10.04 ± 1.16 | 9.58 ± 2.66 | 12.04 ± 1.27 | 10.43 ± 1.41 | 9.51 ± 1.11 | 10.23 ± 1.57 |  | | |  |
| PC aa C40.2 | HMDB0008688 | 1.05 ± 0.05 | 0.96 ± 0.06 | 1.18 ± 0.08 | 1.14 ± 0.11 | 1.3 ± 0.18 | 1.33 ± 0.09 | 0.94 ± 0.12 | 1.08 ± 0.14 |  | | |  |
| PC aa C40.1 | HMDB0007993 | 0.42 ± 0.01 | 0.37 ± 0.03 | 0.44 ± 0.04 | 0.44 ± 0.05 | 0.46 ± 0.05 | 0.5 ± 0.04 | 0.4 ± 0.04 | 0.45 ± 0.04 |  | | |  |
| C0 | HMDB0000062 | 22.64 ± 1.94 | 22.48 ± 1.61 | 24.9 ± 3.11 | 32.32 ± 2.24 | 22.27 ± 1.72 | 21.87 ± 0.73 | 19.67 ± 3.26 | 23.12 ± 2.21 |  | | |  |
| C2 | HMDB0000201 | 5.86 ± 0.56 | 5.3 ± 0.99 | 6.49 ± 0.91 | 6.51 ± 0.55 | 5.63 ± 0.42 | 7.73 ± 1.17 | 5.01 ± 0.56 | 5.75 ± 0.92 |  | | |  |
| C3 | HMDB0000824 | 0.91 ± 0.12 | 1.11 ± 0.44 | 0.88 ± 0.11 | 0.97 ± 0.06 | 0.76 ± 0.03 | 1.16 ± 0.24 | 0.53 ± 0.04 | 0.72 ± 0.07 |  | | |  |
| C4.1 | HMDB0013126 | 0.05  ± 0.0 | 0.07 ± 0.0 | 0.06 ± 0.0 | 0.06 ± 0.0 | 0.07 ± 0.0 | 0.06 ± 0.01 | 0.06 ± 0.01 | 0.06 ± 0.0 |  | | |  |
| C4 | HMDB0002013 | 0.29 ± 0.04 | 0.33 ± 0.07 | 0.27 ± 0.03 | 0.28 ± 0.01 | 0.26 ± 0.01 | 0.34 ± 0.05 | 0.2 ± 0.01 | 0.26 ± 0.02 |  | | |  |
| C5 | HMDB0013128 | 0.14 ± 0.02 | 0.15 ± 0.04 | 0.12 ± 0.01 | 0.13 ± 0.01 | 0.12 ± 0.01 | 0.15 ± 0.02 | 0.09 ± 0.01 | 0.11 ± 0.01 |  | | |  |
| C4OH | HMDB0013127 | 0.06 ± 0.0 | 0.05 ± 0.0 | 0.06 ± 0.0 | 0.06 ± 0.0 | 0.06 ± 0.0 | 0.06 ± 0.01 | 0.05 ± 0.0 | 0.05 ± 0.0 |  | | |  |
| C5OH | HMDB0013132 | 0.09 ± 0.0 | 0.09 ± 0.01 | 0.1 ± 0.01 | 0.1 ± 0.01 | 0.1 ± 0.01 | 0.09 ± 0.01 | 0.09 ± 0.01 | 0.09 ± 0.0 |  | | |  |
| C8 | HMDB0000791 | 0.11 ± 0.01 | 0.09 ± 0.01 | 0.14 ± 0.01 | 0.11 ± 0.01 | 0.13 ± 0.01 | 0.1 ± 0.02 | 0.11 ± 0.02 | 0.1 ± 0.0 |  | | |  |
| C5MDC | HMDB0000552 | 0.04 ± 0.0 | 0.05 ± 0.01 | 0.05 ± 0.01 | 0.06 ± 0.01 | 0.05 ± 0.01 | 0.07 ± 0.02 | 0.05 ± 0.0 | 0.06 ± 0.01 |  | | |  |
| C9 | HMDB0006320 | 0.04 ± 0.0 | 0.04 ± 0.0 | 0.04 ± 0.0 | 0.04 ± 0.0 | 0.04 ± 0.01 | 0.04 ± 0.01 | 0.03 ± 0.0 | 0.03 ± 0.0 |  | | |  |
| C7DC | HMDB0013328 | 0.11 ± 0.06 | 0.13 ± 0.09 | 0.06 ± 0.0 | 0.06 ± 0.01 | 0.06 ± 0.01 | 0.08 ± 0.01 | 0.05 ± 0.01 | 0.06 ± 0.01 |  | | |  |
| C10.2 | HMDB0241102 | 0.12 ± 0.0 | 0.11 ± 0.01 | 0.11 ± 0.01 | 0.14 ± 0.02 | 0.12 ± 0.01 | 0.12 ± 0.01 | 0.1 ± 0.0 | 0.1 ± 0.01 |  | | |  |
| C14 | HMDB0005066 | 0.06 ± 0.01 | 0.05 ± 0.01 | 0.06 ± 0.01 | 0.05 ± 0.01 | 0.06 ± 0.01 | 0.05 ± 0.0 | 0.05 ± 0.0 | 0.05 ± 0.0 |  | | |  |
| C12DC | HMDB0013327 | 0.03 ± 0.0 | 0.03 ± 0.0 | 0.04 ± 0.0 | 0.03 ± 0.0 | 0.03 ± 0.0 | 0.03 ± 0.0 | 0.03 ± 0.0 | 0.03 ± 0.0 |  | | |  |
| C16.1 | HMDB0013207 | 0.03 ± 0.0 | 0.03 ± 0.0 | 0.04 ± 0.0 | 0.03 ± 0.0 | 0.04 ± 0.0 | 0.03 ± 0.0 | 0.03 ± 0.0 | 0.03 ± 0.0 |  | | |  |
| C16 | HMDB0000222 | 0.08 ± 0.01 | 0.08 ± 0.01 | 0.1 ± 0.01 | 0.08 ± 0.01 | 0.1 ± 0.01 | 0.08 ± 0.01 | 0.09 ± 0.01 | 0.07 ± 0.0 |  | | |  |
| C18.2 | HMDB0006469 | 0.11 ± 0.01 | 0.09 ± 0.01 | 0.13 ± 0.0 | 0.09 ± 0.02 | 0.13 ± 0.0 | 0.08 ± 0.01 | 0.12 ± 0.02 | 0.07 ± 0.0 |  | | |  |
| C18.1 | HMDB0006464 | 0.13 ± 0.01 | 0.14 ± 0.03 | 0.13 ± 0.01 | 0.13 ± 0.01 | 0.14 ± 0.01 | 0.13 ± 0.02 | 0.13 ± 0.01 | 0.13 ± 0.01 |  | | |  |
| C18 | HMDB0000848 | 0.05 ± 0.01 | 0.04 ± 0.0 | 0.06 ± 0.01 | 0.05 ± 0.01 | 0.07 ± 0.01 | 0.05 ± 0.0 | 0.04 ± 0.0 | 0.05 ± 0.0 |  | | |  |
| PC aa C28.1 | HMDB0007867 | 1.94 ± 0.11 | 1.62 ± 0.15 | 2.24 ± 0.2 | 2.15 ± 0.15 | 2.15 ± 0.26 | 2.18 ± 0.19 | 1.91 ± 0.25 | 2.01 ± 0.23 |  | | |  |
| PC ae C30.2 | HMDB0013410 | 0.42 ± 0.03 | 0.35 ± 0.03 | 0.56 ± 0.05 | 0.52 ± 0.01 | 0.49 ± 0.09 | 0.46 ± 0.05 | 0.42 ± 0.04 | 0.44 ± 0.06 |  | | |  |
| PC ae C30.1 | HMDB0013402 | 5.63 ± 0.26 | 4.59 ± 0.48 | 6.25 ± 0.41 | 6.03 ± 0.45 | 6.27 ± 0.55 | 6.18 ± 0.41 | 5.11 ± 0.49 | 5.75 ± 0.52 |  | | |  |
| PC ae C30.0 | HMDB0013341 | 1.15 ± 0.08 | 0.99 ± 0.08 | 1.36 ± 0.08 | 1.29 ± 0.04 | 1.32 ± 0.14 | 1.3 ± 0.08 | 1.16 ± 0.15 | 1.25 ± 0.13 |  | | |  |
| PC aa C30.0 | HMDB0007869 | 4.18 ± 0.17 | 3.61 ± 0.38 | 4.5 ± 0.27 | 4.22 ± 0.36 | 4.3 ± 0.34 | 4.51 ± 0.29 | 3.83 ± 0.44 | 4.26 ± 0.46 |  | | |  |
| PC ae C32.2 | HMDB0013411 | 6.68 ± 0.41 | 5.2 ± 0.45 | 8.82 ± 1.01 | 8.17 ± 0.16 | 8.0 ± 1.05 | 7.87 ± 0.73 | 6.63 ± 0.85 | 6.89 ± 0.73 |  | | |  |
| PC ae C32.1 | HMDB0013404 | 8.38 ± 0.6 | 7.1 ± 0.87 | 9.07 ± 0.63 | 8.53 ± 0.53 | 9.43 ± 0.77 | 9.25 ± 0.49 | 7.54 ± 0.84 | 8.26 ± 0.82 |  | | |  |
| PC aa C32.3 | HMDB0007875 | 17.19 ± 0.36 | 14.91 ± 1.64 | 24.13 ± 3.87 | 25.8 ± 2.96 | 22.1 ± 4.45 | 23.47 ± 2.46 | 17.31 ± 2.7 | 18.66 ± 2.37 |  | | |  |
| PC aa C32.1 | HMDB0007872 | 14.34 ± 0.92 | 12.69 ± 1.8 | 15.18 ± 0.63 | 14.25 ± 1.59 | 15.13 ± 1.44 | 15.47 ± 0.89 | 12.53 ± 1.13 | 13.54 ± 1.18 |  | | |  |
| PC aa C32.0 | HMDB0000564 | 11.0 ± 0.64 | 9.41 ± 1.24 | 10.83 ± 0.62 | 10.08 ± 1.22 | 11.86 ± 1.07 | 12.21 ± 0.86 | 8.91 ± 0.79 | 10.11 ± 0.58 |  | | |  |
| PC ae C34.3 | HMDB0013413 | 24.79 ± 1.45 | 20.9 ± 2.04 | 30.85 ± 3.68 | 30.49 ± 2.14 | 30.2 ± 4.2 | 30.1 ± 2.29 | 20.5 ± 2.4 | 23.22 ± 2.11 |  | | |  |
| PC ae C34.2 | HMDB0011151 | 26.54 ± 0.99 | 22.57 ± 2.07 | 29.02 ± 1.88 | 27.57 ± 0.95 | 29.2 ± 3.19 | 28.39 ± 1.64 | 20.66 ± 2.53 | 24.42 ± 2.29 |  | | |  |
| PC ae C34.1 | HMDB0013412 | 22.92 ± 1.32 | 19.04 ± 2.34 | 23.13 ± 0.89 | 21.6 ± 2.58 | 22.9 ± 2.68 | 22.68 ± 1.13 | 17.85 ± 1.78 | 19.83 ± 2.04 |  | | |  |
| PC ae C34.0 | HMDB0013405 | 7.65 ± 0.48 | 6.66 ± 0.95 | 7.26 ± 0.32 | 6.89 ± 0.68 | 7.16 ± 0.75 | 7.55 ± 0.3 | 5.76 ± 0.71 | 6.61 ± 0.59 |  | | |  |
| PC aa C34.4 | HMDB0007883 | 6.47 ± 0.46 | 5.78 ± 0.68 | 8.15 ± 1.0 | 8.6 ± 0.5 | 8.06 ± 1.42 | 8.26 ± 0.8 | 5.44 ± 0.7 | 6.11 ± 0.7 |  | | |  |
| PC aa C34.3 | HMDB0007881 | 30.34 ± 0.6 | 25.42 ± 2.33 | 33.48 ± 1.96 | 31.86 ± 1.26 | 35.41 ± 4.18 | 35.05 ± 1.92 | 23.52 ± 3.47 | 28.8 ± 3.54 |  | | |  |
| PC aa C34.2 | HMDB0007880 | 190.22 ± 7.03 | 147.07 ± 8.42 | 198.9 ± 12.92 | 184.59 ± 10.71 | 212.14 ± 12.05 | 211.05 ± 14.94 | 136.54 ± 12.12 | 173.48 ± 17.75 |  | | |  |
| PC aa C34.1 | HMDB0007879 | 104.32 ± 5.33 | 82.89 ± 9.73 | 106.67 ± 2.28 | 98.44 ± 17.39 | 109.26 ± 7.99 | 111.12 ± 9.75 | 80.83 ± 5.93 | 92.68 ± 9.51 |  | | |  |
| PC ae C36.5 | HMDB0013415 | 9.46 ± 0.68 | 8.31 ± 1.04 | 10.88 ± 0.92 | 10.81 ± 0.51 | 11.98 ± 1.31 | 11.86 ± 0.74 | 8.02 ± 0.86 | 9.15 ± 0.77 |  | | |  |
| PC ae C36.4 | HMDB0013407 | 11.73 ± 0.92 | 9.86 ± 0.93 | 13.21 ± 1.18 | 12.74 ± 0.56 | 13.71 ± 1.8 | 13.52 ± 0.88 | 8.9 ± 0.86 | 10.46 ± 0.96 |  | | |  |
| PC ae C36.3 | HMDB0013425 | 16.93 ± 0.42 | 14.52 ± 1.45 | 18.17 ± 1.22 | 17.57 ± 0.89 | 18.82 ± 2.98 | 18.48 ± 1.18 | 12.59 ± 1.54 | 15.47 ± 1.68 |  | | |  |
| PC ae C36.2 | HMDB0013418 | 42.35 ± 1.58 | 34.53 ± 3.05 | 43.6 ± 3.15 | 43.13 ± 2.41 | 44.58 ± 6.1 | 44.88 ± 3.0 | 31.82 ± 3.88 | 38.6 ± 4.49 |  | | |  |
| PC ae C36.1 | HMDB0013414 | 28.33 ± 1.7 | 22.4 ± 2.79 | 29.5 ± 1.17 | 27.74 ± 4.36 | 28.37 ± 3.55 | 28.19 ± 1.64 | 22.44 ± 2.78 | 24.83 ± 3.23 |  | | |  |
| PC aa C36.5 | HMDB0007890 | 14.52 ± 0.83 | 11.57 ± 1.5 | 14.58 ± 1.08 | 13.89 ± 1.71 | 16.4 ± 1.3 | 16.33 ± 1.43 | 11.15 ± 0.86 | 13.13 ± 0.87 |  | | |  |
| PC aa C36.4 | HMDB0007889 | 45.74 ± 2.41 | 36.91 ± 4.14 | 45.04 ± 3.8 | 41.26 ± 5.24 | 51.54 ± 4.05 | 50.33 ± 3.49 | 32.94 ± 2.34 | 38.66 ± 3.02 |  | | |  |
| PC aa C36.3 | HMDB0007921 | 104.65 ± 0.94 | 84.85 ± 9.91 | 104.43 ± 5.74 | 96.53 ± 9.97 | 111.01 ± 12.97 | 107.64 ± 5.4 | 72.56 ± 8.91 | 89.69 ± 12.96 |  | | |  |
| PC aa C36.2 | HMDB0000593 | 313.76 ± 4.8 | 243.93 ± 25.45 | 316.88 ± 15.61 | 298.89 ± 23.85 | 322.35 ± 27.14 | 312.62 ± 17.07 | 219.81 ± 23.88 | 274.24 ± 39.48 |  | | |  |
| PC aa C36.1 | HMDB0007887 | 138.8 ± 6.65 | 108.82 ± 14.65 | 143.8 ± 2.82 | 134.42 ± 26.91 | 136.56 ± 11.69 | 137.26 ± 10.43 | 107.21 ± 11.22 | 125.16 ± 17.58 |  | | |  |
| PC ae C38.6 | HMDB0013409 | 5.46 ± 0.29 | 4.85 ± 0.61 | 5.94 ± 0.39 | 6.01 ± 0.51 | 6.44 ± 0.54 | 6.36 ± 0.44 | 4.71 ± 0.5 | 5.18 ± 0.49 |  | | |  |
| PC ae C38.5 | HMDB0013432 | 7.57 ± 0.42 | 6.41 ± 0.89 | 7.63 ± 0.52 | 7.39 ± 0.79 | 8.6 ± 0.76 | 8.31 ± 0.44 | 6.0 ± 0.51 | 6.76 ± 0.53 |  | | |  |
| PC ae C38.4 | HMDB0013420 | 10.6 ± 0.33 | 8.96 ± 1.28 | 10.43 ± 0.67 | 10.11 ± 1.25 | 11.65 ± 1.23 | 11.2 ± 0.55 | 7.94 ± 0.59 | 9.25 ± 0.78 |  | | |  |
| PC ae C38.3 | HMDB0013439 | 10.25 ± 0.58 | 8.53 ± 1.03 | 10.5 ± 0.51 | 9.93 ± 1.47 | 10.77 ± 1.47 | 10.22 ± 0.62 | 7.36 ± 0.8 | 8.42 ± 1.1 |  | | |  |
| PC ae C38.2 | HMDB0013431 | 10.05 ± 0.38 | 8.36 ± 0.93 | 10.5 ± 0.39 | 10.07 ± 1.48 | 10.98 ± 1.06 | 10.3 ± 0.68 | 8.56 ± 0.9 | 9.53 ± 1.04 |  | | |  |
| PC ae C38.1 | HMDB0013416 | 9.13 ± 0.55 | 7.59 ± 0.83 | 9.79 ± 0.61 | 9.13 ± 1.51 | 10.24 ± 0.78 | 9.76 ± 0.73 | 7.9 ± 0.77 | 9.04 ± 0.89 |  | | |  |
| PC ae C38.0 | HMDB0013408 | 2.25 ± 0.14 | 1.92 ± 0.25 | 2.53 ± 0.19 | 2.35 ± 0.16 | 2.44 ± 0.24 | 2.43 ± 0.18 | 1.92 ± 0.2 | 2.12 ± 0.17 |  | | |  |
| PC aa C38.5 | HMDB0007989 | 40.72 ± 4.13 | 31.27 ± 5.02 | 38.99 ± 5.16 | 33.84 ± 8.39 | 44.84 ± 4.43 | 39.31 ± 3.76 | 30.11 ± 2.31 | 33.23 ± 2.58 |  | | |  |
| PC aa C38.4 | HMDB0007988 | 83.71 ± 6.21 | 66.01 ± 9.13 | 79.35 ± 8.09 | 72.71 ± 14.36 | 90.75 ± 9.24 | 82.43 ± 6.53 | 60.36 ± 4.54 | 68.34 ± 5.44 |  | | |  |
| PC aa C38.3 | HMDB0008020 | 78.39 ± 9.36 | 59.88 ± 6.55 | 77.08 ± 6.63 | 68.08 ± 12.63 | 85.09 ± 9.71 | 75.05 ± 4.13 | 53.62 ± 5.08 | 60.24 ± 7.11 |  | | |  |
| SM C24.1 | HMDB0012107 | 49.55 ± 4.68 | 46.75 ± 7.44 | 45.19 ± 3.06 | 52.18 ± 11.1 | 50.26 ± 5.39 | 61.01 ± 5.14 | 36.09 ± 4.09 | 50.73 ± 5.43 |  | | |  |
| SM C24.0 | HMDB0011697 | 20.83 ± 2.19 | 21.36 ± 3.87 | 20.46 ± 1.01 | 24.79 ± 3.45 | 21.77 ± 1.97 | 28.85 ± 2.6 | 18.5 ± 2.91 | 25.94 ± 2.93 |  | | |  |
| PC aa C38.1 | HMDB0007894 | 10.29 ± 0.47 | 9.15 ± 1.13 | 11.53 ± 0.62 | 10.72 ± 1.2 | 11.82 ± 1.12 | 12.06 ± 0.88 | 9.72 ± 1.11 | 10.8 ± 1.2 |  | | |  |
| PC ae C40.5 | HMDB0013444 | 4.59 ± 0.42 | 3.75 ± 0.56 | 4.67 ± 0.44 | 4.26 ± 0.87 | 5.24 ± 0.51 | 4.54 ± 0.28 | 3.71 ± 0.35 | 3.95 ± 0.36 |  | | |  |
| PC ae C40.4 | HMDB0013442 | 3.17 ± 0.35 | 2.53 ± 0.34 | 3.43 ± 0.25 | 3.09 ± 0.56 | 3.79 ± 0.45 | 3.18 ± 0.16 | 2.57 ± 0.29 | 2.66 ± 0.23 |  | | |  |
| PC ae C40.3 | HMDB0013445 | 2.43 ± 0.3 | 1.82 ± 0.2 | 2.6 ± 0.24 | 2.21 ± 0.39 | 2.66 ± 0.3 | 2.24 ± 0.12 | 1.89 ± 0.23 | 1.99 ± 0.24 |  | | |  |
| PC ae C40.2 | HMDB0013437 | 1.5 ± 0.09 | 1.34 ± 0.15 | 1.7 ± 0.11 | 1.57 ± 0.17 | 1.79 ± 0.15 | 1.8 ± 0.12 | 1.44 ± 0.17 | 1.58 ± 0.18 |  | | |  |
| PC ae C40.1 | HMDB0013433 | 1.31 ± 0.04 | 1.17 ± 0.13 | 1.4 ± 0.09 | 1.31 ± 0.15 | 1.52 ± 0.15 | 1.55 ± 0.12 | 1.17 ± 0.13 | 1.34 ± 0.11 |  | | |  |
| PC aa C40.5 | HMDB0008055 | 33.86 ± 4.66 | 25.39 ± 3.38 | 32.88 ± 4.85 | 28.97 ± 7.86 | 39.23 ± 4.82 | 32.92 ± 3.16 | 26.1 ± 2.42 | 28.09 ± 2.64 |  | | |  |
| PC aa C40.4 | HMDB0008054 | 16.93 ± 2.61 | 13.22 ± 1.45 | 18.2 ± 2.34 | 16.48 ± 2.73 | 20.28 ± 2.13 | 18.8 ± 1.12 | 13.56 ± 1.78 | 14.62 ± 1.56 |  | | |  |
| PC aa C40.3 | HMDB0008086 | 6.05 ± 0.51 | 5.2 ± 0.6 | 6.9 ± 0.54 | 6.48 ± 0.82 | 7.52 ± 1.07 | 7.32 ± 0.51 | 5.05 ± 0.77 | 5.75 ± 0.8 |  | | |  |
| SM C26.1 | HMDB0013461 | 2.89 ± 0.29 | 3.09 ± 0.47 | 3.02 ± 0.18 | 3.78 ± 0.6 | 3.35 ± 0.48 | 4.4 ± 0.31 | 2.56 ± 0.46 | 3.56 ± 0.47 |  | | |  |
| SM C26.0 | HMDB0011698 | 0.65 ± 0.06 | 0.67 ± 0.1 | 0.66 ± 0.05 | 0.82 ± 0.14 | 0.65 ± 0.09 | 1.0 ± 0.09 | 0.62 ± 0.11 | 0.87 ± 0.08 |  | | |  |
| PC ae C42.5 | HMDB0013451 | 0.86 ± 0.13 | 0.68 ± 0.08 | 0.91 ± 0.11 | 0.84 ± 0.2 | 1.06 ± 0.14 | 0.84 ± 0.08 | 0.7 ± 0.07 | 0.76 ± 0.08 |  | | |  |
| PC ae C42.4 | HMDB0013448 | 0.54 ± 0.05 | 0.41 ± 0.05 | 0.58 ± 0.05 | 0.53 ± 0.08 | 0.63 ± 0.06 | 0.56 ± 0.04 | 0.43 ± 0.05 | 0.52 ± 0.06 |  | | |  |
| PC ae C42.3 | HMDB0013458 | 0.49 ± 0.03 | 0.41 ± 0.05 | 0.48 ± 0.03 | 0.48 ± 0.05 | 0.57 ± 0.07 | 0.53 ± 0.04 | 0.4 ± 0.06 | 0.46 ± 0.07 |  | | |  |
| PC ae C42.2 | HMDB0013438 | 0.44 ± 0.02 | 0.36 ± 0.05 | 0.43 ± 0.02 | 0.42 ± 0.07 | 0.48 ± 0.05 | 0.48 ± 0.04 | 0.35 ± 0.03 | 0.41 ± 0.04 |  | | |  |
| PC ae C42.1 | HMDB0013434 | 0.26 ± 0.01 | 0.25 ± 0.03 | 0.29 ± 0.02 | 0.27 ± 0.04 | 0.3 ± 0.04 | 0.3 ± 0.03 | 0.22 ± 0.02 | 0.25 ± 0.02 |  | | |  |
| PC ae C42.0 | HMDB0013423 | 0.17 ± 0.01 | 0.16 ± 0.03 | 0.17 ± 0.01 | 0.17 ± 0.02 | 0.21 ± 0.03 | 0.18 ± 0.01 | 0.15 ± 0.02 | 0.15 ± 0.02 |  | | |  |
| PC aa C42.6 | HMDB0008288 | 0.64 ± 0.04 | 0.59 ± 0.12 | 0.64 ± 0.06 | 0.65 ± 0.11 | 0.74 ± 0.07 | 0.73 ± 0.07 | 0.62 ± 0.07 | 0.7 ± 0.07 |  | | |  |
| PC aa C42.5 | HMDB0008257 | 1.4 ± 0.16 | 1.18 ± 0.17 | 1.5 ± 0.18 | 1.37 ± 0.2 | 1.6 ± 0.19 | 1.59 ± 0.1 | 1.21 ± 0.16 | 1.29 ± 0.17 |  | | |  |
| PC aa C42.4 | HMDB0008191 | 0.39 ± 0.03 | 0.36 ± 0.04 | 0.44 ± 0.02 | 0.44 ± 0.04 | 0.5 ± 0.08 | 0.51 ± 0.03 | 0.36 ± 0.06 | 0.41 ± 0.05 |  | | |  |
| PC aa C42.2 | HMDB0008092 | 0.19 ± 0.01 | 0.15 ± 0.02 | 0.19 ± 0.01 | 0.19 ± 0.03 | 0.2 ± 0.03 | 0.21 ± 0.02 | 0.14 ± 0.01 | 0.18 ± 0.03 |  | | |  |
| PC aa C42.1 | HMDB0008059 | 0.16 ± 0.01 | 0.15 ± 0.02 | 0.16 ± 0.01 | 0.15 ± 0.02 | 0.17 ± 0.01 | 0.17 ± 0.02 | 0.13 ± 0.01 | 0.15 ± 0.02 |  | | |  |
| PC aa C42.0 | HMDB0008058 | 0.14 ± 0.01 | 0.12 ± 0.01 | 0.14 ± 0.01 | 0.15 ± 0.02 | 0.16 ± 0.02 | 0.16 ± 0.01 | 0.1 ± 0.01 | 0.15 ± 0.02 |  | | |  |
| PC ae C44.6 | HMDB0013450 | 0.15 ± 0.01 | 0.12 ± 0.01 | 0.15 ± 0.01 | 0.15 ± 0.02 | 0.17 ± 0.01 | 0.16 ± 0.01 | 0.13 ± 0.0 | 0.15 ± 0.01 |  | | |  |
| PC ae C44.5 | HMDB0013456 | 0.16 ± 0.01 | 0.15 ± 0.01 | 0.17 ± 0.01 | 0.16 ± 0.02 | 0.19 ± 0.02 | 0.17 ± 0.01 | 0.13 ± 0.01 | 0.15 ± 0.01 |  | | |  |
| PC ae C44.4 | HMDB0013453 | 0.13 ± 0.01 | 0.1 ± 0.02 | 0.13 ± 0.01 | 0.13 ± 0.01 | 0.14 ± 0.01 | 0.14 ± 0.01 | 0.1 ± 0.01 | 0.12 ± 0.02 |  | | |  |
| PC ae C44.3 | HMDB0013449 | 0.09 ± 0.01 | 0.09 ± 0.01 | 0.11 ± 0.01 | 0.1 ± 0.01 | 0.1 ± 0.01 | 0.11 ± 0.01 | 0.08 ± 0.01 | 0.09 ± 0.01 |  | | |  |
| Cer(d18.1/16.0) | HMDB00790 | 0.14 ± 0.05 | 0.17 ± 0.04 | 0.17 ± 0.05 | 0.19 ± 0.05 | 0.23 ± 0.04 | 0.22 ± 0.05 | 0.18 ± 0.01 | 0.19 ± 0.03 |  | | |  |
| Cer(d18.1/22.0) | HMDB04952 | 0.08 ± 0.03 | 0.07 ± 0.01 | 0.08 ± 0.03 | 0.09 ± 0.01 | 0.09 ± 0.02 | 0.1 ± 0.03 | 0.07 ± 0.01 | 0.1 ± 0.02 |  | | |  |
| Cer(d18.1/23.0) | HMDB00950 | 0.13 ± 0.04 | 0.2 ± 0.04 | 0.16 ± 0.06 | 0.14 ± 0.02 | 0.2 ± 0.04 | 0.26 ± 0.05 | 0.15 ± 0.03 | 0.17 ± 0.02 |  | | |  |
| Cer(d18.1/24.0) | HMDB04956 | 0.16 ± 0.05 | 0.16 ± 0.02 | 0.19 ± 0.06 | 0.18 ± 0.05 | 0.21 ± 0.03 | 0.21 ± 0.06 | 0.16 ± 0.01 | 0.15 ± 0.02 |  | | |  |
| Cer(d18.1/24.1) | HMDB04953 | 0.1 ± 0.03 | 0.12 ± 0.03 | 0.12 ± 0.04 | 0.14 ± 0.01 | 0.12 ± 0.0 | 0.19 ± 0.03 | 0.11 ± 0.01 | 0.1 ± 0.01 |  | | |  |
| CE(14.0) | HMDB06725 | 21.8 ± 2.47 | 20.33 ± 1.15 | 20.92 ± 1.91 | 21.98 ± 1.48 | 17.59 ± 1.59 | 25.42 ± 3.84 | 20.48 ± 2.95 | 20.49 ± 2.83 |  | | |  |
| CE(14.1) | HMDB10367 | 1.12 ± 0.24 | 0.97 ± 0.17 | 1.19 ± 0.24 | 1.17 ± 0.29 | 0.81 ± 0.13 | 1.61 ± 0.4 | 1.21 ± 0.16 | 0.98 ± 0.18 |  | | |  |
| CE(15.0) | HMDB60057 | 65.7 ± 4.75 | 62.47 ± 4.93 | 64.51 ± 4.2 | 64.62 ± 4.89 | 55.28 ± 4.08 | 79.67 ± 10.97 | 61.47 ± 7.07 | 64.06 ± 8.37 |  | | |  |
| CE(15.1) | HMDB60056 | 3.96 ± 0.51 | 3.44 ± 0.31 | 3.3 ± 0.5 | 3.86 ± 0.63 | 2.68 ± 0.27 | 5.0 ± 1.45 | 3.31 ± 0.51 | 3.37 ± 0.7 |  | | |  |
| CE(16.0) | HMDB05188 | 83.33 ± 4.77 | 77.68 ± 7.23 | 82.64 ± 4.53 | 74.56 ± 5.89 | 76.78 ± 6.89 | 92.6 ± 7.49 | 73.63 ± 6.21 | 80.58 ± 7.36 |  | | |  |
| CE(16.1) | HMDB05197 | 85.24 ± 5.44 | 79.21 ± 7.18 | 84.81 ± 3.42 | 87.3 ± 14.92 | 71.08 ± 6.42 | 109.2 ± 19.77 | 81.59 ± 8.71 | 82.37 ± 10.29 |  | | |  |
| CE(17.0) | HMDB60059 | 9.13 ± 0.39 | 8.52 ± 0.96 | 8.96 ± 0.72 | 7.74 ± 0.74 | 8.23 ± 0.26 | 10.52 ± 1.46 | 7.29 ± 0.63 | 8.35 ± 0.76 |  | | |  |
| CE(17.1) | HMDB60060 | 64.66 ± 5.25 | 55.17 ± 4.03 | 66.27 ± 3.42 | 58.55 ± 5.62 | 57.22 ± 6.21 | 79.83 ± 13.29 | 52.6 ± 3.71 | 56.59 ± 5.8 |  | | |  |
| CE(18.0) | HMDB10368 | 4.74 ± 0.06 | 4.85 ± 0.57 | 5.12 ± 0.34 | 4.44 ± 0.76 | 4.79 ± 0.26 | 5.76 ± 0.67 | 3.84 ± 0.21 | 4.51 ± 0.62 |  | | |  |
| CE(18.1) | HMDB05191 | 125.43 ± 1.81 | 120.76 ± 13.28 | 122.09 ± 3.02 | 117.52 ± 16.15 | 113.51 ± 6.86 | 153.77 ± 23.87 | 110.46 ± 6.83 | 119.95 ± 12.0 |  | | |  |
| CE(18.2) | HMDB05192 | 1537.87 ± 70.8 | 1464.13 ± 115.57 | 1466.47 ± 90.13 | 1543.01 ± 144.36 | 1445.94 ± 49.76 | 2131.65 ± 411.72 | 1315.69 ± 77.53 | 1383.94 ± 111.01 |  | | |  |
| CE(18.3) | HMDB10369 | 584.1 ± 37.47 | 540.47 ± 49.67 | 544.23 ± 30.34 | 605.65 ± 87.99 | 527.7 ± 24.72 | 837.3 ± 173.46 | 525.53 ± 42.67 | 540.31 ± 66.12 |  | | |  |
| CE(20.0) | HMDB06740 | 1.44 ± 0.3 | 1.53 ± 0.17 | 1.25 ± 0.17 | 1.83 ± 0.85 | 1.16 ± 0.16 | 2.41 ± 1.04 | 1.09 ± 0.16 | 0.68 ± 0.14 |  | | |  |
| CE(20.1) | HMDB05194 | 0.79 ± 0.18 | 0.91 ± 0.09 | 0.81 ± 0.1 | 1.21 ± 0.5 | 0.56 ± 0.08 | 1.61 ± 0.5 | 0.68 ± 0.21 | 0.66 ± 0.04 |  | | |  |
| CE(20.3) | HMDB06736 | 31.36 ± 1.65 | 29.35 ± 2.51 | 26.86 ± 2.27 | 27.09 ± 3.82 | 28.17 ± 2.64 | 37.95 ± 6.56 | 22.25 ± 1.33 | 23.86 ± 2.3 |  | | |  |
| CE(20.4) | HMDB06726 | 179.45 ± 12.08 | 165.67 ± 21.04 | 154.2 ± 15.69 | 165.55 ± 32.02 | 162.46 ± 16.85 | 234.78 ± 45.75 | 139.45 ± 8.17 | 138.41 ± 7.87 |  | | |  |
| CE(20.5) | HMDB06731 | 125.69 ± 8.19 | 108.33 ± 17.43 | 100.03 ± 13.18 | 106.29 ± 29.47 | 108.38 ± 11.35 | 165.83 ± 35.87 | 96.97 ± 3.19 | 101.69 ± 7.24 |  | | |  |
| CE(22.5) | HMDB10374 | 4.38 ± 0.53 | 4.3 ± 0.65 | 3.59 ± 0.25 | 3.99 ± 0.81 | 4.02 ± 0.43 | 5.87 ± 1.24 | 3.43 ± 0.28 | 3.35 ± 0.23 |  | | |  |
| CE(22.6) | HMDB06733 | 7.66 ± 0.76 | 7.12 ± 1.43 | 5.5 ± 0.45 | 7.06 ± 1.73 | 6.0 ± 0.66 | 9.36 ± 2.02 | 6.23 ± 0.55 | 6.21 ± 0.71 |  | | |  |
| Hex2Cer(d18.1/16.0) | HMDB0006750 | 0.69 ± 0.05 | 0.53 ± 0.06 | 0.72 ± 0.04 | 0.59 ± 0.04 | 0.65 ± 0.03 | 0.71 ± 0.08 | 0.73 ± 0.09 | 0.73 ± 0.09 |  | | |  |
| Hex2Cer(d18.1/18.0) | HMDB0011591 | 0.06 ± 0.02 | 0.04 ± 0.01 | 0.09 ± 0.01 | 0.07 ± 0.01 | 0.09 ± 0.01 | 0.07 ± 0.01 | 0.06 ± 0.02 | 0.05 ± 0.01 |  | | |  |
| HexCer(d18.1/23.0) | HMDB0341524 | 0.09 ± 0.01 | 0.05 ± 0.02 | 0.12 ± 0.01 | 0.09 ± 0.01 | 0.12 ± 0.02 | 0.1 ± 0.02 | 0.09 ± 0.02 | 0.07 ± 0.02 |  | | |  |
| HexCer(d18.1/24.0) | HMDB0004978 | 0.4 ± 0.07 | 0.37 ± 0.04 | 0.52 ± 0.07 | 0.48 ± 0.24 | 0.46 ± 0.08 | 0.32 ± 0.04 | 0.36 ± 0.04 | 0.31 ± 0.11 |  | | |  |
| TG.14:0_32:2 | HMDB0042076 | 1.29 ± 0.67 | 1.61 ± 0.78 | 0.78 ± 0.16 | 1.56 ± 0.71 | 0.59 ± 0.12 | 2.61 ± 0.59 | 1.09 ± 0.41 | 0.62 ± 0.08 |  | | |  |
| TG.14:0_34:0 | HMDB0042065 | 3.4 ± 0.98 | 3.08 ± 0.67 | 2.32 ± 0.2 | 3.51 ± 1.25 | 1.73 ± 0.18 | 4.97 ± 1.53 | 1.95 ± 0.35 | 1.85 ± 0.29 |  | | |  |
| TG.14:0_34:1 | HMDB0042072 | 4.31 ± 1.7 | 3.12 ± 0.89 | 2.33 ± 0.15 | 5.81 ± 3.48 | 2.24 ± 0.3 | 7.06 ± 1.81 | 3.29 ± 1.03 | 2.22 ± 0.08 |  | | |  |
| TG.14:0_34:2 | HMDB0042078 | 2.1 ± 0.79 | 1.71 ± 0.69 | 1.23 ± 0.08 | 2.31 ± 1.33 | 1.18 ± 0.22 | 2.99 ± 0.86 | 1.57 ± 0.56 | 0.88 ± 0.19 |  | | |  |
| TG.14:0_35:1 | HMDB0042102 | 1.31 ± 0.47 | 1.04 ± 0.39 | 0.76 ± 0.12 | 1.55 ± 0.92 | 0.7 ± 0.14 | 1.66 ± 0.4 | 0.76 ± 0.16 | 0.72 ± 0.09 |  | | |  |
| TG.14:0_35:2 | HMDB0042108 | 0.91 ± 0.45 | 0.81 ± 0.24 | 0.52 ± 0.05 | 0.83 ± 0.41 | 0.34 ± 0.04 | 1.44 ± 0.59 | 0.49 ± 0.1 | 0.34 ± 0.03 |  | | |  |
| TG.14:0_36:1 | HMDB0042074 | 2.43 ± 1.0 | 1.87 ± 0.24 | 1.42 ± 0.13 | 2.41 ± 1.01 | 1.09 ± 0.13 | 3.17 ± 0.88 | 1.08 ± 0.07 | 1.31 ± 0.23 |  | | |  |
| TG.14:0_36:2 | HMDB0042081 | 1.76 ± 0.74 | 1.39 ± 0.28 | 0.92 ± 0.14 | 2.02 ± 1.03 | 0.84 ± 0.11 | 2.31 ± 0.66 | 1.09 ± 0.28 | 0.9 ± 0.1 |  | | |  |
| TG.14:0_36:3 | HMDB0042133 | 1.11 ± 0.5 | 0.78 ± 0.18 | 0.52 ± 0.02 | 1.18 ± 0.56 | 0.5 ± 0.04 | 1.63 ± 0.54 | 0.9 ± 0.45 | 0.55 ± 0.08 |  | | |  |
| TG.14:0_36:4 | HMDB0042082 | 0.3 ± 0.13 | 0.25 ± 0.11 | 0.2 ± 0.03 | 0.49 ± 0.24 | 0.17 ± 0.04 | 0.49 ± 0.02 | 0.32 ± 0.15 | 0.2 ± 0.05 |  | | |  |
| TG.14:0_38:5 | HMDB0042143 | 0.25 ± 0.05 | 0.16 ± 0.07 | 0.16 ± 0.07 | 0.32 ± 0.22 | 0.16 ± 0.06 | 0.43 ± 0.16 | 0.19 ± 0.1 | 0.16 ± 0.02 |  | | |  |
| TG.16:0_28:1 | HMDB0043834 | 1.55 ± 0.79 | 1.03 ± 0.27 | 0.8 ± 0.09 | 1.84 ± 0.97 | 0.61 ± 0.09 | 2.08 ± 0.6 | 1.16 ± 0.34 | 0.61 ± 0.09 |  | | |  |
| TG.16:0_32:0 | HMDB0005356 | 11.86 ± 3.47 | 9.28 ± 1.47 | 8.01 ± 0.85 | 11.04 ± 2.87 | 6.41 ± 0.67 | 17.31 ± 2.99 | 7.65 ± 1.31 | 6.61 ± 0.74 |  | | |  |
| TG.16:0_32:1 | HMDB0005359 | 11.06 ± 4.67 | 8.29 ± 2.06 | 6.3 ± 0.66 | 14.5 ± 8.6 | 5.38 ± 0.88 | 18.19 ± 4.04 | 8.5 ± 2.8 | 5.25 ± 0.49 |  | | |  |
| TG.16:0_32:2 | HMDB0005376 | 4.15 ± 1.49 | 4.68 ± 1.8 | 2.47 ± 0.4 | 4.94 ± 2.08 | 2.09 ± 0.36 | 8.24 ± 1.87 | 3.35 ± 1.19 | 2.45 ± 0.28 |  | | |  |
| TG.16:0_32:3 | HMDB0043843 | 0.76 ± 0.26 | 0.86 ± 0.21 | 0.49 ± 0.07 | 1.4 ± 0.68 | 0.52 ± 0.08 | 1.97 ± 0.52 | 0.81 ± 0.39 | 0.61 ± 0.04 |  | | |  |
| TG.16:0_33:1 | HMDB0043864 | 10.91 ± 4.22 | 8.13 ± 1.32 | 5.93 ± 0.5 | 12.16 ± 6.34 | 5.1 ± 0.7 | 14.84 ± 3.76 | 6.31 ± 1.21 | 5.34 ± 0.48 |  | | |  |
| TG.16:0_33:2 | HMDB0043870 | 6.89 ± 4.39 | 5.0 ± 1.41 | 2.19 ± 0.28 | 7.21 ± 3.68 | 1.92 ± 0.36 | 11.84 ± 4.53 | 3.63 ± 1.87 | 3.47 ± 0.17 |  | | |  |
| TG.16:0_34:0 | HMDB0005357 | 10.77 ± 1.93 | 8.79 ± 1.25 | 8.81 ± 0.65 | 11.13 ± 2.66 | 6.75 ± 0.85 | 13.91 ± 2.11 | 6.78 ± 0.94 | 7.05 ± 0.94 |  | | |  |
| TG.16:0_34:1 | HMDB0005360 | 20.86 ± 7.29 | 14.72 ± 2.9 | 13.23 ± 1.52 | 33.06 ± 13.79 | 12.31 ± 2.27 | 27.73 ± 4.28 | 15.23 ± 4.22 | 11.06 ± 1.07 |  | | |  |
| TG.16:0_34:2 | HMDB0005362 | 9.93 ± 3.35 | 7.29 ± 2.03 | 6.12 ± 0.85 | 14.94 ± 7.45 | 6.74 ± 1.4 | 14.83 ± 3.48 | 9.55 ± 4.04 | 5.65 ± 1.21 |  | | |  |
| TG.16:0_34:3 | HMDB0005379 | 3.43 ± 1.25 | 2.75 ± 0.63 | 1.94 ± 0.31 | 4.46 ± 2.16 | 2.51 ± 0.58 | 4.65 ± 0.91 | 3.42 ± 1.68 | 2.1 ± 0.48 |  | | |  |
| TG.16:0_34:4 | HMDB0043846 | 0.63 ± 0.23 | 0.63 ± 0.21 | 0.46 ± 0.08 | 0.89 ± 0.4 | 0.42 ± 0.13 | 1.16 ± 0.17 | 0.76 ± 0.32 | 0.46 ± 0.09 |  | | |  |
| TG.16:0_35:1 | HMDB0043866 | 6.14 ± 1.9 | 4.55 ± 0.58 | 3.9 ± 0.53 | 6.71 ± 2.96 | 3.14 ± 0.39 | 7.71 ± 1.82 | 3.42 ± 0.71 | 3.32 ± 0.39 |  | | |  |
| TG.16:0_35:2 | HMDB0043872 | 3.16 ± 1.04 | 2.32 ± 0.4 | 2.05 ± 0.27 | 4.02 ± 2.05 | 1.78 ± 0.29 | 4.09 ± 0.97 | 2.03 ± 0.58 | 1.74 ± 0.15 |  | | |  |
| TG.16:0_35:3 | HMDB0043867 | 1.29 ± 0.42 | 1.12 ± 0.23 | 0.87 ± 0.19 | 1.67 ± 0.68 | 0.86 ± 0.15 | 1.82 ± 0.33 | 0.96 ± 0.31 | 0.79 ± 0.08 |  | | |  |
| TG.16:0_36:2 | HMDB0005369 | 10.65 ± 3.3 | 7.64 ± 1.55 | 6.74 ± 0.65 | 17.47 ± 6.97 | 6.42 ± 1.15 | 15.49 ± 3.9 | 7.42 ± 1.96 | 6.11 ± 0.57 |  | | |  |
| TG.16:0_36:3 | HMDB0005384 | 6.89 ± 2.63 | 4.62 ± 0.88 | 4.03 ± 0.38 | 10.6 ± 4.11 | 4.18 ± 0.77 | 9.61 ± 2.48 | 5.31 ± 2.1 | 3.97 ± 0.59 |  | | |  |
| TG.16:0_36:4 | HMDB0005363 | 3.0 ± 0.92 | 2.4 ± 0.56 | 1.71 ± 0.26 | 4.93 ± 1.95 | 2.12 ± 0.42 | 5.73 ± 1.22 | 2.8 ± 1.38 | 1.98 ± 0.41 |  | | |  |
| TG.16:0_36:5 | HMDB0005380 | 0.96 ± 0.43 | 0.55 ± 0.15 | 0.5 ± 0.08 | 1.54 ± 0.57 | 0.57 ± 0.12 | 1.76 ± 0.34 | 0.95 ± 0.52 | 0.69 ± 0.21 |  | | |  |
| TG.16:0_36:6 | HMDB0043855 | 0.82 ± 0.36 | 0.64 ± 0.11 | 0.47 ± 0.03 | 0.92 ± 0.34 | 0.39 ± 0.04 | 1.51 ± 0.74 | 0.48 ± 0.11 | 0.46 ± 0.03 |  | | |  |
| TG.16:0_37:3 | HMDB0341521 | 0.79 ± 0.4 | 0.75 ± 0.19 | 0.38 ± 0.07 | 0.73 ± 0.23 | 0.38 ± 0.05 | 1.15 ± 0.37 | 0.41 ± 0.13 | 0.35 ± 0.03 |  | | |  |
| TG.16:0_38:1 | HMDB0005368 | 1.29 ± 0.47 | 1.03 ± 0.13 | 0.88 ± 0.22 | 1.98 ± 0.8 | 0.82 ± 0.08 | 2.16 ± 0.49 | 0.71 ± 0.12 | 1.0 ± 0.2 |  | | |  |
| TG.16:0_38:2 | HMDB0005383 | 0.87 ± 0.24 | 0.67 ± 0.17 | 0.63 ± 0.09 | 0.94 ± 0.31 | 0.63 ± 0.1 | 1.15 ± 0.19 | 0.5 ± 0.06 | 0.5 ± 0.07 |  | | |  |
| TG.16:0_38:3 | HMDB0005389 | 0.93 ± 0.32 | 0.77 ± 0.12 | 0.65 ± 0.14 | 1.03 ± 0.41 | 0.63 ± 0.14 | 1.33 ± 0.26 | 0.53 ± 0.13 | 0.48 ± 0.04 |  | | |  |
| TG.16:0_38:4 | HMDB0005370 | 1.2 ± 0.36 | 0.9 ± 0.2 | 0.73 ± 0.14 | 1.26 ± 0.69 | 0.73 ± 0.12 | 1.59 ± 0.41 | 0.92 ± 0.39 | 0.66 ± 0.06 |  | | |  |
| TG.16:0_38:5 | HMDB0005385 | 1.29 ± 0.28 | 0.87 ± 0.16 | 0.93 ± 0.16 | 1.73 ± 1.08 | 1.03 ± 0.24 | 2.29 ± 0.55 | 1.18 ± 0.45 | 0.73 ± 0.16 |  | | |  |
| TG.16:0_38:6 | HMDB0005391 | 0.58 ± 0.19 | 0.5 ± 0.07 | 0.37 ± 0.03 | 0.95 ± 0.55 | 0.51 ± 0.08 | 0.8 ± 0.21 | 0.63 ± 0.28 | 0.47 ± 0.06 |  | | |  |
| TG.16:0_38:7 | HMDB0044079 | 1.92 ± 0.79 | 1.65 ± 0.25 | 1.32 ± 0.4 | 2.25 ± 0.77 | 1.06 ± 0.16 | 3.0 ± 0.82 | 0.9 ± 0.17 | 1.08 ± 0.19 |  | | |  |
| TG.16:0_40:6 | HMDB0043939 | 0.73 ± 0.28 | 0.39 ± 0.07 | 0.31 ± 0.09 | 0.78 ± 0.51 | 0.43 ± 0.08 | 1.03 ± 0.36 | 0.43 ± 0.18 | 0.31 ± 0.05 |  | | |  |
| TG.16:0_40:7 | HMDB0044107 | 0.53 ± 0.25 | 0.36 ± 0.09 | 0.24 ± 0.02 | 0.49 ± 0.2 | 0.26 ± 0.04 | 0.52 ± 0.18 | 0.38 ± 0.19 | 0.25 ± 0.02 |  | | |  |
| TG.16:0_40:8 | HMDB0005392 | 0.45 ± 0.23 | 0.39 ± 0.1 | 0.21 ± 0.05 | 0.29 ± 0.07 | 0.25 ± 0.05 | 0.64 ± 0.21 | 0.28 ± 0.12 | 0.23 ± 0.03 |  | | |  |
| TG.16:1_28:0 | HMDB0042069 | 2.41 ± 1.45 | 1.32 ± 0.34 | 0.88 ± 0.11 | 2.08 ± 1.21 | 0.77 ± 0.08 | 3.31 ± 1.03 | 1.24 ± 0.24 | 0.7 ± 0.12 |  | | |  |
| TG.16:1_32:0 | HMDB0048434 | 6.8 ± 3.35 | 4.2 ± 0.94 | 3.38 ± 0.28 | 6.54 ± 3.72 | 2.76 ± 0.37 | 8.89 ± 2.15 | 3.96 ± 0.98 | 2.5 ± 0.22 |  | | |  |
| TG.16:1_32:1 | HMDB0010420 | 6.22 ± 2.79 | 4.88 ± 1.6 | 3.1 ± 0.31 | 7.22 ± 4.31 | 2.44 ± 0.36 | 9.69 ± 2.68 | 4.22 ± 0.88 | 2.4 ± 0.16 |  | | |  |
| TG.16:1_32:2 | HMDB0010422 | 6.91 ± 1.44 | 11.7 ± 4.21 | 6.42 ± 1.81 | 6.94 ± 1.07 | 5.23 ± 0.78 | 16.75 ± 4.75 | 5.81 ± 1.35 | 6.31 ± 0.76 |  | | |  |
| TG.16:1_33:1 | HMDB0048436 | 4.02 ± 2.05 | 2.76 ± 1.01 | 1.95 ± 0.18 | 4.21 ± 2.53 | 1.38 ± 0.21 | 5.75 ± 1.81 | 2.27 ± 0.61 | 1.46 ± 0.22 |  | | |  |
| TG.16:1_34:0 | HMDB0048478 | 4.67 ± 1.77 | 3.62 ± 0.64 | 2.72 ± 0.46 | 5.46 ± 2.86 | 2.07 ± 0.26 | 6.21 ± 1.65 | 2.74 ± 0.54 | 2.52 ± 0.41 |  | | |  |
| TG.16:1_34:1 | HMDB0010423 | 5.74 ± 2.47 | 4.28 ± 1.33 | 3.22 ± 0.23 | 8.35 ± 5.28 | 2.73 ± 0.52 | 7.13 ± 1.42 | 5.06 ± 1.7 | 2.72 ± 0.48 |  | | |  |
| TG.16:1_34:2 | HMDB0048464 | 2.91 ± 1.26 | 2.2 ± 0.79 | 1.54 ± 0.13 | 4.3 ± 2.62 | 1.44 ± 0.29 | 3.94 ± 1.03 | 2.47 ± 1.08 | 1.34 ± 0.27 |  | | |  |
| TG.16:1_34:3 | HMDB0048465 | 0.72 ± 0.24 | 0.61 ± 0.23 | 0.41 ± 0.09 | 1.05 ± 0.64 | 0.53 ± 0.1 | 1.35 ± 0.45 | 0.67 ± 0.31 | 0.39 ± 0.11 |  | | |  |
| TG.16:1_36:1 | HMDB0048460 | 2.03 ± 0.73 | 1.82 ± 0.36 | 1.26 ± 0.25 | 2.62 ± 1.24 | 1.08 ± 0.11 | 2.89 ± 0.8 | 1.3 ± 0.32 | 1.06 ± 0.06 |  | | |  |
| TG.16:1_36:2 | HMDB0005425 | 1.86 ± 0.7 | 1.52 ± 0.5 | 1.15 ± 0.14 | 3.19 ± 1.55 | 0.99 ± 0.13 | 2.64 ± 0.93 | 1.23 ± 0.47 | 0.95 ± 0.19 |  | | |  |
| TG.16:1_36:3 | HMDB0048487 | 0.86 ± 0.29 | 0.71 ± 0.22 | 0.61 ± 0.09 | 1.64 ± 0.81 | 0.51 ± 0.11 | 1.38 ± 0.42 | 0.83 ± 0.33 | 0.52 ± 0.08 |  | | |  |
| TG.16:1_36:4 | HMDB0048495 | 0.35 ± 0.14 | 0.27 ± 0.08 | 0.23 ± 0.04 | 0.66 ± 0.35 | 0.25 ± 0.05 | 0.65 ± 0.32 | 0.47 ± 0.3 | 0.3 ± 0.1 |  | | |  |
| TG.16:1_38:5 | HMDB0048622 | 0.13 ± 0.02 | 0.1 ± 0.01 | 0.12 ± 0.03 | 0.31 ± 0.14 | 0.13 ± 0.02 | 0.39 ± 0.18 | 0.28 ± 0.17 | 0.12 ± 0.03 |  | | |  |
| TG.17:0_32:1 | HMDB0011713 | 3.27 ± 1.34 | 2.93 ± 0.83 | 2.06 ± 0.22 | 3.39 ± 1.66 | 1.44 ± 0.26 | 5.45 ± 2.0 | 2.03 ± 0.56 | 1.37 ± 0.16 |  | | |  |
| TG.17:0_34:1 | HMDB0049113 | 5.08 ± 2.35 | 3.33 ± 0.74 | 2.66 ± 0.45 | 4.48 ± 1.99 | 2.18 ± 0.27 | 5.47 ± 1.53 | 2.54 ± 0.56 | 2.5 ± 0.45 |  | | |  |
| TG.17:0_34:2 | HMDB0011700 | 2.0 ± 0.81 | 1.55 ± 0.29 | 1.17 ± 0.16 | 2.09 ± 0.91 | 1.0 ± 0.15 | 2.75 ± 0.74 | 1.54 ± 0.57 | 1.12 ± 0.17 |  | | |  |
| TG.17:0_34:3 | HMDB0011701 | 0.75 ± 0.27 | 0.53 ± 0.12 | 0.5 ± 0.07 | 0.84 ± 0.31 | 0.52 ± 0.12 | 0.95 ± 0.29 | 0.68 ± 0.22 | 0.47 ± 0.05 |  | | |  |
| TG.17:0_36:3 | HMDB0050323 | 0.98 ± 0.38 | 0.75 ± 0.15 | 0.62 ± 0.07 | 1.04 ± 0.37 | 0.64 ± 0.13 | 1.18 ± 0.2 | 0.76 ± 0.36 | 0.53 ± 0.09 |  | | |  |
| TG.17:0_36:4 | HMDB0055545 | 0.37 ± 0.1 | 0.37 ± 0.09 | 0.28 ± 0.04 | 0.46 ± 0.14 | 0.27 ± 0.04 | 0.77 ± 0.22 | 0.31 ± 0.13 | 0.26 ± 0.06 |  | | |  |
| TG.17:1_32:1 | HMDB0042976 | 2.23 ± 1.06 | 1.79 ± 0.56 | 1.19 ± 0.17 | 2.68 ± 1.66 | 0.8 ± 0.07 | 3.39 ± 1.26 | 1.44 ± 0.36 | 0.83 ± 0.14 |  | | |  |
| TG.17:1_34:1 | HMDB0011700 | 3.44 ± 1.54 | 2.37 ± 0.45 | 1.78 ± 0.26 | 3.41 ± 1.81 | 1.42 ± 0.17 | 3.56 ± 0.73 | 1.72 ± 0.31 | 1.43 ± 0.16 |  | | |  |
| TG.17:1_34:2 | HMDB0011701 | 1.33 ± 0.42 | 1.31 ± 0.36 | 0.7 ± 0.06 | 1.51 ± 0.82 | 0.61 ± 0.08 | 1.98 ± 0.64 | 1.2 ± 0.49 | 0.64 ± 0.04 |  | | |  |
| TG.17:1_34:3 | HMDB0011702 | 0.46 ± 0.12 | 0.38 ± 0.09 | 0.29 ± 0.04 | 0.63 ± 0.29 | 0.36 ± 0.07 | 0.84 ± 0.27 | 0.32 ± 0.15 | 0.24 ± 0.03 |  | | |  |
| TG.17:1_36:3 | HMDB0055545 | 0.71 ± 0.37 | 0.55 ± 0.15 | 0.37 ± 0.04 | 0.62 ± 0.23 | 0.3 ± 0.07 | 0.64 ± 0.1 | 0.49 ± 0.23 | 0.3 ± 0.02 |  | | |  |
| TG.18:0_30:0 | HMDB0107858 | 6.79 ± 1.99 | 5.74 ± 0.69 | 4.54 ± 0.72 | 6.37 ± 1.39 | 3.41 ± 0.36 | 8.91 ± 2.59 | 3.31 ± 0.79 | 3.85 ± 0.83 |  | | |  |
| TG.18:0_30:1 | HMDB0010426 | 1.74 ± 0.66 | 1.2 ± 0.11 | 1.05 ± 0.15 | 2.07 ± 1.02 | 0.86 ± 0.06 | 2.4 ± 0.75 | 0.96 ± 0.23 | 0.88 ± 0.15 |  | | |  |
| TG.18:0_32:0 | HMDB0010427 | 9.81 ± 1.92 | 8.14 ± 1.2 | 7.87 ± 0.88 | 8.66 ± 1.38 | 5.84 ± 0.46 | 12.11 ± 2.05 | 4.88 ± 1.07 | 6.19 ± 1.31 |  | | |  |
| TG.18:0_32:1 | HMDB0010428 | 8.13 ± 2.74 | 6.46 ± 0.86 | 5.01 ± 0.76 | 8.6 ± 3.7 | 4.18 ± 0.46 | 10.27 ± 2.55 | 3.96 ± 0.46 | 4.4 ± 0.66 |  | | |  |
| TG.18:0_32:2 | HMDB0010430 | 2.09 ± 0.63 | 2.08 ± 0.35 | 1.57 ± 0.2 | 2.78 ± 1.02 | 1.31 ± 0.25 | 4.01 ± 1.19 | 1.32 ± 0.45 | 1.3 ± 0.09 |  | | |  |
| TG.18:0_34:2 | HMDB0044682 | 7.82 ± 2.74 | 6.25 ± 0.83 | 4.77 ± 0.9 | 9.83 ± 3.31 | 4.65 ± 0.79 | 10.59 ± 2.82 | 4.35 ± 1.2 | 4.91 ± 0.68 |  | | |  |
| TG.18:0_34:3 | HMDB0010432 | 3.62 ± 1.07 | 2.71 ± 0.3 | 2.38 ± 0.35 | 3.76 ± 1.14 | 2.41 ± 0.35 | 4.35 ± 1.42 | 2.11 ± 0.62 | 2.24 ± 0.17 |  | | |  |
| TG.18:0_36:1 | HMDB0010433 | 8.31 ± 2.21 | 7.45 ± 0.77 | 6.22 ± 1.42 | 12.23 ± 4.52 | 4.62 ± 0.65 | 11.6 ± 2.69 | 4.12 ± 0.81 | 5.04 ± 1.25 |  | | |  |
| TG.18:0_36:2 | HMDB0005397 | 8.07 ± 2.66 | 6.89 ± 1.07 | 5.31 ± 1.05 | 11.11 ± 3.62 | 4.53 ± 0.65 | 11.21 ± 2.38 | 3.85 ± 0.76 | 4.95 ± 1.02 |  | | |  |
| TG.18:0_36:3 | HMDB0005405 | 5.42 ± 1.7 | 3.94 ± 0.48 | 3.46 ± 0.63 | 6.66 ± 1.82 | 3.58 ± 0.48 | 6.86 ± 1.38 | 3.06 ± 0.97 | 3.37 ± 0.58 |  | | |  |
| TG.18:0_36:4 | HMDB0005411 | 2.72 ± 1.07 | 1.77 ± 0.28 | 1.44 ± 0.29 | 3.45 ± 1.06 | 1.43 ± 0.26 | 4.03 ± 0.98 | 1.8 ± 0.81 | 1.63 ± 0.2 |  | | |  |
| TG.18:0_36:5 | HMDB0044687 | 0.91 ± 0.4 | 0.65 ± 0.05 | 0.5 ± 0.06 | 1.32 ± 0.41 | 0.55 ± 0.07 | 1.33 ± 0.37 | 0.6 ± 0.24 | 0.46 ± 0.1 |  | | |  |
| TG.18:0_38:6 | HMDB0005412 | 0.9 ± 0.43 | 0.69 ± 0.16 | 0.42 ± 0.05 | 0.67 ± 0.27 | 0.44 ± 0.05 | 0.8 ± 0.29 | 0.41 ± 0.11 | 0.42 ± 0.06 |  | | |  |
| TG.18:0_38:7 | HMDB0044909 | 1.55 ± 0.61 | 1.38 ± 0.19 | 1.02 ± 0.33 | 1.23 ± 0.27 | 0.72 ± 0.1 | 1.9 ± 0.47 | 0.55 ± 0.15 | 0.97 ± 0.3 |  | | |  |
| TG.18:1_26:0 | HMDB0042069 | 0.94 ± 0.6 | 0.67 ± 0.08 | 0.5 ± 0.1 | 1.11 ± 0.39 | 0.31 ± 0.01 | 1.32 ± 0.31 | 0.59 ± 0.16 | 0.33 ± 0.07 |  | | |  |
| TG.18:1_28:1 | HMDB0042076 | 1.06 ± 0.68 | 0.69 ± 0.21 | 0.38 ± 0.1 | 1.01 ± 0.42 | 0.43 ± 0.12 | 1.43 ± 0.3 | 0.6 ± 0.19 | 0.29 ± 0.07 |  | | |  |
| TG.18:1_30:0 | HMDB0044726 | 7.31 ± 3.61 | 5.17 ± 1.22 | 3.6 ± 0.46 | 8.11 ± 3.89 | 2.91 ± 0.42 | 9.72 ± 2.46 | 4.14 ± 0.94 | 3.28 ± 0.49 |  | | |  |
| TG.18:1_30:1 | HMDB0005376 | 2.07 ± 0.95 | 1.62 ± 0.47 | 1.03 ± 0.11 | 2.83 ± 1.61 | 0.84 ± 0.11 | 3.31 ± 1.0 | 1.46 ± 0.48 | 0.83 ± 0.07 |  | | |  |
| TG.18:1_30:2 | HMDB0042543 | 0.54 ± 0.35 | 0.37 ± 0.16 | 0.34 ± 0.09 | 0.47 ± 0.26 | 0.27 ± 0.05 | 0.79 ± 0.33 | 0.31 ± 0.13 | 0.2 ± 0.03 |  | | |  |
| TG.18:1_31:0 | HMDB0011713 | 9.23 ± 4.16 | 7.25 ± 1.28 | 5.07 ± 0.88 | 10.76 ± 5.24 | 4.17 ± 0.47 | 12.19 ± 3.3 | 5.16 ± 1.19 | 4.38 ± 0.47 |  | | |  |
| TG.18:1_32:0 | HMDB0049092 | 14.89 ± 5.34 | 11.2 ± 2.03 | 9.29 ± 0.92 | 20.82 ± 8.04 | 8.24 ± 1.0 | 18.57 ± 2.87 | 10.11 ± 2.61 | 8.26 ± 0.84 |  | | |  |
| TG.18:1_32:1 | HMDB0010434 | 7.95 ± 3.25 | 6.02 ± 1.55 | 4.37 ± 0.55 | 10.48 ± 5.62 | 4.16 ± 0.73 | 10.68 ± 2.4 | 6.47 ± 2.25 | 3.94 ± 0.49 |  | | |  |
| TG.18:1_32:2 | HMDB0010436 | 2.29 ± 1.0 | 1.85 ± 0.48 | 1.4 ± 0.2 | 3.07 ± 1.35 | 1.18 ± 0.22 | 3.66 ± 0.91 | 1.73 ± 0.6 | 1.57 ± 0.19 |  | | |  |
| TG.18:1_32:3 | HMDB0049079 | 0.49 ± 0.22 | 0.33 ± 0.09 | 0.28 ± 0.02 | 0.64 ± 0.35 | 0.2 ± 0.03 | 0.54 ± 0.07 | 0.46 ± 0.2 | 0.27 ± 0.04 |  | | |  |
| TG.18:1_33:0 | HMDB0049113 | 10.31 ± 3.17 | 7.96 ± 0.89 | 6.85 ± 1.08 | 10.65 ± 4.34 | 5.28 ± 0.52 | 13.61 ± 3.71 | 5.04 ± 0.84 | 5.43 ± 0.82 |  | | |  |
| TG.18:1_33:1 | HMDB0049094 | 7.24 ± 2.54 | 5.17 ± 1.12 | 4.19 ± 0.48 | 8.27 ± 4.19 | 3.46 ± 0.47 | 8.96 ± 2.81 | 4.34 ± 1.34 | 3.39 ± 0.26 |  | | |  |
| TG.18:1_33:2 | HMDB0049099 | 1.93 ± 0.88 | 1.31 ± 0.31 | 0.97 ± 0.14 | 2.0 ± 0.9 | 0.83 ± 0.15 | 2.33 ± 0.69 | 1.37 ± 0.51 | 1.0 ± 0.08 |  | | |  |
| TG.18:1_34:1 | HMDB0010437 | 16.97 ± 5.27 | 12.29 ± 2.73 | 10.91 ± 1.08 | 31.36 ± 13.13 | 9.77 ± 1.64 | 23.94 ± 5.87 | 12.38 ± 3.79 | 9.46 ± 1.21 |  | | |  |
| TG.18:1_34:2 | HMDB0010439 | 6.43 ± 2.39 | 4.93 ± 1.31 | 4.29 ± 0.51 | 12.58 ± 5.32 | 3.99 ± 0.73 | 10.35 ± 2.75 | 5.99 ± 2.75 | 3.87 ± 0.8 |  | | |  |
| TG.18:1_34:3 | HMDB0049121 | 2.25 ± 0.65 | 1.66 ± 0.53 | 1.55 ± 0.27 | 4.67 ± 1.99 | 1.48 ± 0.26 | 3.37 ± 0.68 | 2.42 ± 1.22 | 1.64 ± 0.24 |  | | |  |
| TG.18:1_34:4 | HMDB0049129 | 0.47 ± 0.13 | 0.52 ± 0.13 | 0.31 ± 0.05 | 0.87 ± 0.3 | 0.33 ± 0.08 | 0.87 ± 0.26 | 0.56 ± 0.28 | 0.32 ± 0.08 |  | | |  |
| TG.18:1_35:2 | HMDB0049101 | 1.72 ± 0.67 | 1.5 ± 0.36 | 1.05 ± 0.18 | 2.28 ± 0.97 | 0.76 ± 0.1 | 2.03 ± 0.68 | 1.14 ± 0.37 | 0.91 ± 0.17 |  | | |  |
| TG.18:1_35:3 | HMDB0049096 | 0.75 ± 0.35 | 0.47 ± 0.07 | 0.4 ± 0.04 | 0.84 ± 0.4 | 0.39 ± 0.09 | 1.14 ± 0.56 | 0.53 ± 0.24 | 0.46 ± 0.06 |  | | |  |
| TG.18:1_36:0 | HMDB0049155 | 6.16 ± 2.01 | 5.07 ± 0.58 | 4.48 ± 0.81 | 7.71 ± 2.17 | 3.32 ± 0.45 | 7.83 ± 1.34 | 2.9 ± 0.52 | 3.54 ± 0.78 |  | | |  |
| TG.18:1_36:1 | HMDB0049116 | 8.39 ± 3.27 | 6.85 ± 1.61 | 4.8 ± 0.64 | 14.67 ± 6.31 | 3.93 ± 0.57 | 11.08 ± 2.49 | 4.15 ± 0.97 | 4.35 ± 0.88 |  | | |  |
| TG.18:1_36:2 | HMDB0010447 | 8.43 ± 4.64 | 4.93 ± 1.21 | 3.97 ± 0.55 | 38.98 ± 29.87 | 3.6 ± 0.72 | 16.33 ± 4.91 | 5.05 ± 2.08 | 3.29 ± 0.63 |  | | |  |
| TG.18:1_36:3 | HMDB0049142 | 5.52 ± 2.65 | 3.93 ± 1.12 | 3.04 ± 0.21 | 26.57 ± 20.3 | 2.87 ± 0.49 | 12.06 ± 3.5 | 3.94 ± 1.6 | 2.45 ± 0.23 |  | | |  |
| TG.18:1_36:4 | HMDB0049150 | 2.68 ± 1.23 | 2.04 ± 0.72 | 1.6 ± 0.14 | 17.78 ± 14.05 | 1.5 ± 0.21 | 7.44 ± 2.59 | 2.31 ± 1.0 | 1.29 ± 0.21 |  | | |  |
| TG.18:1_36:5 | HMDB0049276 | 0.65 ± 0.25 | 0.53 ± 0.17 | 0.33 ± 0.06 | 4.26 ± 3.17 | 0.46 ± 0.09 | 2.02 ± 0.59 | 0.71 ± 0.4 | 0.42 ± 0.09 |  | | |  |
| TG.18:1_36:6 | HMDB0049402 | 0.51 ± 0.19 | 0.44 ± 0.09 | 0.36 ± 0.08 | 1.53 ± 0.91 | 0.25 ± 0.05 | 0.83 ± 0.26 | 0.22 ± 0.04 | 0.24 ± 0.05 |  | | |  |
| TG.18:1_38:5 | HMDB0049390 | 0.92 ± 0.33 | 0.64 ± 0.09 | 0.48 ± 0.05 | 1.01 ± 0.6 | 0.5 ± 0.08 | 0.98 ± 0.32 | 0.51 ± 0.19 | 0.46 ± 0.07 |  | | |  |
| TG.18:1_38:6 | HMDB0049397 | 0.37 ± 0.17 | 0.29 ± 0.1 | 0.27 ± 0.04 | 0.33 ± 0.14 | 0.18 ± 0.06 | 0.54 ± 0.2 | 0.34 ± 0.11 | 0.2 ± 0.04 |  | | |  |
| TG.18:1_38:7 | HMDB0049423 | 0.96 ± 0.36 | 0.75 ± 0.11 | 0.54 ± 0.11 | 0.91 ± 0.39 | 0.41 ± 0.04 | 1.24 ± 0.41 | 0.4 ± 0.16 | 0.44 ± 0.1 |  | | |  |
| TG.18:2_28:0 | HMDB0042076 | 0.68 ± 0.41 | 0.57 ± 0.17 | 0.34 ± 0.04 | 0.75 ± 0.2 | 0.26 ± 0.04 | 0.92 ± 0.22 | 0.35 ± 0.13 | 0.24 ± 0.03 |  | | |  |
| TG.18:2_30:0 | HMDB0005376 | 2.22 ± 1.05 | 1.76 ± 0.53 | 1.2 ± 0.09 | 2.41 ± 1.02 | 1.08 ± 0.16 | 2.35 ± 0.5 | 1.4 ± 0.49 | 0.95 ± 0.07 |  | | |  |
| TG.18:2_30:1 | HMDB0042543 | 0.63 ± 0.31 | 0.39 ± 0.13 | 0.33 ± 0.04 | 0.98 ± 0.63 | 0.44 ± 0.12 | 0.87 ± 0.16 | 0.46 ± 0.17 | 0.33 ± 0.02 |  | | |  |
| TG.18:2_31:0 | HMDB0042976 | 3.18 ± 1.84 | 2.3 ± 0.49 | 1.77 ± 0.32 | 3.46 ± 1.52 | 1.41 ± 0.18 | 4.3 ± 1.17 | 2.08 ± 0.78 | 1.71 ± 0.18 |  | | |  |
| TG.18:2_32:0 | HMDB0052410 | 4.86 ± 1.52 | 3.68 ± 0.63 | 2.89 ± 0.31 | 6.27 ± 2.29 | 3.04 ± 0.59 | 7.29 ± 1.48 | 4.17 ± 1.81 | 2.95 ± 0.41 |  | | |  |
| TG.18:2_32:1 | HMDB0052515 | 2.31 ± 0.91 | 1.72 ± 0.49 | 1.47 ± 0.16 | 3.29 ± 2.1 | 1.19 ± 0.16 | 3.6 ± 1.02 | 2.43 ± 0.97 | 1.36 ± 0.33 |  | | |  |
| TG.18:2_32:2 | HMDB0010470 | 0.63 ± 0.26 | 0.74 ± 0.28 | 0.47 ± 0.09 | 0.95 ± 0.51 | 0.41 ± 0.05 | 1.56 ± 0.63 | 0.75 ± 0.39 | 0.48 ± 0.04 |  | | |  |
| TG.18:2_33:0 | HMDB0052425 | 3.44 ± 1.53 | 2.49 ± 0.43 | 1.88 ± 0.36 | 3.72 ± 1.35 | 1.72 ± 0.32 | 4.83 ± 1.66 | 1.99 ± 0.63 | 1.94 ± 0.21 |  | | |  |
| TG.18:2_33:1 | HMDB0011701 | 2.03 ± 0.79 | 1.59 ± 0.35 | 1.23 ± 0.18 | 2.48 ± 1.15 | 1.13 ± 0.2 | 3.62 ± 1.69 | 1.74 ± 0.76 | 1.18 ± 0.22 |  | | |  |
| TG.18:2_33:2 | HMDB0052411 | 0.49 ± 0.15 | 0.48 ± 0.15 | 0.31 ± 0.05 | 0.64 ± 0.26 | 0.31 ± 0.05 | 0.71 ± 0.19 | 0.76 ± 0.43 | 0.39 ± 0.08 |  | | |  |
| TG.18:2_34:0 | HMDB0052440 | 6.49 ± 2.08 | 5.02 ± 0.81 | 3.81 ± 0.64 | 7.56 ± 2.27 | 3.87 ± 0.68 | 8.67 ± 2.47 | 3.66 ± 1.1 | 3.94 ± 0.41 |  | | |  |
| TG.18:2_34:1 | HMDB0052530 | 5.53 ± 1.94 | 3.91 ± 0.92 | 3.24 ± 0.28 | 8.91 ± 3.5 | 3.24 ± 0.65 | 7.63 ± 1.6 | 5.27 ± 2.54 | 3.66 ± 0.61 |  | | |  |
| TG.18:2_34:2 | HMDB0052426 | 2.7 ± 1.04 | 1.9 ± 0.54 | 1.7 ± 0.16 | 4.69 ± 1.88 | 1.78 ± 0.43 | 5.69 ± 1.53 | 3.39 ± 2.05 | 1.88 ± 0.59 |  | | |  |
| TG.18:2_34:3 | HMDB0052427 | 0.62 ± 0.17 | 0.69 ± 0.2 | 0.57 ± 0.1 | 1.61 ± 0.62 | 0.52 ± 0.13 | 1.5 ± 0.46 | 1.17 ± 0.71 | 0.67 ± 0.24 |  | | |  |
| TG.18:2_35:1 | HMDB0050323 | 1.15 ± 0.5 | 0.91 ± 0.21 | 0.6 ± 0.14 | 1.53 ± 0.77 | 0.58 ± 0.1 | 1.41 ± 0.47 | 0.8 ± 0.3 | 0.7 ± 0.08 |  | | |  |
| TG.18:2_35:2 | HMDB0052413 | 0.57 ± 0.28 | 0.46 ± 0.18 | 0.41 ± 0.12 | 0.73 ± 0.35 | 0.32 ± 0.09 | 0.96 ± 0.46 | 0.52 ± 0.25 | 0.37 ± 0.07 |  | | |  |
| TG.18:2_36:0 | HMDB0052455 | 3.11 ± 0.97 | 2.14 ± 0.34 | 2.02 ± 0.35 | 3.14 ± 0.84 | 1.63 ± 0.21 | 3.2 ± 0.72 | 1.46 ± 0.3 | 1.67 ± 0.3 |  | | |  |
| TG.18:2_36:1 | HMDB0052545 | 2.75 ± 1.15 | 2.13 ± 0.29 | 1.55 ± 0.29 | 4.4 ± 1.64 | 1.37 ± 0.23 | 4.3 ± 1.22 | 1.8 ± 0.62 | 1.59 ± 0.24 |  | | |  |
| TG.18:2_36:2 | HMDB0052441 | 3.13 ± 1.48 | 2.5 ± 0.67 | 1.8 ± 0.23 | 13.12 ± 9.67 | 1.72 ± 0.35 | 6.12 ± 1.89 | 2.58 ± 1.32 | 1.68 ± 0.25 |  | | |  |
| TG.18:2_36:3 | HMDB0052442 | 2.44 ± 1.24 | 1.59 ± 0.51 | 1.07 ± 0.12 | 8.34 ± 5.89 | 1.0 ± 0.11 | 6.26 ± 2.65 | 1.88 ± 0.97 | 1.15 ± 0.14 |  | | |  |
| TG.18:2_36:4 | HMDB0052450 | 1.6 ± 0.76 | 1.17 ± 0.45 | 0.83 ± 0.15 | 6.2 ± 4.2 | 0.79 ± 0.11 | 6.68 ± 3.81 | 1.22 ± 0.59 | 0.63 ± 0.09 |  | | |  |
| TG.18:2_36:5 | HMDB0052540 | 0.36 ± 0.17 | 0.36 ± 0.12 | 0.24 ± 0.05 | 1.44 ± 0.92 | 0.24 ± 0.06 | 1.71 ± 0.95 | 0.41 ± 0.22 | 0.21 ± 0.06 |  | | |  |
| TG.18:2_38:4 | HMDB0052445 | 0.21 ± 0.05 | 0.21 ± 0.08 | 0.2 ± 0.04 | 0.34 ± 0.13 | 0.22 ± 0.04 | 0.46 ± 0.15 | 0.18 ± 0.08 | 0.14 ± 0.05 |  | | |  |
| TG.18:3_30:0 | HMDB0042543 | 0.86 ± 0.42 | 0.73 ± 0.28 | 0.48 ± 0.08 | 0.77 ± 0.29 | 0.36 ± 0.04 | 1.15 ± 0.36 | 0.48 ± 0.19 | 0.39 ± 0.01 |  | | |  |
| TG.18:3_32:0 | HMDB0052858 | 2.06 ± 0.8 | 1.41 ± 0.17 | 1.19 ± 0.17 | 2.18 ± 0.82 | 1.31 ± 0.19 | 2.92 ± 0.82 | 1.53 ± 0.65 | 1.06 ± 0.08 |  | | |  |
| TG.18:3_32:1 | HMDB0052956 | 0.82 ± 0.27 | 0.79 ± 0.22 | 0.52 ± 0.06 | 0.95 ± 0.49 | 0.6 ± 0.13 | 1.39 ± 0.39 | 0.81 ± 0.32 | 0.46 ± 0.07 |  | | |  |
| TG.18:3_34:0 | HMDB0052886 | 2.97 ± 0.99 | 2.12 ± 0.3 | 1.82 ± 0.27 | 3.13 ± 0.87 | 1.83 ± 0.29 | 4.15 ± 0.83 | 1.48 ± 0.36 | 1.71 ± 0.25 |  | | |  |
| TG.18:3_34:1 | HMDB0052970 | 2.09 ± 0.71 | 1.46 ± 0.23 | 1.37 ± 0.17 | 3.24 ± 1.1 | 1.3 ± 0.24 | 3.21 ± 0.86 | 1.88 ± 0.99 | 1.31 ± 0.16 |  | | |  |
| TG.18:3_34:2 | HMDB0052846 | 0.94 ± 0.28 | 0.57 ± 0.15 | 0.68 ± 0.22 | 1.41 ± 0.53 | 0.4 ± 0.11 | 1.58 ± 0.56 | 0.95 ± 0.55 | 0.65 ± 0.21 |  | | |  |
| TG.18:3_36:1 | HMDB0052998 | 1.03 ± 0.46 | 0.96 ± 0.13 | 0.59 ± 0.22 | 1.86 ± 0.65 | 0.71 ± 0.1 | 1.25 ± 0.24 | 0.67 ± 0.22 | 0.6 ± 0.1 |  | | |  |
| TG.18:3_36:2 | HMDB0053068 | 1.24 ± 0.58 | 0.9 ± 0.21 | 0.64 ± 0.04 | 6.32 ± 5.03 | 0.58 ± 0.09 | 2.61 ± 0.76 | 0.74 ± 0.39 | 0.64 ± 0.07 |  | | |  |
| TG.18:3_36:3 | HMDB0052887 | 0.69 ± 0.25 | 0.41 ± 0.05 | 0.48 ± 0.08 | 3.61 ± 2.83 | 0.4 ± 0.06 | 2.1 ± 0.66 | 0.65 ± 0.36 | 0.39 ± 0.09 |  | | |  |
| TG.20:1_32:1 | HMDB0050451 | 0.32 ± 0.16 | 0.41 ± 0.07 | 0.24 ± 0.05 | 0.48 ± 0.2 | 0.15 ± 0.01 | 0.56 ± 0.12 | 0.18 ± 0.05 | 0.19 ± 0.04 |  | | |  |
| TG.20:1_32:2 | HMDB0050304 | 0.36 ± 0.08 | 0.97 ± 0.39 | 0.42 ± 0.15 | 0.6 ± 0.18 | 0.3 ± 0.05 | 1.1 ± 0.28 | 0.44 ± 0.14 | 0.5 ± 0.08 |  | | |  |
| TG.20:1_34:0 | HMDB0050356 | 0.4 ± 0.16 | 0.32 ± 0.1 | 0.22 ± 0.05 | 0.35 ± 0.12 | 0.18 ± 0.01 | 0.46 ± 0.05 | 0.24 ± 0.06 | 0.19 ± 0.05 |  | | |  |
| TG.20:1_34:1 | HMDB0050470 | 0.3 ± 0.09 | 0.29 ± 0.05 | 0.23 ± 0.04 | 0.42 ± 0.09 | 0.26 ± 0.04 | 0.5 ± 0.11 | 0.19 ± 0.05 | 0.21 ± 0.03 |  | | |  |
| TG.20:1_34:2 | HMDB0050342 | 0.2 ± 0.06 | 0.15 ± 0.06 | 0.22 ± 0.04 | 0.26 ± 0.1 | 0.1 ± 0.03 | 0.26 ± 0.07 | 0.13 ± 0.04 | 0.06 ± 0.02 |  | | |  |
| TG.20:3_32:0 | HMDB0050886 | 0.88 ± 0.39 | 0.52 ± 0.09 | 0.42 ± 0.05 | 0.73 ± 0.26 | 0.5 ± 0.1 | 1.41 ± 0.41 | 0.69 ± 0.29 | 0.5 ± 0.07 |  | | |  |
| TG.20:3_32:2 | HMDB0050872 | 2.42 ± 1.74 | 7.81 ± 3.14 | 0.92 ± 0.21 | 5.6 ± 1.79 | 0.68 ± 0.21 | 14.38 ± 5.52 | 2.48 ± 1.83 | 6.61 ± 0.79 |  | | |  |
| TG.20:3_34:0 | HMDB0050922 | 0.94 ± 0.33 | 0.77 ± 0.2 | 0.54 ± 0.07 | 1.03 ± 0.53 | 0.45 ± 0.08 | 1.52 ± 0.47 | 0.45 ± 0.15 | 0.51 ± 0.07 |  | | |  |
| TG.20:3_34:1 | HMDB0051030 | 0.84 ± 0.28 | 0.54 ± 0.15 | 0.5 ± 0.1 | 0.92 ± 0.6 | 0.48 ± 0.13 | 1.08 ± 0.35 | 0.64 ± 0.32 | 0.38 ± 0.09 |  | | |  |
| TG.20:3_34:2 | HMDB0050908 | 0.46 ± 0.24 | 0.28 ± 0.11 | 0.25 ± 0.07 | 0.51 ± 0.26 | 0.19 ± 0.03 | 0.73 ± 0.25 | 0.43 ± 0.27 | 0.27 ± 0.05 |  | | |  |
| TG.20:3_34:3 | HMDB0050909 | 0.23 ± 0.11 | 0.25 ± 0.11 | 0.11 ± 0.02 | 0.21 ± 0.1 | 0.13 ± 0.04 | 0.66 ± 0.41 | 0.18 ± 0.11 | 0.15 ± 0.05 |  | | |  |
| TG.20:3_36:3 | HMDB0050905 | 0.15 ± 0.03 | 0.23 ± 0.09 | 0.1 ± 0.03 | 0.28 ± 0.13 | 0.16 ± 0.04 | 0.52 ± 0.3 | 0.21 ± 0.11 | 0.16 ± 0.02 |  | | |  |
| TG.20:3_36:4 | HMDB0050912 | 0.17 ± 0.07 | 0.13 ± 0.05 | 0.09 ± 0.0 | 0.15 ± 0.05 | 0.11 ± 0.02 | 0.29 ± 0.08 | 0.14 ± 0.04 | 0.06 ± 0.02 |  | | |  |
| TG.20:4_30:0 | HMDB0042633 | 0.42 ± 0.2 | 0.25 ± 0.09 | 0.19 ± 0.04 | 0.35 ± 0.2 | 0.16 ± 0.02 | 0.52 ± 0.21 | 0.17 ± 0.08 | 0.13 ± 0.01 |  | | |  |
| TG.20:4_32:0 | HMDB0054022 | 0.88 ± 0.29 | 0.75 ± 0.18 | 0.6 ± 0.12 | 1.13 ± 0.52 | 0.49 ± 0.1 | 1.57 ± 0.53 | 0.86 ± 0.47 | 0.53 ± 0.06 |  | | |  |
| TG.20:4_32:1 | HMDB0054099 | 0.33 ± 0.08 | 0.51 ± 0.13 | 0.32 ± 0.04 | 0.58 ± 0.32 | 0.26 ± 0.04 | 0.68 ± 0.07 | 0.35 ± 0.12 | 0.31 ± 0.03 |  | | |  |
| TG.20:4_32:2 | HMDB0048751 | 0.51 ± 0.11 | 0.91 ± 0.31 | 0.37 ± 0.07 | 0.63 ± 0.12 | 0.29 ± 0.03 | 1.51 ± 0.53 | 0.41 ± 0.13 | 0.52 ± 0.06 |  | | |  |
| TG.20:4_34:0 | HMDB0054044 | 1.78 ± 0.78 | 1.05 ± 0.14 | 0.89 ± 0.13 | 1.41 ± 0.66 | 0.83 ± 0.19 | 1.92 ± 0.28 | 0.93 ± 0.21 | 0.81 ± 0.11 |  | | |  |
| TG.20:4_34:1 | HMDB0054110 | 0.91 ± 0.28 | 0.75 ± 0.15 | 0.55 ± 0.13 | 1.09 ± 0.59 | 0.6 ± 0.13 | 1.86 ± 0.75 | 0.9 ± 0.46 | 0.61 ± 0.14 |  | | |  |
| TG.20:4_34:2 | HMDB0010418 | 0.43 ± 0.16 | 0.37 ± 0.1 | 0.21 ± 0.05 | 0.65 ± 0.3 | 0.33 ± 0.09 | 0.66 ± 0.11 | 0.54 ± 0.31 | 0.32 ± 0.09 |  | | |  |
| TG.20:4_36:2 | HMDB0054187 | 0.47 ± 0.11 | 0.34 ± 0.08 | 0.24 ± 0.04 | 0.36 ± 0.12 | 0.33 ± 0.06 | 0.73 ± 0.24 | 0.39 ± 0.19 | 0.35 ± 0.07 |  | | |  |
| TG.20:4_36:4 | HMDB0054050 | 0.1 ± 0.01 | 0.13 ± 0.03 | 0.09 ± 0.03 | 0.2 ± 0.06 | 0.14 ± 0.04 | 0.49 ± 0.23 | 0.15 ± 0.07 | 0.08 ± 0.02 |  | | |  |
| TG.20:5_34:0 | HMDB0055696 | 0.97 ± 0.38 | 0.81 ± 0.11 | 0.46 ± 0.11 | 0.9 ± 0.36 | 0.48 ± 0.08 | 1.48 ± 0.48 | 0.42 ± 0.1 | 0.49 ± 0.05 |  | | |  |
| TG.20:5_34:1 | HMDB0055720 | 1.85 ± 0.76 | 1.66 ± 0.25 | 1.05 ± 0.21 | 1.89 ± 0.59 | 0.85 ± 0.08 | 3.32 ± 1.45 | 0.75 ± 0.2 | 0.99 ± 0.24 |  | | |  |
| TG.20:5_34:2 | HMDB0005447 | 0.99 ± 0.48 | 0.9 ± 0.14 | 0.54 ± 0.14 | 1.05 ± 0.17 | 0.48 ± 0.12 | 1.54 ± 0.71 | 0.63 ± 0.24 | 0.54 ± 0.08 |  | | |  |
| TG.20:5_36:2 | HMDB0055748 | 0.88 ± 0.53 | 0.55 ± 0.08 | 0.41 ± 0.06 | 0.67 ± 0.13 | 0.33 ± 0.05 | 1.25 ± 0.67 | 0.35 ± 0.09 | 0.4 ± 0.09 |  | | |  |
| TG.22:4_34:2 | HMDB0046908 | 0.18 ± 0.06 | 0.08 ± 0.02 | 0.08 ± 0.02 | 0.14 ± 0.03 | 0.1 ± 0.03 | 0.4 ± 0.27 | 0.1 ± 0.03 | 0.07 ± 0.02 |  | | |  |
| TG.22:5_32:0 | HMDB0054916 | 2.24 ± 0.98 | 1.53 ± 0.19 | 1.15 ± 0.15 | 2.4 ± 1.27 | 1.14 ± 0.23 | 2.98 ± 0.67 | 1.23 ± 0.55 | 0.96 ± 0.06 |  | | |  |
| TG.22:5_32:1 | HMDB0054972 | 7.91 ± 2.38 | 12.97 ± 2.63 | 5.82 ± 0.99 | 9.09 ± 1.56 | 4.73 ± 0.6 | 15.65 ± 2.58 | 6.25 ± 1.81 | 8.7 ± 0.95 |  | | |  |
| TG.22:5_34:1 | HMDB0054980 | 1.34 ± 0.65 | 0.91 ± 0.2 | 0.7 ± 0.11 | 1.43 ± 0.87 | 0.58 ± 0.09 | 1.3 ± 0.43 | 0.65 ± 0.33 | 0.45 ± 0.08 |  | | |  |
| TG.22:5_34:2 | HMDB0005462 | 0.61 ± 0.27 | 0.46 ± 0.1 | 0.28 ± 0.07 | 0.57 ± 0.15 | 0.33 ± 0.07 | 0.76 ± 0.2 | 0.35 ± 0.12 | 0.34 ± 0.07 |  | | |  |
| TG.22:6_32:0 | HMDB0055894 | 1.2 ± 0.72 | 0.66 ± 0.21 | 0.42 ± 0.06 | 0.97 ± 0.55 | 0.43 ± 0.08 | 1.12 ± 0.35 | 0.54 ± 0.21 | 0.44 ± 0.1 |  | | |  |
| TG.22:6_32:1 | HMDB0055908 | 0.59 ± 0.18 | 0.77 ± 0.18 | 0.39 ± 0.09 | 0.59 ± 0.22 | 0.37 ± 0.06 | 1.13 ± 0.33 | 0.28 ± 0.07 | 0.31 ± 0.04 |  | | |  |
| TG.22:6_34:1 | HMDB0055910 | 0.77 ± 0.43 | 0.66 ± 0.29 | 0.35 ± 0.08 | 0.71 ± 0.39 | 0.37 ± 0.07 | 1.22 ± 0.5 | 0.43 ± 0.16 | 0.35 ± 0.09 |  | | |  |
| TG.22:6_34:2 | HMDB0005392 | 0.37 ± 0.14 | 0.25 ± 0.08 | 0.2 ± 0.07 | 0.33 ± 0.16 | 0.22 ± 0.04 | 0.48 ± 0.16 | 0.25 ± 0.11 | 0.14 ± 0.02 |  | | |  |
| Uric acid | HMDB0000289 | 27.9 ± 0.62 | 31.7 ± 3.51 | 31.44 ± 2.15 | 35.39 ± 6.25 | 27.77 ± 2.27 | 36.42 ± 2.08 | 27.82 ± 2.8 | 37.16 ± 4.17 |  | | |  |
| p-Cresol sulfate | HMDB0011635 | 69.23 ± 7.09 | 97.35 ± 16.86 | 65.86 ± 6.61 | 79.56 ± 6.02 | 60.53 ± 7.23 | 87.7 ± 6.89 | 68.34 ± 5.45 | 66.42 ± 6.01 |  | | |  |
| 4-Ethylphenyl sulfate | HMDB0062551 | 5.62 ± 3.65 | 5.77 ± 2.76 | 2.93 ± 0.71 | 3.12 ± 0.35 | 2.16 ± 0.38 | 10.16 ± 4.5 | 2.33 ± 0.53 | 2.69 ± 0.42 |  | | |  |
| Propionic acid | HMDB0000237 | 21.94 ± 2.19 | 13.11 ± 2.72 | 21.94 ± 2.19 | 14.13 ± 1.57 | 11.79 ± 1.66 | 15.67 ± 1.63 | 9.88 ± 0.75 | 10.45 ± 2.83 |  | | |  |
| Butyric acid | HMDB0000039 | 9.41 ± 3.72 | 9.59 ± 4.48 | 6.83 ± 1.05 | 6.52 ± 0.48 | 6.58 ± 0.88 | 14.55 ± 4.87 | 4.72 ± 0.8 | 5.1 ± 1.87 |  | | |  |
| Lactic acid | HMDB0000190 | 1463.0 ± 406.35 | 1938.25 ± 377.83 | 2041.65 ± 644.19 | 990.68 ± 106.58 | 1386.45 ± 338.36 | 2262.0 ± 340.49 | 1762.15 ± 553.86 | 1998.25 ± 592.17 |  | | |  |
| Valeric acid | HMDB0000892 | 7.05 ± 1.41 | 6.59 ± 2.05 | 4.9 ± 0.83 | 5.86 ± 0.59 | 6.55 ± 1.95 | 8.14 ± 1.1 | 5.29 ± 0.78 | 3.28 ± 0.73 |  | | |  |
| Dimethylglycine | HMDB0000092 | 9.01 ± 1.39 | 11.08 ± 1.38 | 9.1 ± 1.99 | 10.68 ± 0.89 | 8.55 ± 0.97 | 8.43 ± 1.47 | 8.15 ± 0.53 | 9.6 ± 1.97 |  | | |  |
| 2-Hydroxybutyric acid | HMDB0000008 | 5.58 ± 0.39 | 5.02 ± 0.27 | 5.61 ± 0.42 | 4.58 ± 0.17 | 5.01 ± 0.29 | 6.16 ± 0.4 | 5.41 ± 0.58 | 5.65 ± 0.58 |  | | |  |
| 2-Hydroxyisobutyric acid | HMDB0000729 | 3.14 ± 0.1 | 3.27 ± 0.18 | 3.3 ± 0.16 | 3.15 ± 0.29 | 3.5 ± 0.23 | 3.1 ± 0.12 | 3.49 ± 0.13 | 3.28 ± 0.66 |  | | |  |
| 3-Hydroxybutyric acid | HMDB0000011 | 360.82 ± 50.53 | 351.0 ± 37.56 | 373.5 ± 34.8 | 349.1 ± 14.02 | 329.1 ± 22.54 | 447.32 ± 56.33 | 337.9 ± 49.21 | 310.25 ± 43.64 |  | | |  |
| 3-Hydroxyisobutyric acid | HMDB0000336 | 31.95 ± 3.42 | 34.32 ± 4.64 | 31.18 ± 3.06 | 30.27 ± 0.93 | 28.84 ± 1.86 | 42.54 ± 6.59 | 29.11 ± 3.07 | 30.57 ± 1.85 |  | | |  |
| Glyceric acid | HMDB0000139 | 2.51 ± 0.28 | 2.44 ± 0.17 | 3.13 ± 0.3 | 3.07 ± 0.57 | 2.66 ± 0.13 | 2.42 ± 0.13 | 2.49 ± 0.27 | 2.58 ± 0.34 |  | | |  |
| Caproic acid | HMDB0000535 | 0.7 ± 0.08 | 0.86 ± 0.05 | 0.63 ± 0.07 | 0.76 ± 0.03 | 0.66 ± 0.04 | 0.75 ± 0.1 | 0.68 ± 0.06 | 0.65 ± 0.06 |  | | |  |
| Guanidoacetic acid | HMDB0000128 | 4.09 ± 0.4 | 4.54 ± 0.38 | 5.09 ± 0.12 | 4.26 ± 0.26 | 4.88 ± 0.44 | 3.57 ± 0.52 | 3.73 ± 0.44 | 3.62 ± 0.46 |  | | |  |
| N-Acetyl-glycine | HMDB0000532 | 8.23 ± 1.93 | 6.98 ± 0.54 | 10.35 ± 1.22 | 8.01 ± 0.56 | 12.37 ± 2.45 | 8.85 ± 2.06 | 11.51 ± 2.41 | 9.58 ± 2.16 |  | | |  |
| 2-Hydroxy-2-methylbutyric acid | HMDB0001987 | 0.58 ± 0.06 | 0.47 ± 0.03 | 0.57 ± 0.07 | 0.42 ± 0.06 | 0.47 ± 0.07 | 0.37 ± 0.06 | 0.45 ± 0.05 | 0.51 ± 0.1 |  | | |  |
| 2-Hydroxyisovaleric acid | HMDB0000407 | 1.86 ± 0.22 | 1.99 ± 0.16 | 1.85 ± 0.19 | 1.74 ± 0.22 | 1.87 ± 0.23 | 1.63 ± 0.19 | 1.69 ± 0.07 | 2.1 ± 0.4 |  | | |  |
| 3-Hydroxyisovaleric acid | HMDB0000754 | 21.84 ± 5.23 | 23.27 ± 4.03 | 21.42 ± 3.37 | 20.98 ± 1.32 | 16.98 ± 2.09 | 26.45 ± 4.73 | 17.68 ± 1.83 | 18.39 ± 1.72 |  | | |  |
| Benzoic acid | HMDB0001870 | 2.88 ± 0.69 | 3.76 ± 0.75 | 2.47 ± 0.31 | 2.44 ± 0.21 | 2.55 ± 0.26 | 4.03 ± 0.53 | 2.04 ± 0.27 | 2.31 ± 0.1 |  | | |  |
| 5-Oxoproline | HMDB0000267 | 127.63 ± 4.85 | 133.88 ± 9.84 | 139.08 ± 13.36 | 140.15 ± 2.36 | 125.72 ± 4.83 | 132.25 ± 8.33 | 120.1 ± 15.46 | 125.83 ± 30.78 |  | | |  |
| Pipecolic acid | HMDB0000070 | 6.37 ± 0.27 | 6.98 ± 0.41 | 7.57 ± 0.67 | 7.27 ± 0.16 | 6.93 ± 0.15 | 7.26 ± 0.65 | 6.56 ± 1.1 | 6.7 ± 1.61 |  | | |  |
| Guanidinopropionic acid | HMDB0013222 | 150.54 ± 19.36 | 167.42 ± 4.18 | 153.41 ± 18.34 | 175.75 ± 8.55 | 123.39 ± 14.01 | 137.57 ± 9.52 | 164.68 ± 5.08 | 155.57 ± 15.46 |  | | |  |
| N-Acetyl-alanine | HMDB0000766 | 2.82 ± 0.37 | 3.91 ± 0.47 | 3.41 ± 0.47 | 4.32 ± 0.55 | 2.91 ± 0.33 | 3.37 ± 0.31 | 2.37 ± 0.33 | 3.31 ± 0.86 |  | | |  |
| 2-Hydroxy-3-methylvaleric acid | HMDB0000317 | 0.28 ± 0.02 | 0.29 ± 0.02 | 0.27 ± 0.02 | 0.28 ± 0.01 | 0.31 ± 0.02 | 0.31 ± 0.02 | 0.3 ± 0.04 | 0.29 ± 0.02 |  | | |  |
| Phenylacetic acid | HMDB0000209 | 4.58 ± 1.59 | 4.86 ± 1.59 | 3.04 ± 0.27 | 3.72 ± 0.44 | 2.81 ± 0.37 | 6.68 ± 2.02 | 2.71 ± 0.38 | 2.96 ± 0.43 |  | | |  |
| Caprylic acid | HMDB0000482 | 0.46 ± 0.03 | 0.4 ± 0.04 | 0.44 ± 0.05 | 0.42 ± 0.02 | 0.47 ± 0.04 | 0.41 ± 0.05 | 0.47 ± 0.03 | 0.33 ± 0.05 |  | | |  |
| N-Acetyl-serine | HMDB0002931 | 0.84 ± 0.04 | 0.82 ± 0.02 | 0.86 ± 0.04 | 0.87 ± 0.05 | 0.82 ± 0.07 | 0.84 ± 0.07 | 0.8 ± 0.07 | 0.85 ± 0.13 |  | | |  |
| Xanthosine | HMDB0000299 | 0.45 ± 0.03 | 0.48 ± 0.03 | 0.44 ± 0.02 | 0.42 ± 0.02 | 0.49 ± 0.05 | 0.48 ± 0.03 | 0.44 ± 0.03 | 0.49 ± 0.04 |  | | |  |
| 4-Hydroxyphenylacetic acid | HMDB0000020 | 0.7 ± 0.08 | 0.77 ± 0.1 | 0.54 ± 0.05 | 0.6 ± 0.06 | 0.69 ± 0.04 | 0.78 ± 0.06 | 0.58 ± 0.1 | 0.68 ± 0.11 |  | | |  |
| N-Acetyl-proline | HMDB0094701 | 0.62 ± 0.07 | 0.87 ± 0.12 | 0.81 ± 0.13 | 0.91 ± 0.11 | 0.65 ± 0.06 | 0.75 ± 0.04 | 0.5 ± 0.07 | 0.73 ± 0.2 |  | | |  |
| Tiglylglycine | HMDB0000959 | 0.31 ± 0.1 | 0.18 ± 0.03 | 0.32 ± 0.09 | 0.21 ± 0.05 | 0.39 ± 0.09 | 0.29 ± 0.03 | 0.25 ± 0.05 | 0.25 ± 0.08 |  | | |  |
| N-Acetyl-valine | HMDB0011757 | 0.21 ± 0.02 | 0.26 ± 0.02 | 0.25 ± 0.03 | 0.27 ± 0.03 | 0.22 ± 0.04 | 0.24 ± 0.01 | 0.18 ± 0.02 | 0.21 ± 0.03 |  | | |  |
| N-Acetyl-leucine | HMDB0011756 | 0.47 ± 0.1 | 0.53 ± 0.08 | 0.56 ± 0.1 | 0.59 ± 0.09 | 0.48 ± 0.07 | 0.56 ± 0.06 | 0.36 ± 0.05 | 0.48 ± 0.09 |  | | |  |
| N-Acetyl-isoleucine | HMDB0061684 | 0.1 ± 0.01 | 0.12 ± 0.01 | 0.11 ± 0.02 | 0.12 ± 0.01 | 0.1 ± 0.01 | 0.11 ± 0.01 | 0.09 ± 0.02 | 0.1 ± 0.02 |  | | |  |
| N-Acetyl-asparagine | HMDB0006028 | 0.12 ± 0.01 | 0.15 ± 0.01 | 0.13 ± 0.02 | 0.15 ± 0.01 | 0.14 ± 0.02 | 0.14 ± 0.01 | 0.13 ± 0.02 | 0.1 ± 0.01 |  | | |  |
| 3-Indoleacetic acid | HMDB0000197 | 0.99 ± 0.18 | 1.04 ± 0.02 | 0.72 ± 0.14 | 0.8 ± 0.14 | 0.81 ± 0.11 | 1.09 ± 0.17 | 0.76 ± 0.11 | 0.85 ± 0.12 |  | | |  |
| Argininic acid | HMDB0003148 | 1.18 ± 0.06 | 1.24 ± 0.05 | 1.16 ± 0.11 | 1.38 ± 0.18 | 1.04 ± 0.05 | 1.2 ± 0.08 | 1.09 ± 0.1 | 1.1 ± 0.11 |  | | |  |
| Hippuric acid | HMDB0000714 | 99.89 ± 10.95 | 115.39 ± 15.74 | 91.24 ± 4.47 | 91.12 ± 1.25 | 88.68 ± 3.49 | 97.74 ± 5.66 | 85.41 ± 8.57 | 88.22 ± 2.55 |  | | |  |
| N1-Acetyl-lysine | HMDB0000446 | 0.51 ± 0.09 | 0.71 ± 0.05 | 0.71 ± 0.11 | 0.9 ± 0.08 | 0.42 ± 0.02 | 0.51 ± 0.03 | 0.61 ± 0.09 | 0.78 ± 0.15 |  | | |  |
| N1-Acetyl-lysine | HMDB0006029 | 2.27 ± 0.24 | 2.6 ± 0.19 | 2.74 ± 0.39 | 2.99 ± 0.07 | 2.3 ± 0.24 | 2.54 ± 0.27 | 2.5 ± 0.27 | 2.64 ± 0.21 |  | | |  |
| N-Acetyl-glutamine | HMDB0006029 | 0.66 ± 0.05 | 0.69 ± 0.04 | 0.82 ± 0.12 | 0.77 ± 0.04 | 0.68 ± 0.06 | 0.72 ± 0.01 | 0.48 ± 0.05 | 0.64 ± 0.17 |  | | |  |
| Kynurenic acid | HMDB0000715 | 0.14 ± 0.02 | 0.18 ± 0.02 | 0.14 ± 0.02 | 0.16 ± 0.01 | 0.13 ± 0.01 | 0.16 ± 0.01 | 0.14 ± 0.02 | 0.15 ± 0.02 |  | | |  |
| Indole-3-propionic acid | HMDB0002302 | 2.4 ± 0.65 | 2.19 ± 0.25 | 1.73 ± 0.11 | 1.7 ± 0.18 | 2.05 ± 0.13 | 2.74 ± 0.42 | 1.22 ± 0.21 | 1.4 ± 0.17 |  | | |  |
| 5-Hydroxyindoleacetic acid | HMDB0000763 | 0.1 ± 0.0 | 0.11 ± 0.02 | 0.1 ± 0.01 | 0.11 ± 0.02 | 0.11 ± 0.01 | 0.12 ± 0.02 | 0.1 ± 0.01 | 0.1 ± 0.01 |  | | |  |
| p-Hydroxyhippuric acid | HMDB0013678 | 0.1 ± 0.02 | 0.09 ± 0.01 | 0.08 ± 0.01 | 0.06 ± 0.01 | 0.08 ± 0.01 | 0.11 ± 0.03 | 0.07 ± 0.02 | 0.07 ± 0.01 |  | | |  |
| N-Acetyl-histidine | HMDB0032055 | 11.77 ± 0.74 | 12.11 ± 0.46 | 14.24 ± 1.61 | 17.18 ± 0.71 | 11.6 ± 0.61 | 12.18 ± 0.48 | 11.15 ± 1.56 | 13.1 ± 1.74 |  | | |  |
| Indolelactic acid | HMDB0000671 | 0.51 ± 0.09 | 0.51 ± 0.06 | 0.46 ± 0.03 | 0.43 ± 0.03 | 0.55 ± 0.1 | 0.45 ± 0.04 | 0.45 ± 0.08 | 0.48 ± 0.08 |  | | |  |
| N-Acetyl-Arginine | HMDB0004620 | 0.56 ± 0.02 | 0.61 ± 0.02 | 0.65 ± 0.05 | 0.67 ± 0.04 | 0.56 ± 0.02 | 0.6 ± 0.04 | 0.56 ± 0.05 | 0.59 ± 0.11 |  | | |  |
| Pyruvic acid | HMDB0000243 | 53.8 ± 14.95 | 67.91 ± 6.41 | 54.81 ± 9.59 | 43.5 ± 1.44 | 57.02 ± 8.04 | 62.63 ± 5.98 | 55.5 ± 12.36 | 68.99 ± 8.36 |  | | |  |
| N-Acetyl-tyrosine | HMDB0000866 | 0.29 ± 0.04 | 0.37 ± 0.05 | 0.34 ± 0.04 | 0.38 ± 0.06 | 0.29 ± 0.03 | 0.32 ± 0.03 | 0.2 ± 0.03 | 0.3 ± 0.07 |  | | |  |
| Oxalic acid | HMDB0002329 | 18.02 ± 2.38 | 20.98 ± 3.84 | 21.49 ± 4.27 | 21.31 ± 3.96 | 15.55 ± 1.21 | 16.72 ± 0.89 | 18.85 ± 2.8 | 15.99 ± 1.76 |  | | |  |
| Malonic acid | HMDB0000691 | 0.38 ± 0.05 | 0.33 ± 0.02 | 0.35 ± 0.06 | 0.37 ± 0.04 | 0.36 ± 0.05 | 0.48 ± 0.06 | 0.43 ± 0.11 | 0.35 ± 0.08 |  | | |  |
| alpha-Ketoisovaleric acid | HMDB0000019 | 10.37 ± 1.02 | 12.23 ± 1.01 | 10.61 ± 0.7 | 9.62 ± 0.28 | 10.83 ± 1.06 | 12.66 ± 1.54 | 12.28 ± 1.15 | 13.78 ± 1.62 |  | | |  |
| Fumaric acid | HMDB0000134 | 0.94 ± 0.22 | 1.19 ± 0.18 | 0.76 ± 0.09 | 0.59 ± 0.05 | 0.87 ± 0.15 | 0.87 ± 0.06 | 0.82 ± 0.14 | 0.79 ± 0.17 |  | | |  |
| Maleic acid | HMDB0000176 | 0.24 ± 0.05 | 0.27 ± 0.03 | 0.2 ± 0.02 | 0.19 ± 0.01 | 0.23 ± 0.03 | 0.26 ± 0.03 | 0.19 ± 0.02 | 0.23 ± 0.03 |  | | |  |
| Methylmalonic acid | HMDB0000202 | 1.49 ± 0.18 | 1.38 ± 0.17 | 1.86 ± 0.43 | 1.84 ± 0.22 | 1.78 ± 0.17 | 1.69 ± 0.16 | 1.25 ± 0.27 | 1.15 ± 0.24 |  | | |  |
| Succinic acid | HMDB0000254 | 4.34 ± 0.93 | 5.15 ± 0.83 | 4.07 ± 0.64 | 3.43 ± 0.37 | 3.97 ± 0.74 | 4.12 ± 0.27 | 4.17 ± 0.53 | 4.03 ± 0.61 |  | | |  |
| Phenylacetylglutamine | HMDB0006344 | 0.27 ± 0.13 | 0.32 ± 0.1 | 0.13 ± 0.02 | 0.21 ± 0.01 | 0.12 ± 0.04 | 0.43 ± 0.16 | 0.12 ± 0.03 | 0.13 ± 0.04 |  | | |  |
| 2-oxoisocaproic acid | HMDB0000695 | 25.93 ± 1.92 | 30.58 ± 1.94 | 28.76 ± 1.65 | 25.56 ± 0.71 | 30.77 ± 1.75 | 30.77 ± 2.43 | 32.14 ± 2.72 | 34.87 ± 3.88 |  | | |  |
| Ethylmalonic acid | HMDB0000622 | 1.27 ± 0.13 | 1.33 ± 0.08 | 1.28 ± 0.21 | 1.33 ± 0.11 | 1.06 ± 0.1 | 1.12 ± 0.07 | 1.33 ± 0.14 | 1.16 ± 0.19 |  | | |  |
| Glutaric acid | HMDB0000661 | 0.79 ± 0.1 | 0.8 ± 0.05 | 1.07 ± 0.1 | 0.79 ± 0.09 | 0.92 ± 0.12 | 0.71 ± 0.07 | 1.03 ± 0.2 | 0.9 ± 0.14 |  | | |  |
| Malic acid | HMDB0000156 | 4.48 ± 0.95 | 4.93 ± 0.72 | 3.46 ± 0.32 | 2.91 ± 0.36 | 4.06 ± 0.62 | 3.81 ± 0.31 | 3.28 ± 0.34 | 3.51 ± 0.83 |  | | |  |
| 2-Hydroxyglutaric acid | HMDB0059655 | 2.0 ± 0.23 | 1.78 ± 0.36 | 1.93 ± 0.22 | 1.49 ± 0.2 | 2.45 ± 0.34 | 1.66 ± 0.27 | 2.0 ± 0.17 | 1.75 ± 0.29 |  | | |  |
| 2,5-Furandicarboxylic acid | HMDB0004812 | 0.04 ± 0.02 | 0.04 ± 0.02 | 0.02 ± 0.0 | 0.02 ± 0.0 | 0.02 ± 0.0 | 0.04 ± 0.01 | 0.02 ± 0.0 | 0.02 ± 0.0 |  | | |  |
| 2-oxoadipic acid | HMDB0000225 | 7.73 ± 1.93 | 10.7 ± 1.35 | 8.3 ± 0.41 | 11.83 ± 0.36 | 7.6 ± 1.71 | 8.42 ± 0.81 | 8.3 ± 0.28 | 11.21 ± 0.88 |  | | |  |
| 3-Methyladipic acid | HMDB0000555 | 0.16 ± 0.02 | 0.21 ± 0.02 | 0.14 ± 0.02 | 0.14 ± 0.01 | 0.14 ± 0.01 | 0.16 ± 0.01 | 0.11 ± 0.01 | 0.16 ± 0.02 |  | | |  |
| 3-Deoxyglucosone | HMDB0005876 | 0.08 ± 0.01 | 0.08 ± 0.01 | 0.07 ± 0.01 | 0.08 ± 0.0 | 0.09 ± 0.0 | 0.09 ± 0.01 | 0.07 ± 0.01 | 0.08 ± 0.01 |  | | |  |
| Quinolinic acid | HMDB0000232 | 0.35 ± 0.04 | 0.4 ± 0.04 | 0.3 ± 0.01 | 0.31 ± 0.01 | 0.37 ± 0.06 | 0.33 ± 0.05 | 0.35 ± 0.03 | 0.38 ± 0.02 |  | | |  |
| N-Acetyl-aspartic acid | HMDB0000812 | 0.42 ± 0.07 | 0.37 ± 0.05 | 0.38 ± 0.02 | 0.27 ± 0.02 | 0.4 ± 0.05 | 0.44 ± 0.03 | 0.37 ± 0.05 | 0.3 ± 0.05 |  | | |  |
| 4-Hydroxyphenylpyruvic acid | HMDB0000707 | 1.09 ± 0.15 | 1.22 ± 0.25 | 1.11 ± 0.05 | 1.04 ± 0.07 | 1.06 ± 0.06 | 1.1 ± 0.03 | 0.84 ± 0.09 | 1.12 ± 0.14 |  | | |  |
| N-Acetyl-glutamic acid | HMDB0001138 | 0.07 ± 0.0 | 0.08 ± 0.01 | 0.08 ± 0.0 | 0.07 ± 0.0 | 0.07 ± 0.0 | 0.07 ± 0.0 | 0.07 ± 0.0 | 0.07 ± 0.01 |  | | |  |
| alpha-Ketoglutaric acid | HMDB0000208 | 15.46 ± 2.92 | 16.2 ± 2.35 | 12.84 ± 1.5 | 10.55 ± 0.49 | 15.58 ± 2.28 | 12.62 ± 0.68 | 11.73 ± 2.0 | 10.36 ± 1.01 |  | | |  |
| cis-aconitic acid | HMDB0000072 | 4.2 ± 0.54 | 4.81 ± 0.16 | 3.65 ± 0.38 | 4.2 ± 0.36 | 3.56 ± 0.3 | 3.58 ± 0.02 | 3.74 ± 0.54 | 3.41 ± 0.59 |  | | |  |
| Citric acid | HMDB0000094 | 430.84 ± 46.81 | 475.75 ± 24.61 | 385.87 ± 39.34 | 422.38 ± 28.28 | 362.5 ± 51.48 | 360.05 ± 44.82 | 396.05 ± 77.07 | 333.3 ± 66.47 |  | | |  |
| Acetoacetic acid | HMDB0000060 | 8.48 ± 1.89 | 9.54 ± 0.69 | 8.64 ± 1.31 | 10.87 ± 1.7 | 8.4 ± 1.3 | 14.71 ± 1.97 | 6.13 ± 1.22 | 6.97 ± 1.04 |  | | |  |
| Threonic acid | HMDB0000943 | 3.62 ± 0.08 | 3.61 ± 0.13 | 3.99 ± 0.25 | 4.06 ± 0.27 | 3.62 ± 0.16 | 3.52 ± 0.19 | 3.7 ± 0.26 | 3.72 ± 0.42 |  | | |  |

¹Metabolite abbreviations: LysoPC, lysophosphatidylcholine; SM, sphingomyelin; OH, hydroxyl; PC, phosphatidylcholine; aa, diacyl; ae, acyl-alkyl; C0, free carnitine; C2, acetylcarnitine; C3, propionylcarnitine; C4.1, butenylcarnitine; C4, butyrylcarnitine/isobutyrylcarnitine; C5, valerylcarnitine/isovalerylcarnitine; C4OH, hydroxybutyrylcarnitine; C5OH, hydroxyvalerylcarnitine; C5MDC, methylglutarylcarnitine; C7DC, pimelylcarnitine; C12DC, dodecanedioylcarnitine; Cer, ceramide; CE, cholesteryl ester; Hex2Cer, dihexosylceramide; HexCer, hexosylceramide; TG, triacylglycerol; ²HMDB: Human Metabolome Database; ^3^W1: Week 1; ^4^W2: Week 2.

**Table S6.** List of significant metabolites impacted by methane emission, seaweed supplementation, and their interactions, along with their corresponding FDR-adjusted p-values, from the ANOVA test.

| **Metabolites ^1^** | **Metabolite category** | **Methane emission** | **Seaweed treatment** | **Interaction** |
| --- | --- | --- | --- | --- |
| TG.16:1_34:1 | Triglyceride | <0.001 | <0.001 | <0.001 |
| TG.17:1_36:4 | Triglyceride | <0.001 | <0.001 | <0.001 |
| TG.18:2_30:1 | Triglyceride | <0.001 | <0.001 | <0.001 |
| TG.16:1_34:2 | Triglyceride | <0.001 | <0.001 | <0.001 |
| PC ae C40.5 | Phosphatidylcholine | <0.001 | <0.001 | <0.001 |
| TG.18:2_32:1 | Triglyceride | <0.001 | <0.001 | <0.001 |
| TG.17:1_34:1 | Triglyceride | <0.001 | <0.001 | <0.001 |
| PC ae C40.4 | Phosphatidylcholine | <0.001 | 0.003 | <0.001 |
| Threonic acid | Organic acid | <0.001 | 0.002 | <0.001 |
| TG.14:0_34:2 | Triglyceride | <0.001 | <0.001 | <0.001 |
| TG.16:1_36:4 | Triglyceride | <0.001 | <0.001 | <0.001 |
| TG.20:4_32:1 | Triglyceride | <0.001 | <0.001 | <0.001 |
| TG.20:1_31:0 | Triglyceride | <0.001 | <0.001 | <0.001 |
| PC ae C40.6 | Phosphatidylcholine | <0.001 | 0.004 | <0.001 |
| TG.16:0_34:4 | Triglyceride | <0.001 | 0.001 | <0.001 |
| TG.16:0_35:2 | Triglyceride | <0.001 | 0.001 | <0.001 |
| TG.16:0_32:3 | Triglyceride | <0.001 | <0.001 | <0.001 |
| TG.16:0_38:5 | Triglyceride | <0.001 | <0.001 | <0.001 |
| TG.20:4_34:2 | Triglyceride | 0.001 | <0.001 | <0.001 |
| PC ae C40.3 | Phosphatidylcholine | 0.001 | 0.003 | <0.001 |
| TG.16:0_34:3 | Triglyceride | 0.001 | <0.001 | <0.001 |
| TG.16:1_34:3 | Triglyceride | 0.001 | <0.001 | <0.001 |
| PC ae C42.5 | Phosphatidylcholine | 0.001 | 0.002 | <0.001 |
| PC aa C40.6 | Phosphatidylcholine | 0.001 | 0.004 | <0.001 |
| TG.14:0_34:3 | Triglyceride | 0.001 | <0.001 | <0.001 |
| TG.14:0_38.4 | Triglyceride | 0.001 | <0.001 | <0.001 |
| PC aa C38.5 | Phosphatidylcholine | 0.001 | 0.001 | <0.001 |
| TG.17:1_34:2 | Triglyceride | 0.001 | 0.002 | <0.001 |
| TG.20:3_34:1 | Triglyceride | 0.001 | 0.001 | <0.001 |
| TG.18:2_34:4 | Triglyceride | 0.001 | 0.003 | <0.001 |
| TG.18:2_32:2 | Triglyceride | 0.001 | 0.004 | <0.001 |
| TG.14:0_32:2 | Triglyceride | 0.001 | NS ^2^ | <0.001 |
| TG.20:1_30:1 | Triglyceride | 0.002 | <0.001 | <0.001 |
| TG.14:0_36:2 | Triglyceride | 0.002 | 0.001 | <0.001 |
| TG.16:1_34:0 | Triglyceride | 0.003 | 0.004 | <0.001 |
| PC aa C40.4 | Phosphatidylcholine | 0.003 | 0.007 | <0.001 |
| TG.16:0_32:2 | Triglyceride | 0.003 | 0.012 | 0.001 |
| TG.20:4_32:0 | Triglyceride | 0.003 | 0.002 | <0.001 |
| TG.18:1_33:1 | Triglyceride | 0.003 | 0.007 | <0.001 |
| TG.20:2_32:0 | Triglyceride | 0.003 | 0.012 | <0.001 |
| TG.16:1_38.3 | Triglyceride | 0.004 | 0.004 | <0.001 |
| TG.14:0_35:2 | Triglyceride | 0.004 | NS | <0.001 |
| TG.18:3_32:1 | Triglyceride | 0.004 | 0.004 | <0.001 |
| PC ae C38.6 | Phosphatidylcholine | 0.004 | NS | <0.001 |
| TG.14:0_35:1 | Triglyceride | 0.004 | NS | <0.001 |
| TG.14:0_36:3 | Triglyceride | 0.004 | 0.001 | <0.001 |
| TG.16:0_38:6 | Triglyceride | 0.004 | 0.001 | <0.001 |
| TG.17:1_34:3 | Triglyceride | 0.004 | 0.003 | <0.001 |
| TG.18:0_32:2 | Triglyceride | 0.004 | 0.007 | <0.001 |
| TG.18:1_38:5 | Triglyceride | 0.004 | <0.001 | <0.001 |
| TG.18:2_35:1 | Triglyceride | 0.004 | 0.004 | <0.001 |
| TG.22:6_32:0 | Triglyceride | 0.004 | 0.004 | <0.001 |
| TG.22:6_34:1 | Triglyceride | 0.004 | 0.012 | <0.001 |
| Glucose | Carbohydrate | 0.006 | NS | <0.001 |
| PC aa C38.6 | Phosphatidylcholine | 0.006 | NS | <0.001 |
| TG.14:0_38:5 | Triglyceride | 0.006 | 0.003 | <0.001 |
| TG.16:0_30:2 | Triglyceride | 0.006 | 0.003 | <0.001 |
| TG.17:0_34:2 | Triglyceride | 0.006 | 0.007 | <0.001 |
| TG.18:1_30:2 | Triglyceride | 0.006 | NS | <0.001 |
| TG.18:1_33:2 | Triglyceride | 0.006 | 0.004 | <0.001 |
| TG.18:2_33:2 | Triglyceride | 0.006 | 0.024 | <0.001 |
| TG.20:4_34:1 | Triglyceride | 0.006 | 0 | <0.001 |
| Citric acid | Organic acid | 0.006 | NS | 0.045 |
| TG.16:0_38.4 | Triglyceride | 0.009 | 0.007 | <0.001 |
| TG.22:5_34:1 | Triglyceride | 0.009 | 0.024 | <0.001 |
| TG.16:0_40:6 | Triglyceride | 0.012 | 0.001 | <0.001 |
| TG.20:4_34:3 | Triglyceride | 0.012 | 0.004 | <0.001 |
| TG.16:0_28:2 | Triglyceride | 0.014 | 0.013 | 0.006 |
| TG.17:0_32:1 | Triglyceride | 0.014 | NS | <0.001 |
| TG.18:2_35:2 | Triglyceride | 0.014 | 0.018 | <0.001 |
| TG.18:0_34:2 | Triglyceride | 0.014 | NS | 0.024 |
| PC aa C38.4 | Phosphatidylcholine | 0.016 | 0.01 | <0.001 |
| TG.20.0_32:3 | Triglyceride | 0.016 | NS | 0.005 |
| PC ae C36.0 | Phosphatidylcholine | 0.018 | NS | <0.001 |
| PC ae C38.2 | Phosphatidylcholine | 0.018 | NS | <0.001 |
| TG.18:2_33:1 | Triglyceride | 0.018 | 0.004 | <0.001 |
| TG.22:6_34:2 | Triglyceride | 0.018 | 0.036 | <0.001 |
| PC ae C38.0 | Phosphatidylcholine | 0.021 | NS | <0.001 |
| CE.20.4 | Cholesteryl ester | 0.023 | 0.033 | <0.001 |
| Acetyl.Lysine | Amino acid derivative | 0.028 | NS | 0.001 |
| PC aa C42.6 | Phosphatidylcholine | 0.029 | NS | <0.001 |
| Oxalic.acid | Organic acid | 0.029 | NS | 0.003 |
| TG.18:1_38:6 | Triglyceride | 0.031 | NS | <0.001 |
| PC aa C38.3 | Phosphatidylcholine | 0.033 | 0.033 | <0.002 |
| CE.14.1 | Cholesteryl ester | 0.037 | NS | 0.002 |
| TG.22:5_32:0 | Triglyceride | 0.037 | 0.004 | <0.001 |
| TG.16:0_33:2 | Triglyceride | 0.039 | 0.023 | <0.001 |
| Indoleacetic.acid | Organic acid | 0.039 | NS | NS |
| PC ae C36.1 | Phosphatidylcholine | 0.040 | NS | <0.001 |
| Acetyl.serine | Amino acid derivative | 0.040 | NS | 0.002 |
| Aconitic.acid | Organic acid | 0.040 | NS | 0.011 |
| PC aa C42.5 | Phosphatidylcholine | 0.041 | 0.029 | <0.001 |
| TG.20:4_36:4 | Triglyceride | 0.041 | 0 | <0.001 |
| TG.17:0_34:3 | Triglyceride | NS | 0.007 | <0.001 |
| PC ae C38.5 | Phosphatidylcholine | NS | NS | <0.001 |
| CE.22.5 | Cholesteryl ester | NS | NS | 0.008 |
| CE.22.6 | Cholesteryl ester | NS | NS | 0.001 |
| CE.20.5 | Cholesteryl ester | NS | 0.038 | <0.001 |
| PC aa C36.0 | Phosphatidylcholine | NS | NS | <0.001 |
| CE.18.3 | Cholesteryl ester | NS | NS | 0.008 |
| TG.20:3_34:0 | Triglyceride | NS | NS | 0.001 |
| Tiglylglycine | Organic acid | NS | 0.002 | <0.001 |
| Serotonin | Neurotransmitter | NS | NS | <0.001 |
| PC aa C36.1 | Phosphatidylcholine | NS | NS | <0.001 |
| TG.22:6_32:1 | Triglyceride | NS | NS | 0.018 |
| TG.17:0_34:1 | Triglyceride | NS | NS | <0.001 |
| Caprylic acid | Fatty acid | NS | NS | 0.034 |
| PC aa C34.1 | Phosphatidylcholine | NS | NS | <0.001 |
| PC ae C34.1 | Phosphatidylcholine | NS | NS | <0.001 |
| C0 | Carnitine | NS | NS | <0.001 |
| TG.18:1_38:7 | Triglyceride | NS | NS | 0.002 |
| PC aa C36.5 | Phosphatidylcholine | NS | NS | <0.001 |
| CE.15.1 | Cholesteryl ester | NS | NS | 0.008 |
| Serine | Amino acid | NS | NS | 0.001 |
| Valine | Amino acid | NS | NS | 0.033 |
| PC ae C38.1 | Phosphatidylcholine | NS | NS | <0.001 |
| TG.16:0_40:7 | Triglyceride | NS | NS | 0.001 |
| PC ae C42.2 | Phosphatidylcholine | NS | NS | 0.001 |
| CE.20.3 | Cholesteryl ester | NS | NS | 0.017 |
| PC aa C32.0 | Phosphatidylcholine | NS | NS | 0.002 |
| TG.20:3_34:2 | Triglyceride | NS | NS | 0.002 |
| PC ae C34.0 | Phosphatidylcholine | NS | NS | 0.002 |
| PC aa C36.4 | Phosphatidylcholine | NS | NS | 0.001 |
| PC ae C38.4 | Phosphatidylcholine | NS | NS | 0.002 |
| Kynurenine | Amino acid derivative | NS | NS | 0.005 |
| PC ae C38.3 | Phosphatidylcholine | NS | NS | <0.001 |
| Methylmalonic acid | Organic acid | NS | NS | 0.025 |
| TG.20:3_36:3 | Triglyceride | NS | NS | 0.034 |
| PC ae C40.1 | Phosphatidylcholine | NS | NS | 0.005 |
| Hippuric acid | Organic acid | NS | NS | 0.034 |
| TG.18:0_38:6 | Triglyceride | NS | NS | <0.001 |
| PC aa C38.0 | Phosphatidylcholine | NS | NS | <0.001 |
| PC aa C36.2 | Phosphatidylcholine | NS | NS | 0.043 |
| Hydroxyglutaric acid | Organic acid | NS | NS | <0.001 |
| PC aa C36.3 | Phosphatidylcholine | NS | NS | 0.017 |
| Acetyl.glycine | Amino acid derivative | NS | NS | <0.001 |
| PC ae C42.4 | Phosphatidylcholine | NS | NS | 0.002 |
| SM.C16.1 | Sphingomyelin | NS | NS | 0.013 |
| PC aa C40.3 | Phosphatidylcholine | NS | NS | <0.001 |
| Carnosine | Dipeptide | NS | NS | <0.001 |
| PC ae C30.1 | Phosphatidylcholine | NS | NS | 0.011 |
| Betaine | Amino acid derivative | NS | NS | 0.006 |
| PC ae C40.2 | Phosphatidylcholine | NS | NS | <0.001 |
| PC aa C40.2 | Phosphatidylcholine | NS | NS | 0.033 |
| PC ae C30.0 | Phosphatidylcholine | NS | NS | 0.021 |
| SM.C16.0 | Sphingomyelin | NS | NS | 0.034 |
| Proline | Amino acid | NS | NS | 0.006 |
| TG.20:4_36:2 | Triglyceride | NS | NS | 0.035 |
| Creatinine | Metabolite | NS | NS | 0.016 |
| Homoarginine | Amino acid | NS | NS | 0.006 |
| Tryptophan | Amino acid | NS | NS | 0.022 |
| Alanine | Amino acid | NS | NS | 0.046 |
| Methylhistidine | Amino acid derivative | NS | NS | 0.021 |

¹Metabolite abbreviations: TG, triglyceride; PC, phosphatidylcholine; ae, acyl-alkyl; aa, diacyl; CE, cholesteryl ester; C0, free carnitine; SM, sphingomyelin; ² NS: not significant.

| **Table S7.** Significant plasma discriminating metabolites identified by univariate and multivariate analyses for the interactive effects of seaweed supplementation and methane emission groups on the plasma metabolome of replacement heifers. | | | |  |
| --- | --- | --- | --- | --- |
| **Analysis method** | | **Significant metabolites ^1^** | |  |
| **ROC ^2^ AUC ^3^** | |  | |  |
|  | | TG.20:1_34:0, Urea, TG.16:0_38:2, TG.20:1_34:2, TG.18:2_28:0, TG.20:0_34:1, TG.20:0_32:3, Oxalic.acid, TG.22:4_34:2, TG.18:1_28:1, TG.14:0_38:4, TG.16:0_35:3, TG.18:2_38:5, TG.14:0_32:2, LysoPC.a.C18.2 | |  |
| **Two-way ANOVA ^4^** | |  | |  |
|  | | TG.16:1_34:1, TG.17:1_36:4, TG.18:2_30:1, TG.16:1_34:2, PC ae C40.5, TG.18:2_32:1, TG.17:1_34:1, PC ae C40.4, Threonic acid, TG.14:0_34:2, TG.16:1_36:4, TG.20:4_32:1, TG.20:1_31:0, PC ae C40.6, TG.16:0_34:4 | |  |
| **Random Forest MDA ^5^** | |  | |  |
|  | | TG.18:3_36:4, TG.18:2_36:2, TG.18:1_36:4, TG.18:2_36:3, PC aa C28.1, Ethylmalonic acid, TG.18:2_36:4, TG.18:3_36:1, TG.18:1_36:2, Hydroxyphenylpyruvic.acid, PC aa C40.6, trans.4.Hydroxyproline, Cis-aconitic acid, TG.18:1_36:3, TG.18:3_36:2 | |  |
| **Pairwise ROC** | |  | |  |
| Seaweed Groups | Methane Emission Groups | | Significant Discriminating Metabolites |  |
| 0.0% vs.0.5% | High, Moderate | | NS ^6^ |  |
| 0.0% vs.1.0% | Low, High, Moderate | | Ethylmalonic.acid, PC ae C42.3, Hippuric.acid, TG.20:3_32:1 |  |
| 0.0% vs.2.0% | High, Moderate | | TG.18:0_32:2, TG.16:0_38:2, TG.16:0_35:3, Hex2Cer.d18.1.16.0, TG.18:1_36:1, TG.20:1_34:0, TG.17:1_32:1, TG.16:1_33:1, TG.14:0_38:4, TG.18:2_28:0, TG.18:1_30:1, TG.16:1_36:1, TG.18:1_34:4, Benzoic.acid, TG.16:1_32:1 |  |
| 0.5% vs.1.0% | Low, Moderate | | Homoarginine |  |
| 0.5% vs.2.0% | Low, High, Moderate | | Sarcosine, Threonine, Propionic.acid, alpha.Ketoglutaric.acid, oxoisocaproic.acid, TG.20:4_32:2, LysoPC a C18.2, Benzoic.acid, Pyruvic.acid, Lactic.acid, Fumaric.acid, Cystathionine, alpha-Ketoisovaleric acid, Hydroxybutyric.acid, Isoleucine |  |
| 1.0% vs.2.0% | Low, High, Moderate | | NS |  |

¹Metabolite abbreviations: TG, triglyceride; LysoPC, lysophosphatidylcholine; PC, phosphatidylcholine; ae, acyl-alkyl; aa, diacyl; Hex2Cer, dihexosylceramide. ²ROC: receiver operating characteristic; ³AUC: area under the curve; ⁴ANOVA: analysis of variance; ⁵MDA: mean decrease accuracy; ⁶NS: not significant.

**Table S8.** KEGG-based pathway annotation of plasma metabolites discriminating Atlantic GRO® seaweed supplementation, methane emission phenotype, and their interaction in replacement beef heifers.

| **Enriched pathway/ pathway annotation** | **Manuscript effect(s) where observed** | **Mapped metabolites ^1^** |
| --- | --- | --- |
| Valine, leucine and isoleucine biosynthesis | Independent seaweed supplementation; Low vs. Moderate methane emission groups; 0.5% vs. 2.0% seaweed comparison across methane groups (interaction) | Valine; Leucine; Isoleucine |
| Valine, leucine and isoleucine degradation | Low vs. Moderate methane emission groups; 0.5% vs. 2.0% seaweed comparison across methane groups (interaction) | alpha-Ketoisovaleric acid; oxoisocaproic.acid; Valine; Leucine; Isoleucine; 3-Hydroxyisovaleric acid; Hydroxyisovaleric acid |
| Pyrimidine metabolism | Independent seaweed supplementation | Cytidine; Deoxycytidine; Thymidine; Uridine |
| Purine metabolism | Overall seaweed x methane emission interaction | Inosine; Xanthosine; Deoxyadenosine; Uric acid; Allantoin |
| Arginine biosynthesis / arginine-related nitrogen metabolism | High vs. Moderate methane emission groups; overall seaweed x methane emission interaction; 0.5% vs. 2.0% seaweed comparison across methane groups (interaction) | Urea; Asymmetric dimethylarginine; Homoarginine; Argininic acid; Guanidinopropionic acid |
| Glycine, serine and threonine metabolism | 0.5% vs. 2.0% seaweed comparison across methane groups (interaction) | Glycine; Serine; Threonine; Sarcosine; N-Acetylserine; N-Acetyl Glycine; Oxalic acid |
| Alanine, aspartate and glutamate metabolism | 0.5% vs. 2.0% seaweed comparison across methane groups (interaction) | Glutamic.acid; N-Acetyl Glutamine; 2-Hydroxyglutaric.acid; Hydroxyglutaric acid; alpha.Ketoglutaric.acid; Pyruvic.acid; Fumaric.acid; N-Acetyl Alanine |
| Cysteine and methionine metabolism | 0.5% vs. 2.0% seaweed comparison across methane groups (interaction) | Methionine sulfoxide; Cystathionine; alpha.Aminobutyric.acid |
| Tyrosine metabolism | 0.5% vs. 2.0% seaweed comparison across methane groups (interaction) | Hydroxyphenylpyruvic.acid; Hydroxyphenyl acetic acid; N-Acetyl.Tyrosine; p-Cresol sulfate |
| Propanoate metabolism | High vs. Moderate methane emission groups | Propionic.acid; Succinic.acid; Pyruvic.acid; Lactic.acid |
| Pyruvate metabolism | 0.5% vs. 2.0% seaweed comparison across methane groups (interaction) | Pyruvic.acid; Lactic.acid |
| Citrate cycle (TCA cycle) | 0.5% vs. 2.0% seaweed comparison across methane groups (interaction) | Citric.acid; Cis-aconitic acid; alpha.Ketoglutaric.acid; Succinic.acid; Fumaric.acid; Malic acid |
| Pantothenate and CoA biosynthesis | Low vs. Moderate methane emission groups | No directly named metabolite/feature from Tables S1, S3, or S6; retained because this enriched pathway was reported in the manuscript. |
| Lipoic acid metabolism | 0.5% vs. 2.0% seaweed comparison across methane groups (interaction) | No directly named metabolite/feature from Tables S1, S3, or S6; retained because this enriched pathway was reported in the manuscript. |
| Glycerolipid metabolism / triacylglycerol metabolism | TG features reported for independent seaweed supplementation, methane emission groups, and interaction analyses | TG.18:0_38:6; TG.18:0_30:1; TG.18:0_36:4; TG.20:1_34:0; TG.20:1_34:2; TG.18:2_28:0; TG.22:4_34:2; TG.18:3_36:4; TG.18:2_36:2; TG.18:2_36:3; TG.14:0_38:4; TG.16:0_38:2; TG.16:0_35:3; TG.18:2_32:1; TG.20:3_32:1 |
| Glycerophospholipid metabolism | PC and LysoPC features reported for independent seaweed supplementation, methane emission groups, and interaction analyses | PC aa C32.2; PC ae C40.5; PC ae C38.6; PC ae C38.2; PC ae C36.5; PC aa C40.6; PC ae C40.4; PC ae C42.3; LysoPC a C18.2; LysoPC a C20.4; LysoPC a C16.0; LysoPC.a.C14.0 |
| Sphingolipid / glycosphingolipid metabolism | Sphingolipid-related features reported in seaweed supplementation and methane emission comparisons | HexCer.d18.1.24.1; Hex2Cer.d18.1.16.0; Hex2Cer.d18.1.14.0; Hex2Cer.d18.1.24.0; Cer.d18.2.24.1; SM.C24.0; SM.(OH).C22.1 |
| Fatty acid oxidation / acylcarnitine-related energy metabolism | Acylcarnitine features reported mainly for independent seaweed supplementation comparisons | C2; C3OH; C3.1 |
| Microbial fermentation and host-microbial co-metabolism | Microbial-derived or host-microbial metabolites reported for independent seaweed supplementation, methane emission groups, and interaction analyses | Trimethylamine; Dimethylamine; Indole.3.propionic.acid; Indolelactic acid; 3.Indoleacetic.acid; p-Cresol sulfate; Hippuric.acid; Benzoic.acid; Caproic.acid; Propionic.acid; Butyric acid/Isobutyric acid |
| Organic acid and central-energy metabolism | Organic acid features reported for methane emission group comparisons and for the seaweed x methane interaction | Ethylmalonic.acid; Oxalic acid; Hydroxybutyric.acid; Hydroxyisobutyric acid; Hydroxyisovaleric acid; 2-Hydroxyglutaric.acid; Hydroxyglutaric acid; Malic acid; Succinic.acid; Citric.acid; Cis-aconitic acid; Fumaric.acid; Pyruvic.acid; Lactic.acid |
| Nitrogen homeostasis and amino-acid derivatives | Nitrogen-related features reported for seaweed supplementation, methane emission groups, and interaction analyses | Urea; Asymmetric dimethylarginine; Homoarginine; Argininic acid; N1-Acetyllysine; N6.Acetyllysine; N-Acetyllysine; N-Acetylproline; N-Acetyl Alanine; N-Acetyl Glutamine |

¹Metabolite abbreviations: TG, triacylglycerol; PC, phosphatidylcholine; LysoPC, lysophosphatidylcholine; aa, diacyl; ae, acyl-alkyl; HexCer, hexosylceramide; Hex2Cer, dihexosylceramide; Cer, ceramide; SM, sphingomyelin; OH, hydroxyl; C2, acetylcarnitine; C3OH, hydroxypropionylcarnitine; C3.1, unsaturated C3 acylcarnitine; CoA, coenzyme A; TCA, tricarboxylic acid.
